# New Triterpenoids from the Leaves of *Heritiera littoralis* and Their Anti-Inflammatory Activity

**DOI:** 10.3390/molecules30010131

**Published:** 2024-12-31

**Authors:** Chenyang Ren, Xiaoqin Liang, Rui Pi, Jiwen Xin, Bo Yang, Qingfang Zheng, Yanqin Li, Jun Li, Yanjun Zhang

**Affiliations:** 1 State Key Laboratory for Chemistry and Molecular Engineering of Medicinal Resources, Key Laboratory for Chemistry and Molecular Engineering of Medicinal Resources (Ministry of Education of China), Collaborative Innovation Center for Guangxi Ethnic Medicine, School of Chemistry and Pharmaceutical Sciences, Guangxi Normal University, Guilin 541004, China; rcy9910@stu.gxnu.edu.cn (C.R.); lxiaoqin20181124@163.com (X.L.); 13278111850@163.com (J.X.); y1903851236@163.com (B.Y.); 18775566850@163.com (Q.Z.); liyanqin1721@163.com (Y.L.); 2 Guangxi Key Laboratory of Green Chemical Materials and Safety Technology, College of Petroleum and Chemical Engineering, Beibu Gulf University, Qinzhou 535000, China; pirui5915862@163.com

**Keywords:** *Heritiera*, *Heritiera littoralis*, heritiera C, heritiera D, anti-inflammatory

## Abstract

Six new triterpenoids, heritieras C–H (**1**–**6**), along with thirteen known triterpenoids (**7**–**19**), were isolated from the leaves of *Heritiera littoralis*. Their structures were identified by spectroscopic analysis, including 1D and 2D nuclear magnetic resonance (NMR), high-resolution electrospray ionization mass spectrometry (HRESIMS), and by comparison with the literature. Anti-inflammatory activity of the isolates was evaluated using the lipopolysaccharide (LPS) stimulated RAW 264.7 cell model. Among the isolated triterpenoid, compounds **1**, **12**, **16**, **17**, and **18** demonstrated inhibitory activity against nitric oxide (NO) release, in which compound **18** exhibited the best activity with an IC_50_ value of 18.13 μM. The potential anti-inflammatory mechanism was investigated using molecular docking. The triterpenoids from *H. littoralis* could be served as potential candidates for the development of new anti-inflammatory agents.

## 1. Introduction

Malvaceae is a family consisting of herbs, subshrubs, shrubs, or trees, with about 245 genera and 4300 species, of which about 59 genera and 264 species are found in China [1,2]. The genus *Heritiera* belongs to the family Malvaceae growing in tropical and subtropical regions of Asia. It consists of 35 species, of which 3 species can be found in provinces of Guangdong, Guangxi, Hainan, and Yunnan in China [3].

The genus Heritiera has a long history for medicinal use in China, especially for folk use [4,5]. *Heritiera littoralis* Dryand, a semi-mangrove plant, has been used as a traditional medicine by the Jing people [6]. This plant has been reported for its wide ranges of activities. The water extract of the bark from *H. littoralis* can be used to treat hematuria, diarrhea, and dysentery. The seeds are used to treat diarrhea. The twigs are served as toothbrushes [7,8]. Previous phytochemical investigations of *H. littoralis* revealed a few chemical compositions, such as flavonoids, terpenoids, phenylpropanoids, lignans, steroids, and triterpenoid saponins [9,10,11,12,13,14]. The constituents from *H. littoralis* have shown an impressive range of biological activities, including antibacterial, antioxidant [15], anti-inflammatory [16,17], and toxicant effects [11].

**Figure 1:**
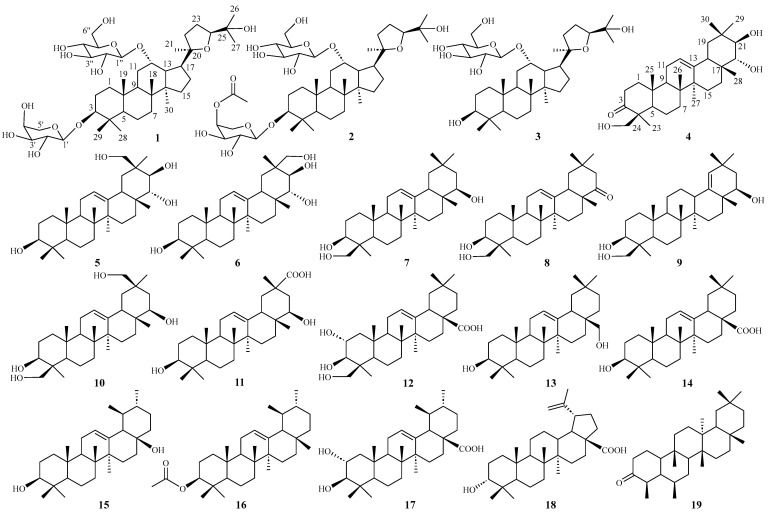
Structures of 1–19 from H. littoralis.

To further explore the anti-inflammatory ingredients from the leaves of *H. littorali*s, the chemical constitutions and pharmacological activities of *H. littoralis* were investigated. Nineteen compounds, including six new triterpenoids, heritieras C–H (**1**–**6**), and thirteen known triterpenoids (**7**–**19**) (Figure 1), were isolated from the extract of the leaves of *H. littoralis*. Furthermore, the isolates were evaluated for their anti-inflammatory activity against the production of the NO in LPS induced RAW 264.7 macrophage cells. The potential anti-inflammatory mechanism was further investigated using molecular docking. Herein, the isolation, purification, and determination of the isolates and their anti-inflammatory activity and molecular docking assay are described.

## 2. Results and Discussion

### 2.1. Elucidation of the Chemical Structures of Heritieras C–H (***1***–***6***)

**Figure 2:**
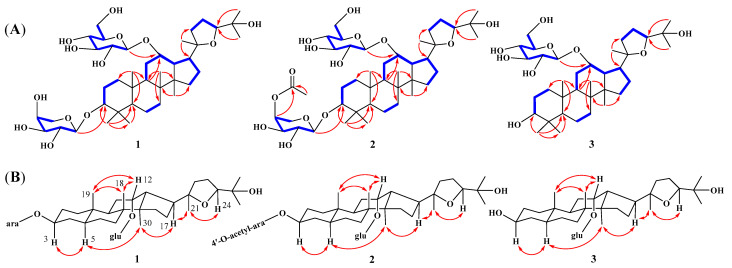
(**A**) ^1^H-^1^H COSY (blue bold bonds) and key HMBC (red arrows) correlations of **1**–**3**. (**B**) ROESY (double dashed arrows) correlations of **1**–**3**.

Compound **1**, a white amorphous powder, had the molecular formula of C_41_H_70_O_13_ determined by HRESIMS *m*/*z* 793.4706 [M + Na]^+^ (calcd. *m*/*z* 793.4714), indicating seven unsaturated degrees. The ^1^H-NMR spectrum (Table 1) of **1** exhibited eight methyl singlets at *δ*_H_ 0.63 (3H, s, H-30), 0.95 (3H, s, H-28), 1.03 (3H, s, H-18), 1.16 (3H, s, H-21), 1.26 (3H, s, H-29), 1.41 (6H, s, H-19, 26), 1.48 (3H, s, H-27), and two anomeric protons, *δ*_H_ 4.72 (1H, d, *J* = 6.4 Hz) and *δ*_H_ 5.14 (1H, d, *J* = 8.0 Hz), as well as overlapping signals belonging to sugar moieties around *δ*_H_ 3.76−4.54. The ^13^C-NMR, DEPT, and HSQC spectra of **1** displayed 41 carbon signals, including 30 carbons attributed to a triterpenoid skeleton [three oxygenated methines (*δ*_C_ 78.0, 81.8, 84.7), and two oxygenated quaternary carbon (*δ*_C_ 71.7, 86.9), four methines (*δ*_C_ 41.6, 50.0, 51.4, 54.4), four quaternary carbon (*δ*_C_ 38.5, 41.7, 41.9, 50.5), nine methylenes (*δ*_C_ 18.8, 22.3, 26.7, 27.1, 32.0, 34.6, 35.0, 36.1, 36.8), eight methyls (*δ*_C_ 17.2, 17.4×2, 23.6, 25.0, 26.7, 28.1, 30.5)] and the other 11 carbons assigned to two sugar moieties [one L-arabinopyranose (*δ*_C_ 66.8, 69.6, 72.9, 75.2, 102.3) and one D-glucopyranose (*δ*_C_ 63.9, 72.9, 75.8, 78.3, 79.1, 102.7)]. The ^1^H−^1^H COSY data revealed the partial structures –CH_2_(1)−CH_2_(2)−CH(3)−, –CH_2_(6)−CH_2_(7)−, −CH(9)−CH_2_(11)−CH(12)−CH(13)−CH(17)−CH_2_(16)−CH_2_(15)− and -CH_2_(22)−CH_2_(23)−CH_3_(24)− (Figure 2A). Detailed analysis of the abovementioned NMR data showed that the data of **1** was highly similar to those of ocotillol-II [18], indicating that **1** has the same basic dammarane triterpenoid skeleton as ocotillol-II. The differences from ocotillol-II are that **1** has an L-arabinopyranose connected at position 3 and a D-glucopyranose connected at position 12. Furthermore, the HMBC correlations from the anomeric protons *δ*_H_ 4.72 (H-1′) to *δ*_C_ 81.8 (C-3) and *δ*_H_ 5.14 (H-1″) to *δ*_C_ 78.0 (C-12) suggested that the two sugars are situated at C-3 and C-12, respectively (Figure 2A). After acid hydrolysis of **1** using 1 M HCl, one set of carbon signals for an *α*-L-arabinopyranose and another set of carbon signals for a *β*-D-glucopyranose were identified by comparison on HPLC with authentic samples [19] (Figure S51). In addition, the relative stereochemistry was deduced from ROESY spectrum (Figure 2B). In the ROESY spectrum, the correlations between H-3/H-5/H_3_-30/H-17 and H-12/H_3_-18/H_3_-19 revealed that H-3, 5, 30, and 17 were located on one side, whereas H-12, 18, and 19 were on the other side. According to biosynthetic characteristics of dammarane trierpenoid, H-18 and 19 are *β*-orientation, whereas H-30 is *α*-orientation [20]. Consequently, it can be deduced that H-3 is *α*-orientation and H-12 is *β*-orientation. In addition, the absolute configurations at C-20 and C-24 were determined to be *R* and *S*, respectively, by comparing with the ^13^C NMR chemical shift in analogous epoxydammaranes [21,22]. Thus, the structure of **1** was deduced as (20*R*,24*S*)-20,24-epoxy-3*β*,12*α*,25-trihydoxy-12-*O*-*β*-D-glucopyranosyldammarane-3-*O*-*α*-L-arabinopyranoside (**1**), named heritiera C (Figure 1).

Compound **2** was isolated as white amorphous powder. Its molecular formula was established as C_43_H_72_O_14_ by HRESIMS *m*/*z* 835.4820 [M + Na]^+^ (calcd. *m*/*z* 835.4820), which is 42 Da more than that of **1**. Extensive analysis of the NMR data (Table 1) revealed that **2** shares almost the same structure as **1**, the difference is one more acetyl group (*δ*_C_ 170.7, 20.9) at position 4′ in **2**. The location of the acetyl group was confirmed by the HMBC correlations from *δ*_H_ 5.41 (H-4′) to *δ*_C_ 170.7 (-OOCCH_3_) (Figure 2A). The sugar units were determined to be *α*-L-arabinopyranose and *β*-D-glucopyranose based on the hydrolysis of **2** with 1 M HCl and the assays of HPLC (Figure S51). Moreover, the diagnostic ROESY correlations of H-3/H-5/H-30/H-17 suggested that H-3 is *α*-oriented, while the ROESY correlations of H-12/H-18/H-19 indicated that H-12 is *β*-oriented (Figure 2B). In addition, to determine the absolute configuration of the chiral centers C-20 and C-24 in compound **2**, the carbon signals at *δ*_C_ 86.9 (C-20) and 84.6 (C-24) were compared with those of analogous dammaranes [21,22], suggesting that the chiral centers are 20*R* and 24*S*, respectively. Therefore, the structure of **2** was determined as (20*R*,24*S*)-20,24-epoxy-3*β*,12*α*,trihydoxy-12-*O*-*β*-D-glucopyranosyldammarane-3-*O*-[4′-*O*-acetyl]-*α*-L-arabinopyran-oside (**2**), named heritiera D (Figure 1).

Compound **3** was isolated as white amorphous powder. Its molecular formula was established as C_36_H_62_O_9_ by HRESIMS *m*/*z* 661.4291 [M + Na]^+^ (calcd. *m*/*z* 661.4292), which is 132 Da less than that of **1**. Extensive analysis of the NMR data (Table 1) revealed that **3** shares almost the same structure as **1**, the only difference is that compound **3** is one less arabinopyranose group at position 3 than **1**. Moreover, the HMBC correlations from *δ*_H_ *δ*_H_ 5.17 (H-1″) to *δ*_C_ 77.2 (C-12) suggested that the D-glucopyranose are situated at C-12 (Figure 2A). The sugar unit was determined to be *β*-D-glucopyranose based on the hydrolysis of **3** with 1 M HCl and the assays of HPLC (Figure S49). Furthermore, the *β*-orientations of H-12 were then verified by the ROESY peaks of H-12/H-18/H-19 (Figure 2B). In addition, to determine the absolute configuration for chiral centers C-20/24 of **3**, the comparisons of the carbon signals of compound **3** at *δ*_C_ 86.9 (C-20) and 84.6 (C-24) with those of analogous dammaranes suggested its chiral centers to be 20*R* and 24*S* [21,22]. Consequently, the structure of **3** was identified as (20*R*,24*S*)-20,24-epoxy-3*β*,12*α*,25-trihydoxy −12-*O*-*β*-D-glucopyranosyldammarane (**3**), named heritiera E (Figure 1).

**Figure 3:**
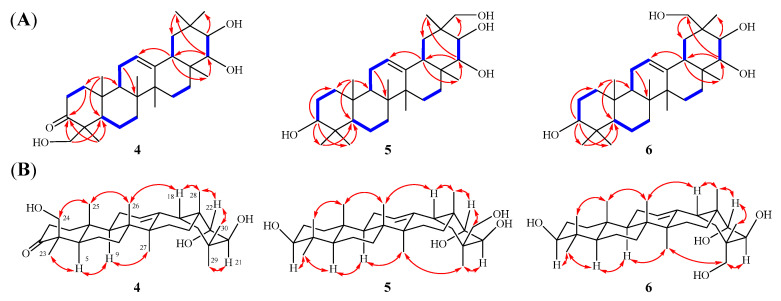
(**A**) ^1^H-^1^H COSY (blue bold bonds) and key HMBC (red arrows) correlations of **4**–**6**. (**B**) NOESY (double dashed arrows) correlations of **4**–**6**.

Compound **4** was isolated as white amorphous powder. Its molecular formula was established as C_30_H_48_O_4_ by HRESIMS *m*/*z* 473.3633 [M + H]^+^ (calcd. *m*/*z* 473.3631), suggesting seven unsaturated degrees. The ^1^H-NMR spectrum (Table 2) of **4** exhibited seven methyl singlets at *δ*_H_ 1.06 (3H, s, H-26), 1.17 (3H, s, H-25), 1.22 (3H, s, H-27), 1.25 (3H, s, H-30), 1.29 (3H, s, H-28), 1.45 (3H, s, H-29), and 1.50 (3H, s, H-23), and one olefinic proton *δ*_H_ 5.35 (1H, t, *J* = 3.6 Hz, H-12). Its ^13^C NMR spectrum (Table 2) revealed the presence of 30 carbon resonances, which were classified by DEPT and HSQC spectra, including one carbonyl carbon (*δ*_C_ 214.9), one double bond (*δ*_C_ 122.6, 145.0), three oxygenated carbons (*δ*_C_ 65.6, 75.0, 80.0), and seven methyl carbons (*δ*_C_ 16.1, 17.4, 21.2, 21.7, 22.7, 27.0, 31.9). Extensive analysis of the NMR data (Table 2) revealed that **4** shares almost the same structure as soyasapogenol A [23]. The difference between these two compounds is that soyasapogenol A has four oxygenated carbon signals, while **4** has three oxygenated carbon signals and a carbonyl carbon signal (*δ*_C_ 214.9), suggesting that one oxygenated carbon in soyasapogenol A has been oxidated to a carbonyl carbon in **4**. In the ^1^H−^1^H COSY spectrum of **4**, the following partial structures were verified as –CH_2_(1)−CH_2_(2)−, –CH(5)−CH_2_(6)−CH_2_(7)−, –CH(9)−CH_2_(11)−CH(12)−, –CH_2_(15)−CH_2_(16)−, –CH(18)−CH_2_(19)− and –CH(21)−CH(22)− (Figure 3A). Furthermore, the key HMBC correlations from *δ*_H_ 2.37 and 2.83 (H-2), 1.50 (H_3_-23), as well as 3.89 and 4.37 (H-24) to *δ*_C_ 214.9 (C-3) confirmed that the carbonyl carbon was situated at C-3 (Figure 3A). In addition, the relative configurations of **4** were determined by NOESY spectrum (Figure 3B). The key correlations between H-5/H-9/H-21/H_3_-23/H_3_-27/H_3_-29 indicate that H-5, H-9, H-21, H_3_-23, H_3_-27, and H_3_-29 are on the same side, suggesting that H-5, H-9, H-21, H_3_-23, H_3_-27, and H_3_-29 are *α*-orientation. The correlations between H-18/H-22/H_2_-24/H_3_-25/H_3_-26/H_3_-28/H_3_-30 suggest that H-18, H-22, H_2_-24, H_3_-25, H_3_-26, H_3_-28, and H_3_-30 are on the same side, indicating that H-18, H-22, H_2_-24, H_3_-25, H_3_-26, H_3_-28, and H_3_-30 are *β*-orientation. Consequently, according to the above analysis and the biosynthetic characteristics of oleanane triterpenoid [20], the absolute configuration of **4** can be determined as 21*β*,22*α*,24-trihydroxy-3-oxo-olean-12-ene (**4**), named heritiera F (Figure 1).

Compound **5** was isolated as white amorphous powder. Its molecular formula was established as C_30_H_50_O_4_ by HRESIMS *m*/*z* 497.3606 [M + Na]^+^ (calcd. *m*/*z* 497.3607), suggesting six unsaturated degrees. The ^1^H-NMR spectrum (Table 2) of **5** exhibited seven methyl singlets at *δ*_H_ 0.96 (3H, s, H-25), 1.06 (3H, s, H-27), 1.08 (3H, s, H-23), 1.26 (3H, s, H-24), 1.31 (3H, s, H-26), 1.34 (3H, s, H-28) and 1.44 (3H, s, H-29), and one olefinic proton *δ*_H_ 5.36 (1H, t, *J* = 3.4 Hz, H-12). Its ^13^C NMR spectrum (Table 2) revealed the presence of 30 carbon resonances, which were classified by DEPT and HSQC spectra, including one double bond (*δ*_C_ 123.4, 144.8), four oxygenated carbons (*δ*_C_ 67.4, 76.6, 78.4, 79.8), and seven methyl carbons (*δ*_C_ 16.1, 16.9, 17.4, 22.8, 27.1, 27.4, 29.1). Extensive analysis of the NMR data (Table 2) revealed that **5** shares almost the same structure as abrisapogenol L [24], the difference being in that **5** possesses a methyl group at C-24 instead of a CH_2_OH at C-24 in abrisapogenol L. In the ^1^H−^1^H COSY spectrum of **5**, the partial structures revealed are as follows: –CH_2_(1)−CH_2_(2)−CH(3)−, –CH(5)−CH_2_(6)−CH_2_(7)−, –CH(9)−CH_2_(11)−CH(12)−, –CH_2_(15)−CH_2_(16)−, –CH(18)−CH_2_(19)− and –CH(21)−CH(22)− (Figure 3A). In addition, the key HMBC correlations from *δ*_H_ 3.46 (H-3) and 0.88 (H-5) to *δ*_C_ 16.9 (C-23) and 29.1 (C-24) confirmed that two methyl groups of C-23 and C-24 were linked to C-4 in **5**. (Figure 3A). Furthermore, the relative configurations of **5** were determined by NOESY spectrum (Figure 3B). In the NOESY spectrum of **5,** the correlations of H-3/H-5/H-9/H-21/H_3_-27/H_3_-29 suggest that H-3, H-5, H-9, H-21, H_3_-27 and H_3_-29 are on the same side, indicating H-3, H-5, H-9, H-21, H_3_-27 and H_3_-29 are α-orientation. The NOESY correlations of H-18/H-22/H_3_-28/H_2_-30 suggest that H-18, H-22, H_3_-28 and H_2_-30 are on the same side, indicating H-18, H-22, H_3_-28 and H_2_-30 are *β*-orientation. According to the above analysis, the structure of **5** was deduced as 3*β*,21*β*,22α,30-tetrahydroxy-olean-12-ene (**5**), named heritiera G (Figure 1).

Compound **6** was isolated as white amorphous powder. Its molecular formula was established as C_30_H_50_O_4_ by HRESIMS *m*/*z* 497.3596 [M + Na]^+^ (calcd. *m*/*z* 497.3607), which is the same as that of **5**. The 1D NMR data (Table 1) for **5** and **6** are highly similar, the difference being in C-21 (*δ*_H_ 4.51, *δ*_C_ 70.8), C-29 (*δ*_H_ 3.66 and 4.07, *δ*_C_ 72.0) and C-30 (*δ*_H_ 1.54, *δ*_C_ 17.9) in **6** instead of C-21 (*δ*_H_ 4.15, *δ*_C_ 76.6), C-29 (*δ*_H_ 1.44, *δ*_C_ 27.4), and C-30 (*δ*_H_ 4.33 and 4.42, *δ*_C_ 67.4) in **5**. From the above analysis, it can be inferred that **6** possesses a methyl group at C-29 and a CH_2_OH at C-30 instead of a CH_2_OH at C-29 and a methyl group at C-30. In the NOESY spectrum of **6**, the correlations of H-3/H-5/H-9/H_α_-16/H-21/H_3_-27/H_2_-29 suggest that H-3, H-5, H-9, H_α_-16, H-21, H_3_-27, and H_2_-29 are on the same side, indicating H-3, H-5, H-9, H_α_-16, H-21, H_3_-27, and H_2_-29 are α-orientation. The NOESY correlations of H-18/H-22/H_3_-28/H_3_-30 suggest that H-18, H-22, H_3_-28, and H_3_-30 are on the same side, indicating H-18, H-22, H_3_-28, and H_3_-30 are *β*-orientation (Figure 3B). Thus, the structure of **6** was deduced as 3*β*,21*β*,22α,29-tetrahydroxyl-olean-12-ene (**6**), named heritiera H (Figure 1).

By comparing the measured NMR (^1^H and ^13^C) and MS data to those reported in the literature, the known triterpenoids were identified as soyasapogenol B (**7**) [25], soyasapogenol E (**8**) [26], soyasapogenol H (**9**) [27], wistariasapogenol B (**10**) [28], triptotriterpenic acid B (**11**) [29], 2*α*,3*β*,24-trihydroxy olea-12-en-28-oic acid (**12**) [30], erythrodiol (**13**) [31], oleanolic acid (**14**) [32], uvaol (**15**) [33], *β*-amyrin acetate (**16**) [34], 2*α*-hydroxy ursolic acid (**17**) [35], betulinic acid (**18**) [13], and friedelin (**19**) [36] (Figure 1).

### 2.2. Anti-Inflammatory Assay of the Isolates

Cell viability was measured by an MTT assay. The RAW 264.7 cell viability assays showed that the survival rate remained greater than 90% after treatment with all isolates at different concentrations from 0 to 50 μM. All isolates were evaluated for their anti-inflammatory effects on the production of NO in the RAW 264.7 macrophage cell line exposed to the inflammatory stimulus by LPS. Dexamethasone was used as a positive control. The effects of active compounds (IC_50_ < 50 μM) on the production of NO by LPS-induced RAW 264.7 cells are shown in Table 3.

The results showed that compound **18** substantially inhibited the release of NO, with IC_50_ value of 18.13 μM. The value is slightly lower than that of positive control, dexamethasone, with IC_50_ value of 16.37 μM. Compounds **1**, **12**, **16,** and **17** showed moderate effects with IC_50_ values of 47.12, 45.31, 39.98, and 25.23 μM, respectively. Compounds **2**–**11**, **15**, and **19** showed no significant effects against LPS-induced nitric oxide production in RAW 264.7 macrophages.

### 2.3. Predicted Binding Modes of Compounds and iNOS, COX-2 Using Molecular Docking Analysis

NO serves as an indicator of inflammatory response, and elevated levels of NO within tissues signify the overexpression of critical proteins, iNOS and COX-2 [37,38]. Specifically, iNOS plays a role in catalyzing the production of NO within the inflammatory signaling pathway [39]. Studies have shown that the interaction between iNOS/COX-2 and certain small molecules can halt the inflammatory process and prevent the overproduction of downstream inflammatory mediators [40]. In an effort to elucidate the potential mechanisms behind NO inhibition, the binding interactions between bioactive compounds and iNOS/COX-2 were examined.

**Figure 4:**
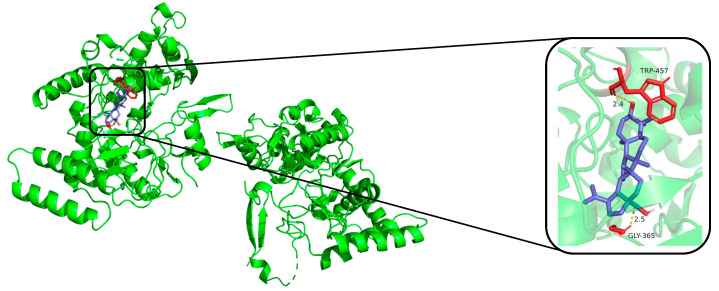
Molecular docking result of compound **18** with iNOS protein. Molecular docking simulation obtained at lowest energy conformation, highlighting potential hydrogen contacts of compound **18** (iNOS protein is shown in green, compound **18** are in purple). For clarity, only interacting residues are labeled. Hydrogen bonds between amino acid residues and compounds are represented by yellow dashed lines. These figures were created by PyMOL (3.0.0).

**Figure 5:**
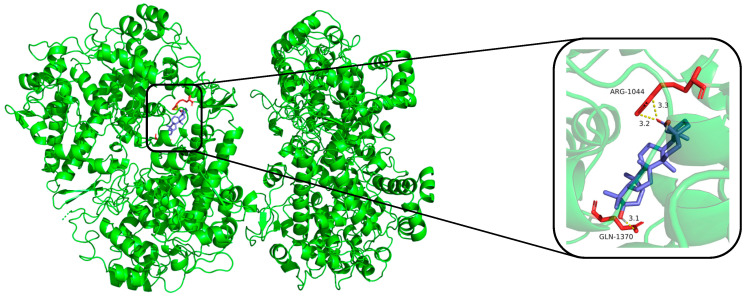
Molecular docking result of compound **18** with COX-2 protein. Molecular docking simulation obtained at lowest energy conformation, highlighting potential hydrogen contacts of compound **18** (COX-2 protein is shown in green, compound **18** are in purple). For clarity, only interacting residues are labeled. Hydrogen bonds between amino acid residues and compounds are represented by yellow dashed lines. These figures were created by PyMOL.

The compounds (**1**, **12**, **16**, **17**, **18**, and dexamethasone) were subjected to molecular docking. Results from molecular docking study revealed that compounds **1**, **12**, **16**, **17**, **18**, and dexamethasone had strong interactions with the iNOS/COX-2 protein (Figure 4, Figure 5 and Figures S53–S62) and the binding residues and the logarithms of free binding energies were collated in Tables S1 and S2. The results show that **18** docks effectively into the active sites of iNOS and COX-2, with binding energies of −9.0 kcal/mol, which is comparable to those of dexamethasone (positive control, with binding energies of −9.5 and −9.6 kcal/mol, respectively). Further simulations were conducted to model the docking sites of compound **18** with iNOS and COX-2. The results show that compound **18** binds to the amino acid residues TRP-457 and GLY-365 of the iNOS protein through hydrogen bonds, and to the amino acid residues ARG-1044 and GLN-1370 of the COX-2 protein through hydrogen bonds as well.

Comparison their IC_50_ values with the results of molecular docking, it was discovered that the IC_50_ values of the active compounds correlate with the trends observed in molecular docking study. Compounds with higher activity have lower Logarithms of free binding energies (FBE). For example, compound **18** and dexamethasone form hydrogen bonds with the amino acid residue GLY-365 in the iNOS protein to show lower IC_50_ values and free binding energies. Meanwhile, compounds **12, 17**, and **18** all feature an α-oriented hydroxyl group (OH) on ring A and demonstrate excellent activities. They are likely the critical factor contributing to their activities.

## 3. Experimental

### 3.1. General Experimental Procedures

UV spectra were acquired with a TU-1901 spectrophotometer. NMR experiments were conducted on Bruker Advance 600 or 500 MHz spectrometers. Optical rotations were acquired based on a JASCO P-2000 polarimeter. HRESIMS were recorded on Agilent 6545 Q-TOF LC-MS mass spectrometer. Semi-preparative HPLC separations were conducted on an Aglient 1260 instrument equipped with a DAD detector and a C_18_ column; analytical HPLC was conducted on an Aglient 1260 instrument equipped with a DAD detector and an Aglient C_18_ column. OD values of 96-well were measured with an imark Bio-Rad plate microplate reader.

### 3.2. Plant Material

The dried leave (5.0 kg) of *H. littorali*s was obtained from Beilun Estuary in Guangxi Province, China, in September 2018 and identified by professor Qiuping Zhong (Guangxi Institute of Botany). A voucher specimen (No. ID-20180910) is deposited at the Guangxi key laboratory of green chemical materials and safety technology, Beibu Gulf University, China.

### 3.3. Extraction and Isolation

Dried leaves (5.0 kg) of *H. littorali*s were extracted with H_2_O-EtOH (20 L each, 25:75) under the extraction tank (4 h each), The filtrates were combined and dried under reduced pressure to yield a residue (0.52 kg). The concentrates were suspended in H_2_O (1 L) and partitioned with EtOAc (5 × 1 L) and *n*-BuOH fraction (5 × 1 L). The EtOAc extract (233 g) was eluted by silica gel CC (200~300 mesh) using petroleum ether (PE) and ethyl acetate (95:5 to 50:50 *v*/*v*) as eluents and six fractions (Frs. 1–6) were obtained based on TLC assay. Fr. 2 (43.1 g) was subjected to silica gel CC (200~300 mesh) and eluted sequentially with PE-CH_2_Cl_2_ (100:0, 90:10, 80:20, 50:50, and 0:100, *v*/*v*) to yield five fractions (Fr. 2.1−Fr. 2.5). Fr. 2.3 was purified by recrystallization to yield **19** (12.6 mg). Fr. 2.4 was further separated using Sephadex LH-20 (PE:CH_2_Cl_2_:MeOH = 1:2:1, *v*/*v*/*v*) to obtain compound **16** (5.2 mg). Six fractions (Fr. 3.1−Fr. 3.6) were obtained by further separation of Fr. 3 (24.3 g) on an ODS column eluted with H_2_O-MeOH (40:60 to 0:100, *v*/*v*). Fr. 3.3 was subjected to silica gel CC (200~300 mesh) and eluted sequentially with CH_2_Cl_2_-MeOH (100:0, 98:2, 96:4, 94:6, 90:10, and 1:1, *v*/*v*) to yield five fractions (Fr. 3.3.1−Fr. 3.3.5), according to TLC monitoring. Fr. 3.3.2 was purified by semipreparative HPLC (5 μm, 9.3 × 250 mm, 2.5 mL/min, 197 nm), eluted with H_2_O-MeCN (27:73, *v*/*v*) to yield **11** (t_R_ 14.2 min, 7.3 mg), **13** (t_R_ 23.8 min, 9.4 mg), and **14** (t_R_ 32.9 min, 12.3 mg). After purification over Sephadex LH-20 CC eluted with MeOH, Fr. 3.3.3 was purified by semipreparative HPLC (5 μm, 9.3 × 250 mm, 2.5 mL/min, 197 nm), to obtain compounds **4** (t_R_ 16.7 min, 5.6 mg), **7** (t_R_ 20.1 min, 39.1 mg), and **9** (t_R_ 28.6 min, 4.6 mg). Fr. 3.4 was purified by semipreparative HPLC (5 μm, 9.3 × 250 mm, 2.5 mL/min, 210 nm), eluted with H_2_O-MeOH (15:85, *v*/*v*) to yield **18** (t_R_ 36.7 min, 8.6 mg). Fr.4 (20.7 g) was further separated using Sephadex LH-20 (MeOH) to yield five fractions (Fr. 4.1−Fr. 4.4). Fr. 4.1 (6.1 g) was submitted to ODS column eluted with H_2_O-MeOH (40:60 to 10:90, *v*/*v*) to give **12** (t_R_ 15.4 min, 7.2 mg), **15** (t_R_ 18.6 min, 6.5 mg), and **17** (t_R_ 26.1 min, 12.1 mg). Fr. 4.2 (3.5 g) was submitted to Sephadex LH-20 column eluted with MeOH to give **8** (20.1 mg). Compounds **5** (t_R_ 16.7 min, 4.8 mg), **6** (t_R_ 25.2 min, 13.7 mg), and **10** (t_R_ 15.4 min, 54.0 mg) were afforded by semipreparative HPLC purification of Fr. 4.3 (5 μm, 9.3 × 250 mm, 2.5 mL/min, 196 nm), eluted with H_2_O-MeCN (23:77, *v*/*v*). Fr. 4.4 purified by semipreparative HPLC (H_2_O:MeOH = 40:60, *v*/*v*) separations to yield **3** (t_R_ 23.5 min, 5.2 mg). Fr. 5 (23.1 g) was purified successively by ODS column eluted with H_2_O-MeOH (50:50 to 10:90, *v*/*v*) and a Sephadex LH-20 column (H_2_O:MeOH = 80:20, *v*/*v*) to give **1** (8.2 g) and **2** (4.5 mg).

### 3.4. Characterization of the Isolates

Heritiera C (**1**): white amorphous powder; [*α*${]}_{D}^{25}$ − 10.7 (*c* 0.10, MeOH); HRESIMS *m*/*z* 793.4914 [M + Na]^+^, calcd for C_41_H_70_O_13_Na). For ^1^H (pyridine-*d*_5_, 600 MHz) and ^13^C NMR (pyridine-*d*_5_, 150 MHz) spectroscopic data, see Table 1; all significant data are given in the electronic Supplementary Materials (Figures S1−S8).

Heritiera D (**2**): white amorphous powder; [*α*${]}_{D}^{25}$ − 23.5 (*c* 0.10, MeOH); HRESIMS *m*/*z* 835.4820 ([M + Na]^+^, calcd for C_43_H_72_O_14_Na). For ^1^H (pyridine-*d*_5_, 600 MHz) and ^13^C NMR (pyridine-*d*_5_, 150 MHz) spectroscopic data, see Table 1; all significant data are given in the Supplementary Materials (Figures S9−S16).

Heritiera E (**3**): white amorphous powder; [*α*${]}_{D}^{25}$ − 17.2 (*c* 0.10, MeOH); HRESIMS *m*/*z* 661.4291 ([M + Na]^+^, calcd for C_36_H_62_O_9_Na). For ^1^H (pyridine-*d*_5_, 600 MHz) and ^13^C NMR (pyridine-*d*_5_, 150 MHz) spectroscopic data, see Table 1; all significant data are given in the Supplementary Materials (Figures S17−S24).

Heritiera F (**4**): white amorphous powder; [*α*${]}_{D}^{25}$ + 15.4 (*c* 0.10, MeOH); HRESIMS *m*/*z* 473.3633 [M + H]^+^, calcd for C_30_H_48_O_4_). For ^1^H (pyridine-*d*_5_, 600 MHz) and ^13^C NMR (pyridine-*d*_5_, 150 MHz) spectroscopic data, see Table 2; all significant data are given in the electronic Supplementary Materials (Figures S25−S32).

Heritiera G (**5**): white amorphous powder; [*α*${]}_{D}^{25}$ + 27.4 (*c* 0.10, MeOH); HRESIMS *m*/*z* 497.3607 ([M + Na]^+^, calcd for C_30_H_50_O_4_Na). For ^1^H (pyridine-*d*_5_, 600 MHz) and ^13^C NMR (pyridine-*d*_5_, 150 MHz) spectroscopic data, see Table 2; all significant data are given in the Supplementary Materials (Figures S33−S40).

Heritiera H (**6**): white amorphous powder; [*α*${]}_{D}^{25}$ + 20.2 (*c* 0.10, MeOH); HRESIMS *m*/*z* 497.3596 ([M + Na]^+^, calcd for C_30_H_50_O_4_Na). For ^1^H (pyridine-*d*_5_, 600 MHz) and ^13^C NMR (pyridine-*d*_5_, 150 MHz) spectroscopic data, see Table 2; all significant data are given in the Supplementary Materials (Figures S41−S48).

Soyasapogenol B (7): colorless crystals; ^1^H NMR (400 MHz, CDCl_3_) *δ*: 5.14 (1H, t, *J* = 3.7 Hz, H-12), 4.10 (1H, d, *J* = 11.0 Hz, H-3), 3.49 (1H, s, H-22), 1.13 (3H, s, H-23), 1.00 (3H, s, H-25), 0.92 (3H, s, H-26), 0.84 (3H, s, H-27), 0.82 (3H, s, H-28), 0.80 (3H, s, H-29), 0.75 (3H, s, H-30); ^13^C NMR (100 MHz, CDCl_3_) *δ*: 38.3 (C-1), 25.7 (C-2), 80.4 (C-3), 42.0 (C-4), 55.8 (C-5), 18.4 (C-6), 33.1 (C-7), 39.5 (C-8), 47.7 (C-9), 37.3 (C-10), 23.6 (C-11), 122.3 (C-12), 143.7 (C-13), 42.3 (C-14), 28.3 (C-15), 27.2 (C-16), 36.6 (C-17), 45.0 (C-18), 46.0 (C-19), 30.4 (C-20), 41.1 (C-21), 76.3 (C-22), 22.4 (C-23), 64.5 (C-24), 16.1 (C-25), 16.8 (C-26), 25.0 (C-27), 28.4 (C-28), 32.2 (C-29), 19.6 (C-30).

Soyasapogenol E (**8**): white amorphous powder; ^1^H NMR (600 MHz, CDCl_3_) *δ*: 5.29 (1H, t, *J* = 3.7 Hz, H-12), 4.20 (1H, d, *J* = 11.1 Hz, H-24), 3.44 (1H, dd, *J* = 11.8, 4.5 Hz, H-24), 3.34 (1H, d, *J* = 11.1 Hz, H-3), 1.25 (3H, s, H-27), 1.22 (3H, s, H-23), 0.99 (6H, s, H-26, 29), 0.93 (3H, s, H-30), 0.89 (3H, s, H-25), 0.85 (3H, s, H-28); ^13^C NMR (150 MHz, CDCl_3_) *δ*: 38.5 (C-1), 27.7 (C-2), 80.9 (C-3), 42.9 (C-4), 55.9 (C-5), 18.5 (C-6), 33.0 (C-7), 39.8 (C-8), 47.7 (C-9), 36.8 (C-10), 23.9 (C-11), 123.7 (C-12), 141.7 (C-13), 41.9 (C-14), 25.4 (C-15), 27.3 (C-16), 47.7 (C-17), 47.7 (C-18), 46.8 (C-19), 34.3 (C-20), 51.0 (C-21), 217.3 (C-22), 25.5 (C-23), 22.5 (C-23), 64.6 (C-24), 16.2 (C-25), 16.8 (C-26), 25.2 (C-28), 32.1 (C-29), 20.7 (C-30).

Soyasapogenol H (**9**): colorless crystals; ^1^H NMR (600 MHz, pyridine-*d*_5_) *δ*: 4.98 (1H, s, H-19), 4.52 (1H, d, *J* = 11.0 Hz, H-24), 4.00 (1H, dd, *J* = 12.3, 3.8 Hz, H-22), 3.69 (1H, dd, *J* = 11.0, 4.3 Hz, H-3), 2.39 (1H, d, *J* = 11.6 Hz, H-24), 1.54 (3H, s, H-23), 1.34 (3H, s, H-30), 1.14 (3H, s, H-29), 1.08 (6H, s, 25, 26), 0.92 (3H, s, H-28), 0.87 (3H, s, 27); ^13^C NMR (150 MHz, pyridine-*d*_5_) *δ*: 39.6 (C-1), 28.1 (C-2), 80.5 (C-3), 43.7 (C-4), 56.9 (C-5), 19.5 (C-6), 35.5 (C-7), 40.7 (C-8), 52 (C-9), 37.7 (C-10), 22.0 (C-11), 27.3 (C-12), 39.1 (C-13), 43.3 (C-14), 29.0 (C-15), 35.7 (C-16), 41.3 (C-17), 143.3 (C-18), 129.7 (C-19), 34.1 (C-20), 42.7 (C-21), 75.8 (C-22), 23.9 (C-23), 64.9 (C-24), 18.9 (C-25), 17.8 (C-26), 15.4 (C-27), 16.6 (C-28), 32.5 (C-29), 30.5 (C-30).

Wistariasapogenol B (**10**): white amorphous powder; ^1^H NMR (600 MHz, pyridine-*d*_5_) *δ*: 3.95 (2H, m, H-24, 29), 3.81 (4H, m, H-24, 29), 3.73 (1H, m, H-22), 3.69 (1H, dd, 1.5, 5.9, H-3), 1.58 (3H, s, H-30), 1.29 (3H, s, H-28), 1.25 (3H, s, H-27), 1.09 (3H, s, H-23), 1.01 (3H, s, H-26), 0.94 (3H, s, H-25); ^13^C NMR (150 MHz, pyridine-*d*_5_) *δ*_C_ 39.3 (C-1), 28.8 (C-2), 80.5 (C-3), 43.6 (C-4), 56.7 (C-5), 19.5 (C-6), 33.9 (C-7), 40.4 (C-8), 48.5 (C-9), 37.4 (C-10), 24.4 (C-11), 123.1 (C-12), 145.0 (C-13), 42.7 (C-14), 26.8 (C-15), 28.8 (C-16), 38.5 (C-17), 45.6 (C-18), 42.4 (C-19), 36.3 (C-20), 39.3 (C-21), 75.6 (C-22), 24.4 (C-23), 65.0 (C-24), 16.6 (C-25), 17.4 (C-26), 26.2 (C-27), 21.7 (C-28), 70.6 (C-29), 28.9 (C-30).

Triptotriterpenic acid B (**11**): white amorphous powder; ^1^H NMR (600 MHz, pyridine-*d*_5_) *δ*: 5.44 (1H, t, *J* = 3.6 Hz, H-12), 4.04 (1H, dd, *J* = 7.4, 3.2 Hz, H-22), 3.46 (1H, dd, *J* = 11.2, 4.9 Hz, H-3), 1.30 (3H, s, H-30), 1.27 (3H, s, H-28), 1.26 (3H, s, H-27), 1.08 (3H, s, H-23), 1.08 (3H, s, H-24), 1.00 (3H, s, H-26), 0.88 (1H, s, H-25); ^13^C NMR (150 MHz, pyridine-*d*_5_) *δ*: 38.4 (C-1), 29.1 (C-2), 78.4 (C-3), 40.0 (C-4), 56.1 (C-5), 19.2 (C-6), 33.6 (C-7), 40.4 (C-8), 48.4 (C-9), 37.7 (C-10), 25.4 (C-11), 123.6 (C-12), 144.7 (C-13), 42.8 (C-14), 26.7 (C-15), 21.4 (C-16), 39.5 (C-17), 45.1 (C-18), 41.9 (C-19), 43.0 (C-20), 38.2 (C-21), 75.7 (C-22), 29.3 (C-23), 17.0 (C-24), 16.2 (C-25), 17.6 (C-26), 28.5 (C-27), 25.9 (C-28), 181.92 (C-29), 24.3 (C-30).

2*α*,3*β*,24-trihydroxy olea-12-en-28-oic acid (**12**): white amorphous powder; ^1^H NMR (500 MHz, DMSO-*d*_6_) *δ*: 5.15 (1H, brs, H-12), 4.24 (1H, m, H-2), 3.90 (1H, brs, H-3), 3.71 (1H, d, *J* = 10.7 Hz, H-24), 3.20 (1H, d, *J* = 10.7 Hz, H-24),1.09 (3H, s, H-27), 0.93 (3H, s, H-23), 0.87 (6H, s, H-25, 30), 0.85 (3H, s, H-29), 0.67 (3H, s, H-26); ^13^C NMR (125 MHz, DMSO-*d*_6_) *δ*: 41.3 (C-1), 64.7 (C-2), 72.6 (C-3), 41.8 (C-4), 48.3 (C-5), 18.0 (C-6), 32.8 (C-7), 47.1 (C-9), 37.7 (C-10), 23.2 (C-11), 121.5 (C-12), 143.9 (C-13), 43.8 (C-14), 27.2 (C-15), 22.9 (C-16), 45.7 (C-17), 40.8 (C-18), 45.5 (C-19), 30.4 (C-20), 33.4 (C-21), 32.2 (C-22), 22.6 (C-23), 63.8 (C-24), 16.8 (C-25), 16.5 (C-26), 25.7 (C-27), 178.6 (C-28), 32.9 (C-29), 23.4 (C-30).

Erythrodiol (**13**): white amorphous powder; ^1^H NMR (600 MHz, CDCl_3_) *δ*: 5.19 (1H, d, *J* = 3.3 Hz, H-12), 3.56 (1H, d, *J* = 11.0 Hz, H-28), 3.22 (1H, d, *J* = 11.0 Hz, H-28), 3.12 (1H, m, H-3), 1.16 (3H, s, H-27), 0.99 (3H, s, H-23), 0.94 (3H, s, H-24), 0.93 (3H, s, H-30), 0.88 (3H, s, H-29), 0.86 (3H, s, H-26), 0.78 (3H, s, H-25); ^13^C NMR (150 MHz, CDCl_3_) *δ*: 38.7 (C-1), 26.1 (C-2),79.1 (C-3), 38.9 (C-4), 55.3 (C-5), 18.5 (C-6), 32.7 (C-7), 39.9 (C-8), 47.7 (C-9), 37.0 (C-10), 22.1 (C-11), 122.5 (C-12), 144.3 (C-13), 41.8 (C-14), 28.2 (C-15), 23.7 (C-16), 37.0 (C-17), 42.4 (C-18), 46.6 (C-19), 31.1 (C-20), 34.2 (C-21), 31.2 (C-22), 27.3 (C-23), 15.7 (C-24), 15.6 (C-25), 16.8 (C-26), 25.7 (C-27), 69.8 (C-28), 33.3 (C-29), 23.6 (C-30).

Oleanolic acid (**14**): white amorphous powder; ^1^H NMR (400 MHz, CDCl_3_) *δ*: 5.27 (1H, d, *J* = 3.5 Hz, H-12), 3.12 (1H, m, H-3), 1.16 (3H, s, H-27), 0.99 (3H, s, H-23), 0.93 (3H, s, H-30), 0.90 (3H, s, H-29), 0.91 (3H, s, H-25), 0.77 (3H, s, H-24), 0.74 (3H, s, H-26); ^13^C NMR (100 MHz, CDCl_3_) *δ*: 38.5 (C-1), 27.3 (C-2), 79.1 (C-3), 38.9 (C-4), 55.3 (C-5), 18.5 (C-6), 32.7 (C-7), 39.4 (C-8), 47.7 (C-9), 37.0 (C-10), 23.1 (C-11), 122.8 (C-12), 143.7 (C-13), 41.8 (C-14), 27.8 (C-15), 23.7 (C-16), 46.7 (C-17), 41.1 (C-18), 46.0 (C-19), 30.8 (C-20), 33.9 (C-21), 32.6 (C-22), 28.2 (C-23), 15.7 (C-24), 15.6 (C-25), 17.3 (C-26), 26.1 (C-27), 183.6 (C-28), 33.3 (C-29), 23.6 (C-30).

Uvaol (**15**): white amorphous powder; ^1^H NMR (500 MHz, CDCl_3_) *δ*: 5.14 (1H, t, *J* = 3.6 Hz, H-12), 3.52 (1H, t, *J* = 8.2 Hz, H-28a), 2.89 (d, *J* = 15.5 Hz, 1H), 2.78 (1H, d, *J* = 15.5 Hz, H-28b), 1.25 (3H, s, H-27), 1.10 (s, 3H, H-25), 1.00 (s, 3H, H-23), 0.99 (s, 3H, H-26), 0.95 (s, 3H, H-29), 0.93 (s, 3H, H-30), 0.79 (s, 3H, H-24); ^13^C NMR (125 MHz, CDCl_3_) *δ*: 38.9 (C-1), 27.4 (C-2), 79.2 (C-3), 38.2 (C-4), 55.3 (C-5), 18.5 (C-6), 32.9 (C-7), 39.5 (C-8), 47.8 (C-9), 43.5 (C-10), 23.4 (C-11), 125.1 (C-12), 138.7 (C-13), 42.2 (C-14), 26.1 (C-15), 23.5 (C-16), 37.0 (C-17), 54.2 (C-18), 40.2 (C-19), 39.6 (C-20), 35.3 (C-21), 30.8 (C-22), 28.3 (C-23), 15.8 (C-24), 15.8 (C-25), 16.9 (C-26), 23.5 (C-27), 70.2 (C-28), 17.5 (C-29), 21.5 (C-30).

*β*-amyrin acetate (**16**): white amorphous powder; ^1^H NMR (500 MHz, CDCl_3_) *δ*: 4.50 (1H, m, H-3), 5.25 (1H, t, *J* = 3.6 Hz, H-12), 1.10 (3H, s, H-24), 0.90 (3H, s, H-25), 0.85 (3H, s, H-26), 0.89 (3H, s, H-27), 0.99 (3H, s, H-28), 1.05 (3H, s, H-29), 0.91 (3H, s, H-30), 0.93 (3H, s, H-31), 2.03 (3H, s, H-32). ^13^C NMR (125 MHz, CDCl_3_) *δ*: 38.4 (C-1), 23.7 (C-2), 81.1 (C-3), 37.8 (C-4), 55.4 (C-5), 18.4 (C-6), 32.7 (C-7), 39.8 (C-8), 47.6 (C-9), 37.0 (C-10), 23.6 (C-11), 121.8 (C-12), 145.4 (C-13), 41.9 (C-14), 26.3 (C-15), 27.1 (C-16), 32.6 (C-17), 47.4 (C-18), 46.9 (C-19), 31.2 (C-20), 34.8 (C-21), 37.3 (C-22), 16.9 (C-23), 28.2 (C-24), 15.7 (C-25), 16.9 (C-26), 26.1 (C-27), 23.8 (C-28), 33.4 (C-29), 28.5 (C-30), 171.2 (C=O), 21.4 (CH_3_-C=O).

2*α*-hydroxy ursolic acid (**17**): white amorphous powder; ^1^H NMR (500 MHz, DMSO-*d*_6_) *δ*: 5.16 (1H, dd, *J* = 15.2, 5.6 Hz, H-12), 1.08 (3H, s, H-27), 0.92 (9H, m, H-23, 24, 26), 0.88 (1H, s, H-25), 0.74 (3H, s, H-29), 0.70 (3H, s, H-30); ^13^C NMR (125 MHz, DMSO-*d*_6_) *δ*: 47.1 (C-1), 67.5 (C-2), 82.3 (C-3), 39.5 (C-4), 54.8 (C-5), 18.2 (C-6), 32.6 (C-7), 38.9 (C-8), 46.8 (C-9), 36.3 (C-10), 23.0 (C-11), 124.5 (C-12), 138.2 (C-13), 41.7 (C-14), 27.5 (C-15), 23.8 (C-16), 47.0 (C-17), 52.4 (C-18), 38.4 (C-19), 37.6 (C-20), 30.2 (C-21), 36.3 (C-22), 28.8 (C-23), 17.1 (C-24), 17.0 (C-25), 17.2 (C-26), 23.3 (C-27), 178.3 (C-28), 16.4 (C-29), 21.5 (C-30).

Betulinic acid (**18**): white amorphous powder; ^1^H NMR (500 MHz, CDCl_3_) *δ*: 4.78 (1H, brs, H-29a), 4.61 (1H, brs, H-29b), 3.19 (1H, dd, *J* = 11.2, 4.9 Hz, H-3), 1.69 (3H, s, H-30), 0.97 (3H, s, H-23), 0.96 (3H, s, H-26), 0.93 (3H, s, H-25), 0.82 (3H, s, H-24), 0.75 (3H, s, H-27); ^13^C NMR (125 MHz, CDCl_3_) *δ*: 38.7 (C-1), 27.4 (C-2), 79.0 (C-3), 38.9 (C-4), 55.6 (C-5), 18.3 (C-6), 34.3 (C-7), 40.7 (C-8), 50.5 (C-9), 37.2 (C-10), 20.8 (C-11), 25.5 (C-12), 38.4 (C-13), 42.4 (C-14), 29.5 (C-15), 32.1 (C-16), 56.3 (C-17), 46.9 (C-18), 49.2 (C-19), 150.5 (C-20), 29.7 (C-21), 37.0 (C-22), 28.0 (C-23), 15.4 (C-24), 16.0 (C-25), 16.1 (C-26), 14.7 (C-27), 77.0 (C-28), 109.7 (C-29), 19.4 (C-30).

Friedelin (**19**): white amorphous powder; ^1^H-NMR (500 MHz, CDCl_3_) *δ*: 0.72 (3H, s, H-3), 0.87 (3H, s), 0.90 (3H, s), 0.95 (3H, s), 0.98 (3H, s), 1.01 (3H, s), 1.05 (3H, s), 1.18 (3H, s); ^13^C NMR (125 MHz, CDCl_3_) *δ*: 22.2 (C-1), 41.7 (C-2), 213.1 (C-3), 58.2 (C-4), 42.2 (C-5), 41.4 (C-6), 18.2 (C-7), 53.2 (C-8), 37.6 (C-9), 59.5 (C-10), 36.1 (C-11), 30.5 (C-12), 39.8 (C-13), 38.4 (C-14), 35.2 (C-15), 32.6 (C-16), 30.0 (C-17), 42.9 (C-18), 35.3 (C-19), 28.1 (C-20), 32.9 (C-21), 39.4 (C-22), 7.0 (C-23), 14.7 (C-24) 18.0 (C-25), 20.2 (C-26), 18.6 (C-27), 32.2(C-28), 35.5 (C-29), 31.9 (C-30).

### 3.5. Enzymatic Hydrolysis of Compounds ***1***–***3***

Acidic hydrolysis of compounds **1***–***3** were carried out according to the method described previously [18]. The configuration of the sugar moiety was determined by comparing their retention times with the derivatives of authentic samples. D-glucose and L-arabinose were confirmed by comparison with those of authentic samples t_R_: 6.5 min (D-glucose); 6.8 min (L-arabinose) (Figures S49−S51).

### 3.6. Anti-Inflammatory Assay

All compounds showed no cytotoxicity on RAW 264.7 cells at the concentrations from 0 to 50. All isolates from the leaves of *H. littorali*s were subjected to MTT assays to assess the cell viability of LPS stimulated RAW 264.7 cell model. Anti-inflammatory activity of terpenoids were evaluated using LPS stimulated RAW 264.7 cell model. The productions of NO were determined by Griess analysis [41,42].

### 3.7. Molecular Docking Studies

Molecular docking simulations were performed using the software AutoDock Vina along with AutoDock Tools (ADT 1.5.6) using the hybrid Lamarckian Genetic Algorithm (LGA) [43]. The three-dimensional (3D) crystal structures of iNOS (PDB code, 3E6T) and COX-2 (PDB code, 1PXX) were obtained from the RCSB Protein Data Bank, which resolution was 2.5 Å [44]. The standard 3D structures (PDB format) of selected compounds for molecular docking were constructed by chem3D Pro 22.0 software, whose configurations were determined by their NOESY spectra and Chem3D modeling. The cubic grid box of 20 Å size (x, y, z) with a spacing of 1.000 Å and grid maps were built. All of the other parameters were used according to default settings of AutoDock Vina. Results differing by less than 2.0 Å in positional root mean-square deviation (RMSD) were clustered together, and the results of the most favorable free energy of binding were chosen as the resultant complex structures.

## 4. Conclusions

Six new compounds, heritieras C–H (**1**–**6**), and thirteen known triterpenoids (**7–19**) were isolated from the leaves of *H. littorali*s. Five triterpenoids decreased secretions of NO on LPS stimulated RAW 264.7 cells. Among these compounds, compound **18** substantially inhibit the release of NO. The results from molecular docking revealed the preliminary anti-inflammatory mechanism due to the bindings of iNOS/COX-2 with bioactive triterpenoids. Our research disclosed that *H. littorali*s is potentially useful as a medicine for the treatment of inflammation.

## Figures and Tables

**Table 1:** ^1^H NMR (600 MHz) and ^13^C NMR (150 MHz) spectral data of compounds **1**–**3** (pyridine-*d*_5_, *δ* in ppm, *J* in Hz).

	1		2		3	
NO.	*δ*_H_ (*J* in Hz)	*δ*_C_ (DEPT)	*δ*_H_ (*J* in Hz)	*δ*_C_ (DEPT)	*δ*_H_ (*J* in Hz)	*δ*_C_ (DEPT)
1	3.16, dt (10.3, 3.1); 2.08, m	36.1, CH_2_	3.20, dt (10.3, 3.1); 2.13, m	35.6, CH_2_	3.12, dt (10.3, 3.1); 2.16, m	36.0, CH_2_
2	2.07, m; 1.87, m	22.3, CH_2_	2.13, m; 1.86, m	22.0, CH_2_	2.08, m; 1.75, m	27.0, CH_2_
3	3.61, brs	81.8, CH	3.58, brs	81.5, CH	3.59, brs	75.7, CH
4		38.5, C		38.0, C		38.9, C
5	1.60, m	51.4, CH	1.64, m	50.9, CH	1.79, m	50.3, CH
6	1.57, m; 1.45, m	18.8, CH_2_	1.57, m; 1.45, m	18.3, CH_2_	1.54, m; 1.46, m	18.8, CH_2_
7	1.50, m; 1.16, m	36.8, CH_2_	1.52, m; 1.16, m	36.4, CH_2_	1.63, m; 1.21, m	36.8, CH_2_
8		41.7, C		41.5, C		41.8, C
9	1.91, m	54.4, CH	1.92, m	53.9, CH	2.05, m	54.7, CH
10		41.9, C		39.9, C		40.5, C
11	2.93, dt (8.5, 3.9); 1.53, m	35.0, CH_2_	2.93, dt (8.5, 3.9); 1.52, m	34.5, CH_2_	2.96, dt (12.2, 4.1); 1.54, m	34.8, CH_2_
12	4.47, m	78.0, CH	4.47, m	77.8, CH	4.54, m	77.2, CH
13	1.79, m	41.6, CH	1.79, m	41.2, CH	1.84, m	41.5, CH
14		50.5, C		50.0, C		50.5, C
15	1.37, m; 0.99, m	32.0, CH_2_	1.37, m; 0.99, m	31.5, CH_2_	1.44, m; 1.03, m	31.9, CH_2_
16	1.84, m; 1.76, m	26.7, CH_2_	1.86, m; 1.75, m	26.4, CH_2_	1.87, m; 1.64, m	26.8, CH_2_
17	1.88, m	50.0, CH	1.88, m	49.2, CH	1.90, m	49.7, CH
18	1.03, s	17.4, CH_3_	1.04, s	17.0, CH_3_	1.07, s	17.5, CH_3_
19	1.41, s	17.4, CH_3_	1.42, s	16.8, CH_3_	1.43, s	17.3, CH_3_
20		86.9, C		86.4, C		86.9, C
21	1.18, s	25.0, CH_3_	1.18, s	24.5, CH_3_	1.20, s	24.8, CH_3_
22	1.77, m; 1.57, m	34.6, CH_2_	1.75, m; 1.57, m	34.2, CH_2_	1.77, m; 1.60, m	34.7, CH_2_
23	2.05, m; 1.95, m	27.1, CH_2_	2.06, m; 1.95, m	26.7, CH_2_	2.05, m; 1.97, m	26.7, CH_2_
24	3.95, t (7.2)	84.7, CH	3.96, t (7.2)	84.2, CH	3.98, t (7.2)	84.6, CH
25		71.7, C		71.2, C		71.7, C
26	1.41, s	26.7, CH_3_	1.42, s	26.2, CH_3_	1.44, s	26.5, CH_3_
27	1.48, s	28.1, CH_3_	1.48, s	27.7, CH_3_	1.49, s	28.0, CH_3_
28	0.95, s	23.6, CH_3_	0.95, s	23.1, CH_3_	0.95, s	23.4, CH_3_
29	1.26, s	30.5, CH_3_	1.27, s	29.8, CH_3_	1.26, s	30.4, CH_3_
30	0.63, s	17.2, CH_3_	0.61, s	16.7, CH_3_	0.81, s	17.1, CH_3_
1′	4.72, d (6.4)	102.3, CH	4.73, d (6.5)	102.2, CH		
2′	4.43, dd (8.2, 6.4)	72.9, CH	4.31, m	75.1, CH		
3′	4.23, dd (8.2, 3.4)	75.2, CH	4.23, m	75.1, CH		
4′	4.37, m	69.6, CH	5.41, m	73.3, CH		
5′	4.33, m; 3.76, m	66.8, CH_2_	4.34, m; 3.53, m	63.5, CH_2_		
1″	5.14, d (8.0)	102.7, CH	5.14, d (8.0)	102.3, CH	5.17, d (7.8)	102.0, CH
2″	4.01, m	75.8, CH	4.02, m	75.3, CH	4.04, m	75.5, CH
3″	4.30, m	79.1, CH	4.29, m	78.7, CH	4.30, m	78.9, CH
4″	4.16, m	72.9, CH	4.15, m	72.3, CH	4.14, m	72.7, CH
5″	4.03, m	78.3, CH	4.04, m	77.8, CH	4.04, m	78.2, CH
6″	4.54, dd (11.3, 3.0); 4.37, dd (11.3, 5.5)	63.9, CH_2_	4.54, dd (11.3, 3.0); 4.37, dd (11.3, 5.5)	63.3, CH_2_	4.54, dd (11.3, 3.0); 4.37, dd (11.3, 5.5)	63.9, CH_2_
-OOCCH_3_				170.7, C		
-OOCCH_3_			1.98, s	20.9, CH_3_		

**Table 2:** ^1^H NMR and ^13^C NMR spectral data of compounds **4**–**6** (pyridine-*d*_5_, *δ* in ppm, *J* in Hz).

	4 ^a^		5 ^a^		6 ^b^	
NO.	*δ*_H_ (*J* in Hz)	*δ*_C_ (DEPT)	*δ*_H_ (*J* in Hz)	*δ*_C_ (DEPT)	*δ*_H_ (*J* in Hz)	*δ*_C_ (DEPT)
1	1.33, m; 1.78, m	40.8, CH_2_	1.00, m; 1.58, m	39.5, CH_2_	1.01, m; 1.60, m	39.5, CH_2_
2	2.37, m; 2.83, m	36.0, CH_2_	1.86, m; 1.86, m	28.5, CH_2_	1.86, m; 1.86, m	28.5, CH_2_
3		214.9, C	3.46, dd (11.2, 5.0)	78.4, CH	3.46, dd (11.2, 5.0)	78.4, CH
4		55.6, C		42.5, C		39.5, C
5	1.39, m	58.2, CH	0.88, m	56.1, CH	0.86, m	56.1, CH
6	1.18, m; 1.64, m	20.4, CH_2_	1.44, m; 1.63, m	19.2, CH_2_	1.45, m; 1.61, m	19.2, CH_2_
7	1.30, m; 1.49, m	33.3, CH_2_	1.35, m; 1.56, m	33.2, CH_2_	1.33, m; 1.55, m	33.2, CH_2_
8		40.6, C		39.8, C		39.8, C
9	1.68, m	47.9, CH	1.68, m	48.4, CH	1.69, m	48.4, CH
10		37.4, C		37.6, C		37.6, C
11	1.68, m; 1.91, m	24.6, CH_2_	1.90, m; 1.90, m	24.3, CH_2_	1.92, m; 1.94, m	24.3, CH_2_
12	5.35, t (3.6)	122.6, CH	5.36, t (3.4)	123.4, CH	5.41, t (3.6)	123.0, CH
13		145.0, C		144.8, C		145.1, C
14		42.4, C		41.1, C		42.5, C
15	0.97, m; 1.84, m	26.9, CH_2_	1.00, m; 1.90, m	26.9, CH_2_	1.01, m; 1.90, m	27.0, CH_2_
16	1.01, m; 1.96, m	27.8, CH_2_	1.10, m; 2.06, m	28.0, CH_2_	1.05, m; 2.14, m	27.8, CH_2_
17		39.6, C		39.8, C		41.5, C
18	2.61, dd (13.8, 4.3)	44.3, CH	2.89, dd (13.8, 4.3)	44.6, CH	2.75, dd (13.8, 4.3)	43.7, CH
19	1.34, m; 2.09, m	47.6, CH_2_	1.81, m; 2.06, m	43.9, CH_2_	1.40, m; 2.58, m	41.4, CH_2_
20		37.0, C		40.6, C		40.7, C
21	3.87, s	75.0, CH	4.15, d (3.4)	76.6, CH	4.51, d (3.5)	70.8, CH
22	3.78, t (3.0)	80.0, CH	3.84, s	79.8, CH	3.87, s	80.2, CH
23	1.52, s	21.2, CH_3_	1.26, s	29.1, CH_3_	1.25, s	29.1, CH_3_
24	3.89, d (11.1) 4.37, d (11.1)	65.6, CH_2_	1.08, s	16.9, CH_3_	1.08, s	17.0, CH_3_
25	1.17, s	16.1, CH_3_	0.96, s	16.1, CH_3_	0.98, s	16.1, CH_3_
26	1.06, s	17.4, CH_3_	1.31, s	17.4, CH_3_	1.08, s	17.5, CH_3_
27	1.22, s	27.0, CH_3_	1.06, s	27.1, CH_3_	1.33, s	27.1, CH_3_
28	1.29, s	22.7, CH_3_	1.34, s	22.8, CH_3_	1.35, s	22.7, CH_3_
29	1.46, s	21.7, CH_3_	1.44, s	27.4, CH_3_	3.66, d (10.1) 4.07, d (10.1)	72.0, CH_3_
30	1.25, s	31.9, CH_3_	4.33, d (10.3) 4.42, d (10.3)	67.4, CH_2_	1.54, s	17.9, CH_2_

^a^ **4** and **5** were recorded in 600 MHz; ^b^ **6** was recorded in 400 MHz.

**Table 3:** Anti-inflammatory effects of active compounds (IC_50_ < 50 μM) on the production of the NO in LPS-stimulated RAW 264.7 Cells ^a^.

Compounds	IC_50_ (μM)
**1**	47.12 ± 0.61
**12**	25.23 ± 0.76
**16**	45.31 ± 0.45
**17**	39.98 ± 0.42
**18**	18.13 ± 0.32
Dexamethasone ^b^	16.37 ± 0.82

^a^ All values are means of three independent experiments. Values present mean ± SD of triplicate experiments. ^b^ Dexamethasone as positive control.

## Data Availability

The data of the article can be obtained from the authors.

## Supplementary Materials

The following supporting information can be downloaded at: https://www.mdpi.com/article/10.3390/molecules30010131/s1, Experimental details, including figures and tables of this manuscript, are provided as Supplementary Material.
