# Supplementary material for: New Triterpenoids from the Leaves of Heritiera littoralis and Their Anti-Inflammatory Activity
Source: Molecules. 2024 Dec 31;30(1):131. doi: 10.3390/molecules30010131 (PMC11721666; doi:10.3390/molecules30010131)
Supplement: Supplementary file 1 [file molecules-30-00131-s001.zip › molecules-3406054-supplementary.pdf]

SUPPLEMENTARY MATERIAL

New Triterpenoids from the Leaves of *Heritiera littoralis* and Their Anti-Inflammatory Activity

Chenyang Ren [1], Xiaoqin Liang[1], Rui Pi[2], Jiwen Xin [1], Bo Yang [1], Qingfang Zheng [1], Yanqin Li [1], Jun Li [1*], Yanjun Zhang [2*]

1 State Key Laboratory for Chemistry and Molecular Engineering of Medicinal Resources, Key Laboratory for Chemistry and Molecular Engineering of Medicinal Resources (Ministry of Education of China), Collaborative Innovation Center for Guangxi Ethnic Medicine, School of Chemistry and Pharmaceutical Sciences, Guangxi Normal University, Guilin 541004, China

2 Guangxi Key Laboratory of Green Chemical Materials and Safety Technology, College of Petroleum and Chemical Engineering, Beibu Gulf University, (Qinzhou 535000)

* Correspondence: lijun9593@gxnu.edu.cn (J. Li.); zhangyj201608@163.com (Y.J. Zhang.).

Abstract: Six new triterpenoids, heritieras C–H (**1**–**6**), along with thirteen known triterpenoids (**7**–**19**), were isolated from the leaves of *Heritiera littoralis*. Their structures were identified by spectroscopic analysis, including 1D and 2D nuclear magnetic resonance (NMR), high-resolution electrospray ionization mass spectrometry (HRESIMS), and by comparison with the literature. Anti-inflammatory activity of the isolates was evaluated using the lipopolysaccharide (LPS) stimulated RAW 264.7 cell model. Among the isolated triterpenoid, compounds **1**, **12**, **16**, **17**, and **18** demonstrated inhibitory activity against nitric oxide (NO) release, in which compound **18** exhibited the best activity with an IC_{50} value of 18.13 μM. The potential anti-inflammatory mechanism was investigated using molecular docking. The triterpenoids from *H. littoralis* could be served as potential candidates for the development of new anti-inflammatory agents.

Keywords: *Heritiera littoralis* Dryand.; heritiera C; heritiera D; anti-inflammatory

List of supporting information

Figure S1. HRESIMS spectrum of compound **1** 1
Figure S2. ^{1}H NMR (600 MHz, pyridine-d_5) spectrum of compound **1** 1
Figure S3. ^{13}C NMR (150 MHz, pyridine-d_5) spectrum of compound **1** 2
Figure S4. DEPT (150 MHz, pyridine-d_5) spectrum of compound **1** 2
Figure S5. ^{1}H-^{1}H COSY (600 MHz, pyridine-d_5) spectrum of compound 1 3
Figure S6. HSQC spectrum of compound **1** 3
Figure S7. HMBC spectrum of compound **1** 4
Figure S8. ROESY spectrum of compound **1** 4
Figure S9. HRESIMS spectrum of compound **2** 5
Figure S10. ^{1}H NMR (600 MHz, pyridine-d_5) spectrum of compound **2** 5
Figure S11. ^{13}C NMR (150 MHz, pyridine-d_5) spectrum of compound **2** 6
Figure S12. DEPT (150 MHz, pyridine-d_5) spectrum of compound **2** 6
Figure S13. ^{1}H-^{1}H COSY (600 MHz, pyridine-d_5) spectrum of compound **2** 7
Figure S14. HSQC spectrum of compound **2** 7
Figure S15. HMBC spectrum of compound **2** 8
Figure S16. ROESY spectrum of compound **2** 8
Figure S17. HRESIMS spectrum of compound **3** 9
Figure S18. ^{1}H NMR (600 MHz, pyridine-d_5) spectrum of compound **3** 9
Figure S19. ^{13}C NMR (150 MHz, pyridine-d_5) spectrum of compound **3** 10
Figure S20. DEPT (150 MHz, pyridine-d_5) spectrum of compound **3** 10
Figure S21. ^{1}H-^{1}H COSY (600 MHz, pyridine-d_5) spectrum of compound **3** 11
Figure S22. HSQC spectrum of compound **3** 11
Figure S23. HMBC spectrum of compound **3** 12
Figure S24. ROESY spectrum of compound **3** 12
Figure S25. HRESIMS spectrum of compound **4** 13
Figure S26. ^{1}H NMR (600 MHz, pyridine-d_5) spectrum of compound **4** 13
Figure S27. ^{13}C NMR (150 MHz, pyridine-d_5) spectrum of compound **4** 14
Figure S28. DEPT (150 MHz, pyridine-d_5) spectrum of compound **4** 14
Figure S29. ^{1}H-^{1}H COSY (600 MHz, pyridine-d_5) spectrum of compound **4** 15
Figure S30. HSQC spectrum of compound **4** 15
Figure S31. HMBC spectrum of compound **4** 16
Figure S32. NOESY spectrum of compound **4** 16
Figure S33. HRESIMS spectrum of compound **5** 17
Figure S34. ^{1}H NMR (600 MHz, pyridine-d_5) spectrum of compound **5** 17
Figure S35. ^{13}C NMR (150 MHz, pyridine-d_5) spectrum of compound **5** 18
Figure S36. DEPT (150 MHz, pyridine-d_5) spectrum of compound **5** 18
Figure S37. ^{1}H-^{1}H COSY (600 MHz, pyridine-d_5) spectrum of compound **5** 19
Figure S38. HSQC spectrum of compound **5** 19
Figure S39. HMBC spectrum of compound **5** 20
Figure S40. NOESY spectrum of compound **5** 20
Figure S41. HRESIMS spectrum of compound **6** 21
Figure S42. ^{1}H NMR (600 MHz, pyridine-d_5) spectrum of compound **6** 21

Figure S43. ^{13}C NMR (150 MHz, pyridine-d_5) spectrum of compound **6**......22
Figure S44. DEPT (150 MHz, pyridine-d_5) spectrum of compound **6**......22
Figure S45. ^1H-^1H COSY (600 MHz, pyridine-d_5) spectrum of compound **6**......23
Figure S46. HSQC spectrum of compound **6**......23
Figure S47. HMBC spectrum of compound **6**......24
Figure S48. NOESY spectrum of compound **6**......24
Figure S49. The HPLC plot of the standard and compound **3** of D-glucopyranose......25
Figure S50. The HPLC plot of the standard of L-arabinose......25
Figure S51. The HPLC plot of the compound **1** and **2** of D-glucopyranose and L-arabinose......25
Figure S52. The HPLC plots of the initial extract of *H. littorali*s and pure compound **2**......26
Figure S53. Molecular docking result of compound **1** with iNOS protein......27
Figure S54. Molecular docking result of compound **1** with COX-2 protein......27
Figure S55. Molecular docking result of compound **12** with iNOS protein......27
Figure S56. Molecular docking result of compound **12** with COX-2 protein......28
Figure S57. Molecular docking result of compound **16** with iNOS protein......28
Figure S58. Molecular docking result of compound **16** with COX-2 protein......28
Figure S59. Molecular docking result of compound **17** with iNOS protein......29
Figure S60. Molecular docking result of compound **17** with COX-2 protein......29
Figure S61. Molecular docking result of dexamethasone with iNOS protein......29
Figure S62. Molecular docking result of dexamethasone with COX-2 protein......30
Table S1. Logarithms of free binding energies (FBE, kcal/mol) of compounds **1**, **12**, **16**, **17**, **18,** and Dexamethasone to the active cavities of iNOS (PDB code: 3E6T) and targeting residues of the binding site located on the mobile flap......30
Table S2. Logarithms of free binding energies (FBE, kcal/mol) of compounds **1**, **12**, **16**, **17**, **18**, and Dexamethasone to the active cavities of COX-2 (PDB code: 1PXX) and targeting residues of the binding site located on the mobile flap......30

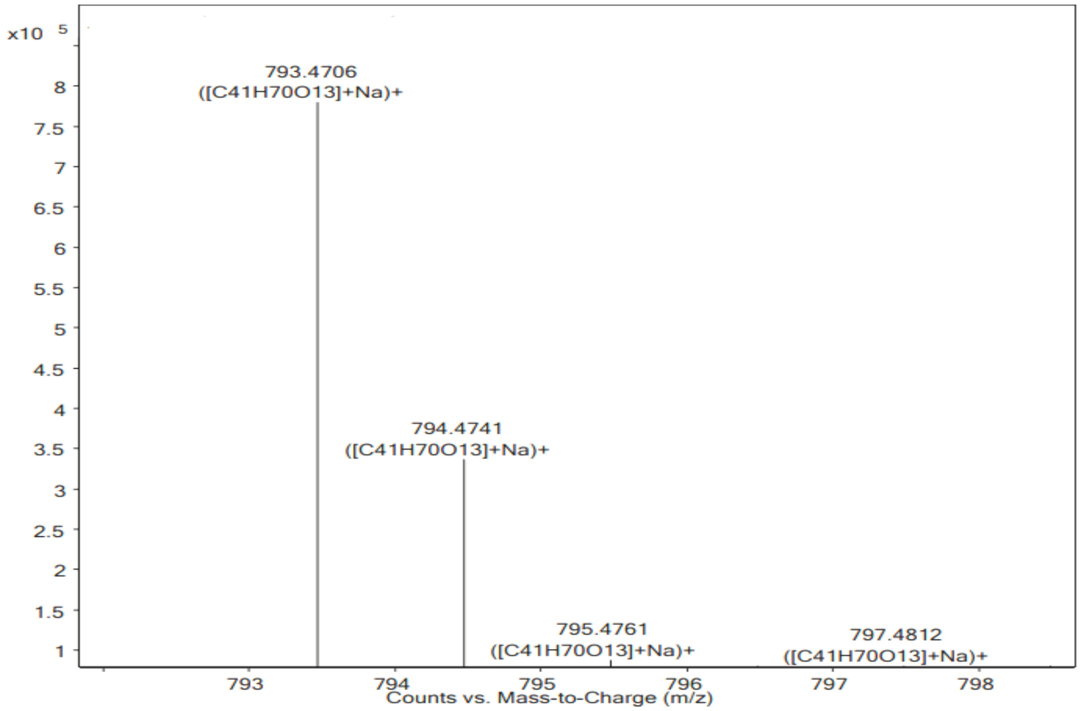

Figure S1. HRESIMS spectrum of compound **1**

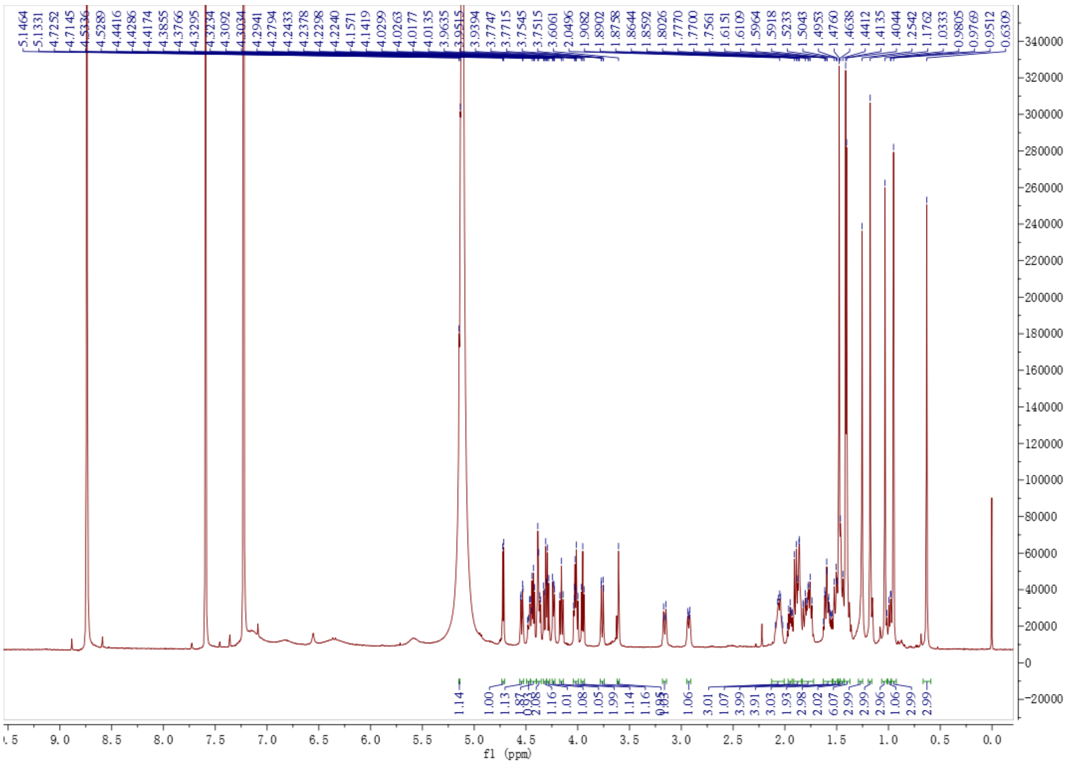

Figure S2. ^{1}H NMR (600 MHz, pyridine-d_5) spectrum of compound **1**

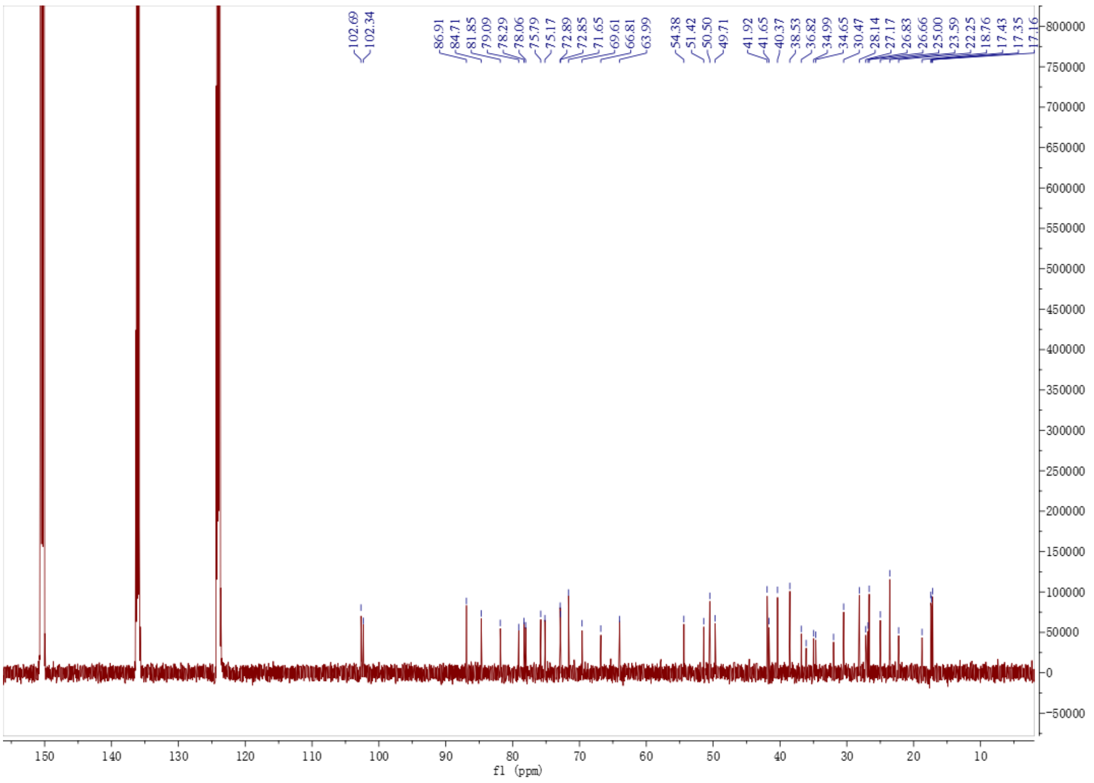

Figure S3. ^{13}C NMR (150 MHz, pyridine-d_5) spectrum of compound **1**

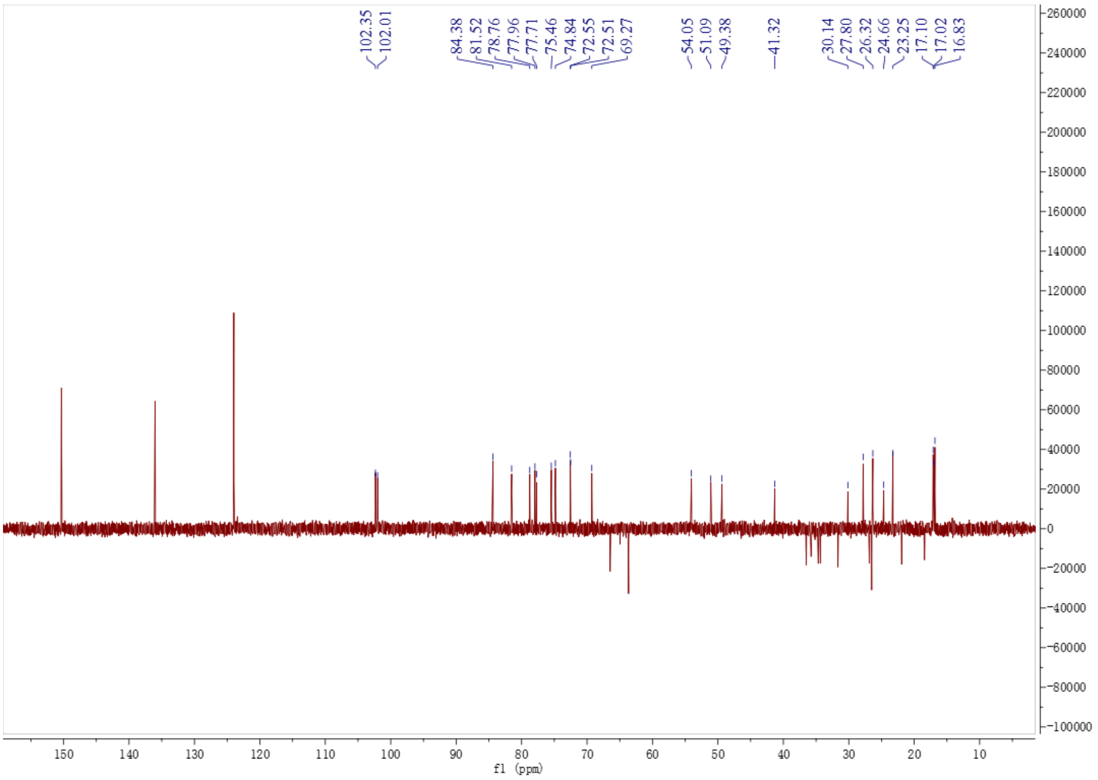

Figure S4. DEPT (150 MHz, pyridine-d_5) spectrum of compound **1**

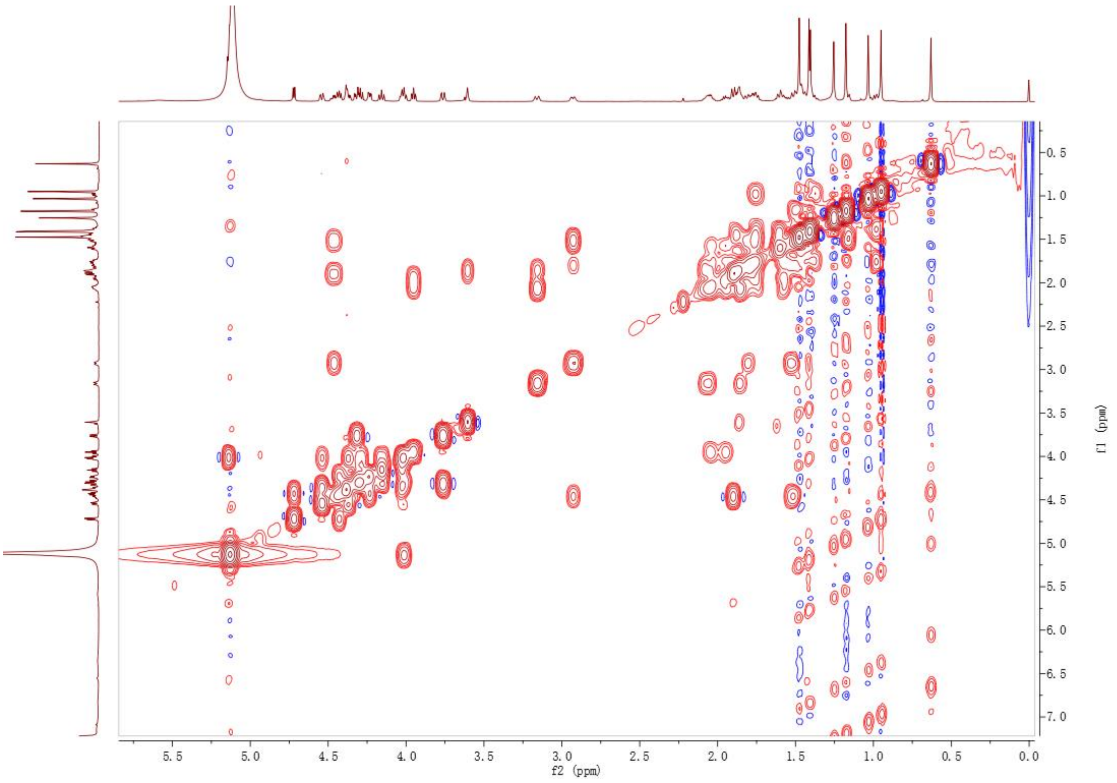

Figure S5. ^{1}H-^{1}H COSY (600 MHz, pyridine-d_5) spectrum of compound **1**

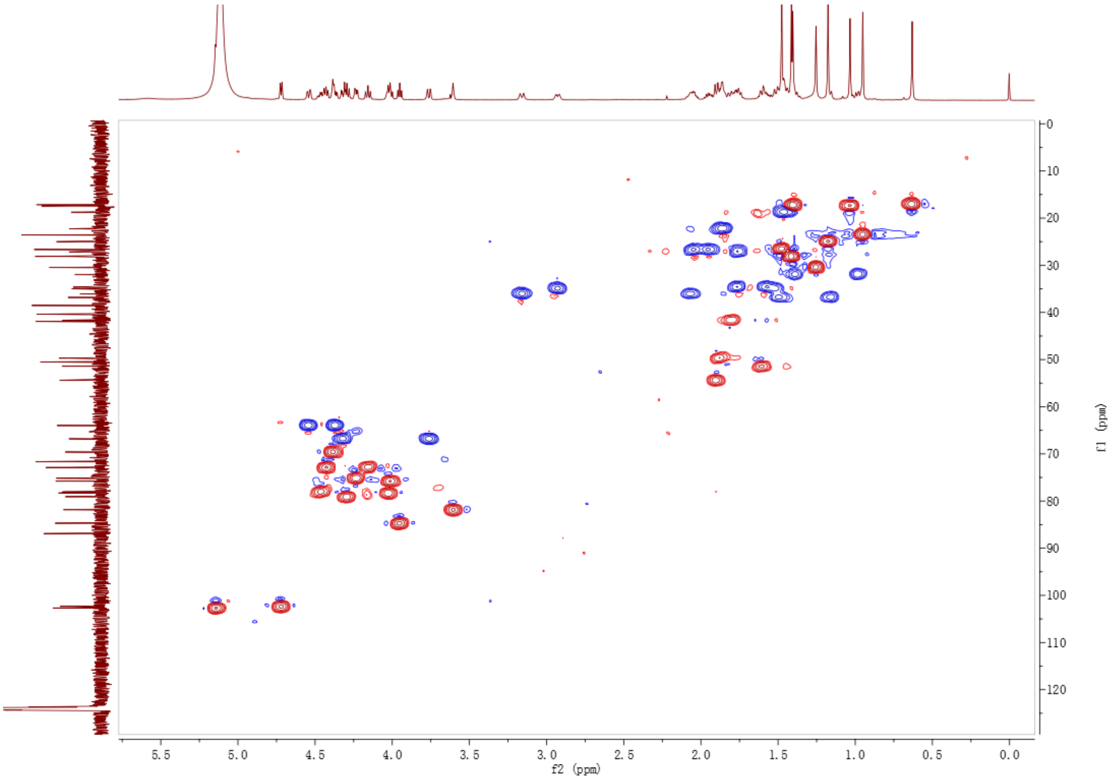

Figure S6. HSQC spectrum of compound **1**

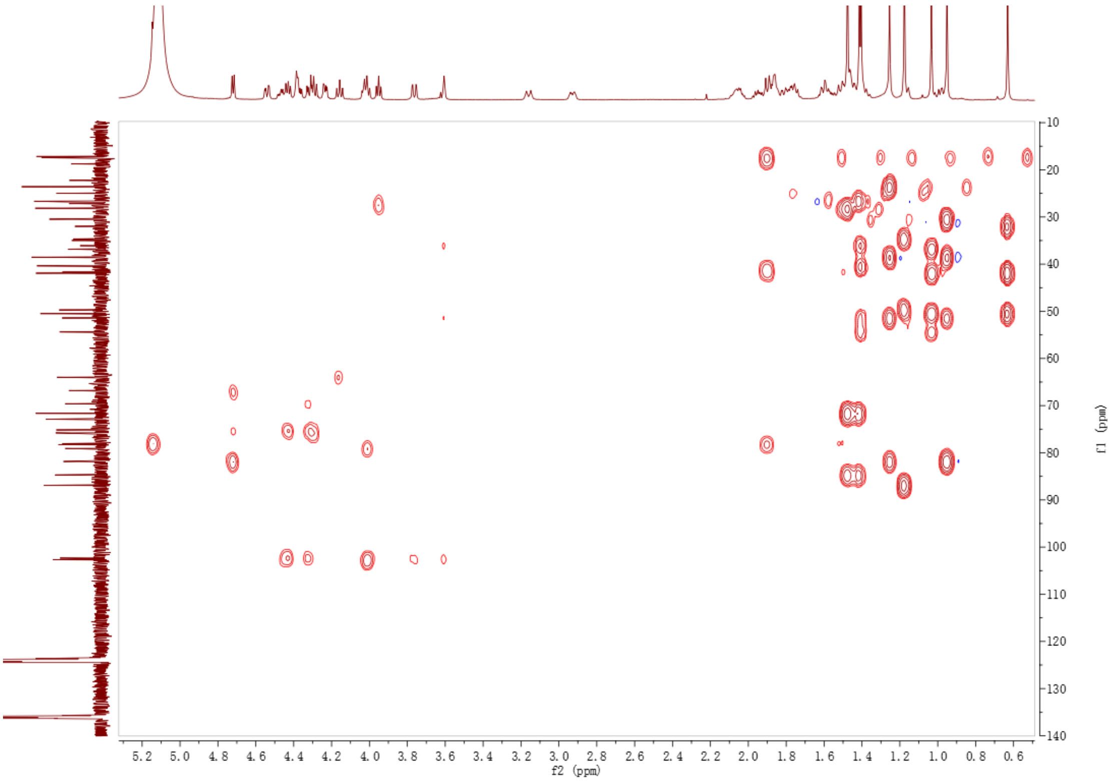

Figure S7. HMBC spectrum of compound **1**

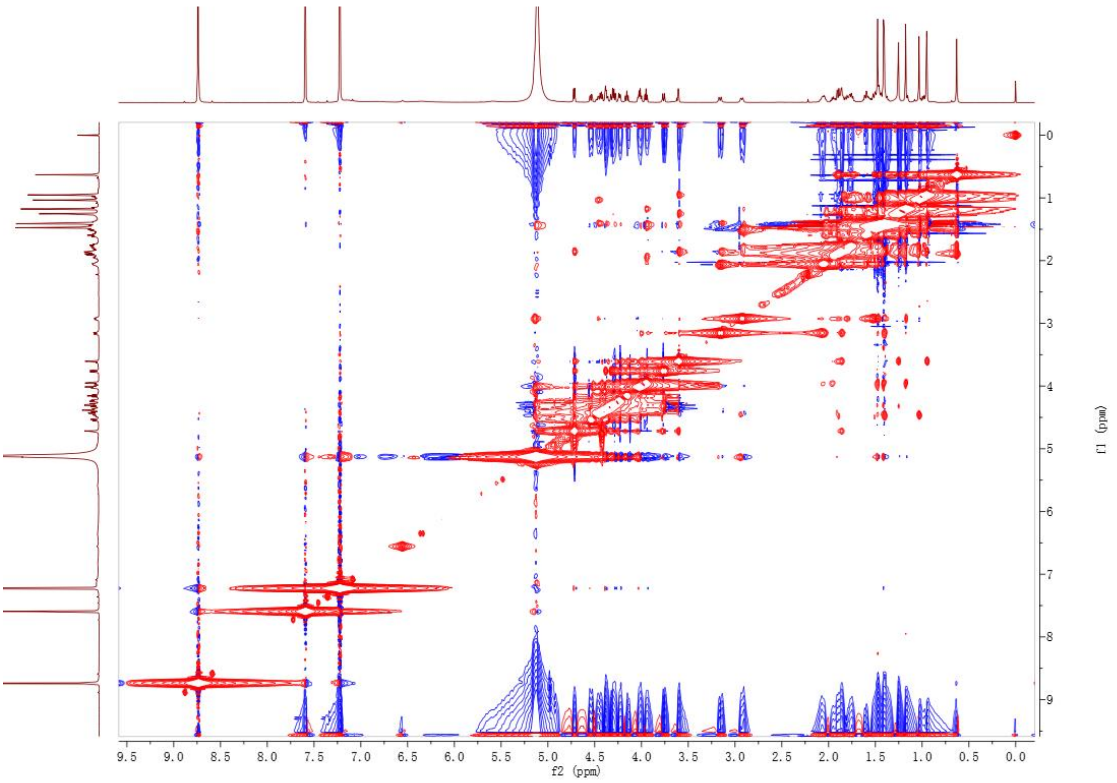

Figure S8. ROESY spectrum of compound **1**

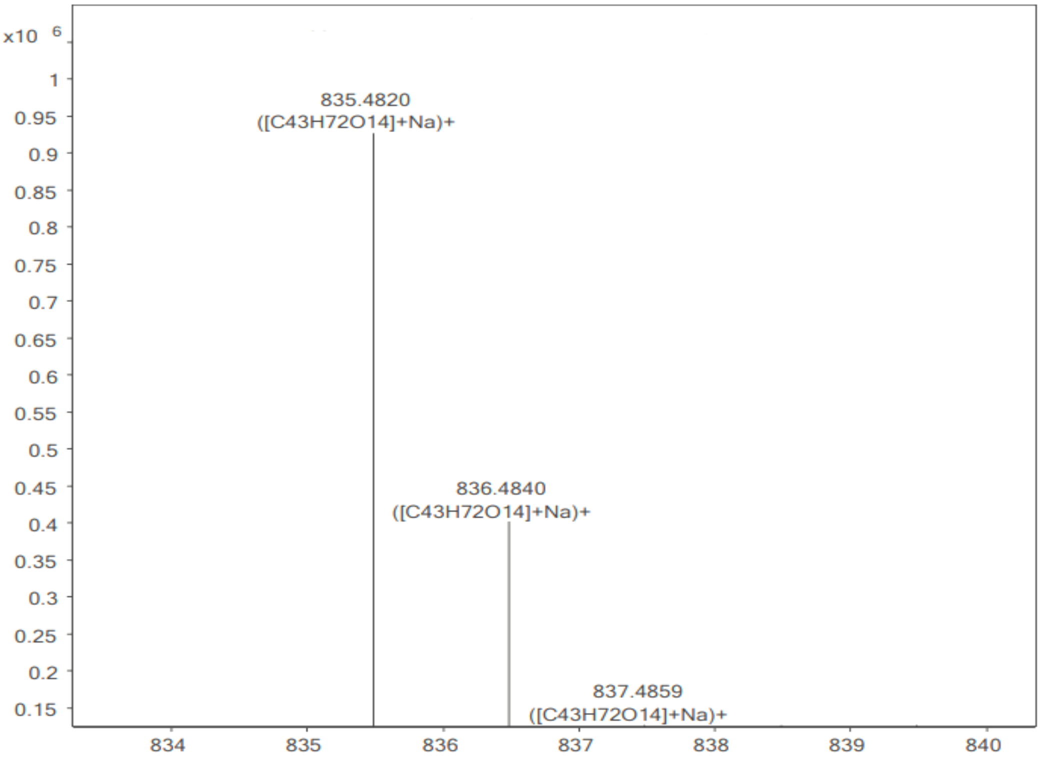

Figure S9. HRESIMS spectrum of compound **2**

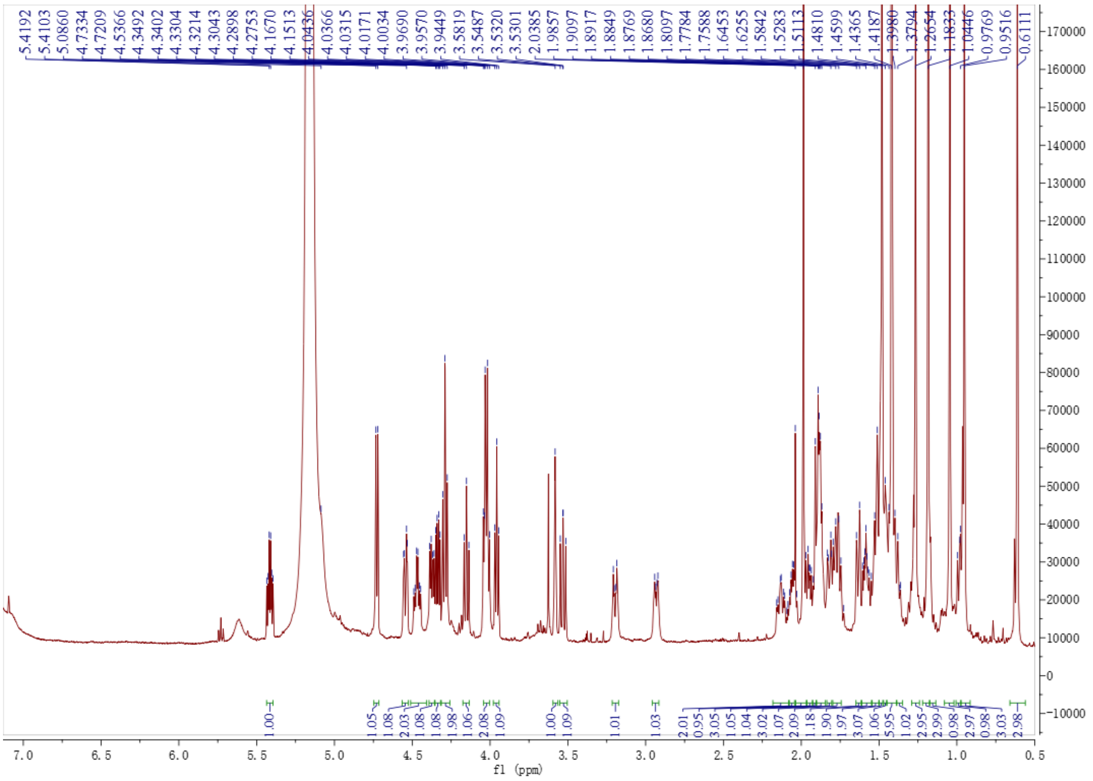

Figure S10. ^{1}H NMR (600 MHz, pyridine-d_5) spectrum of compound **2**

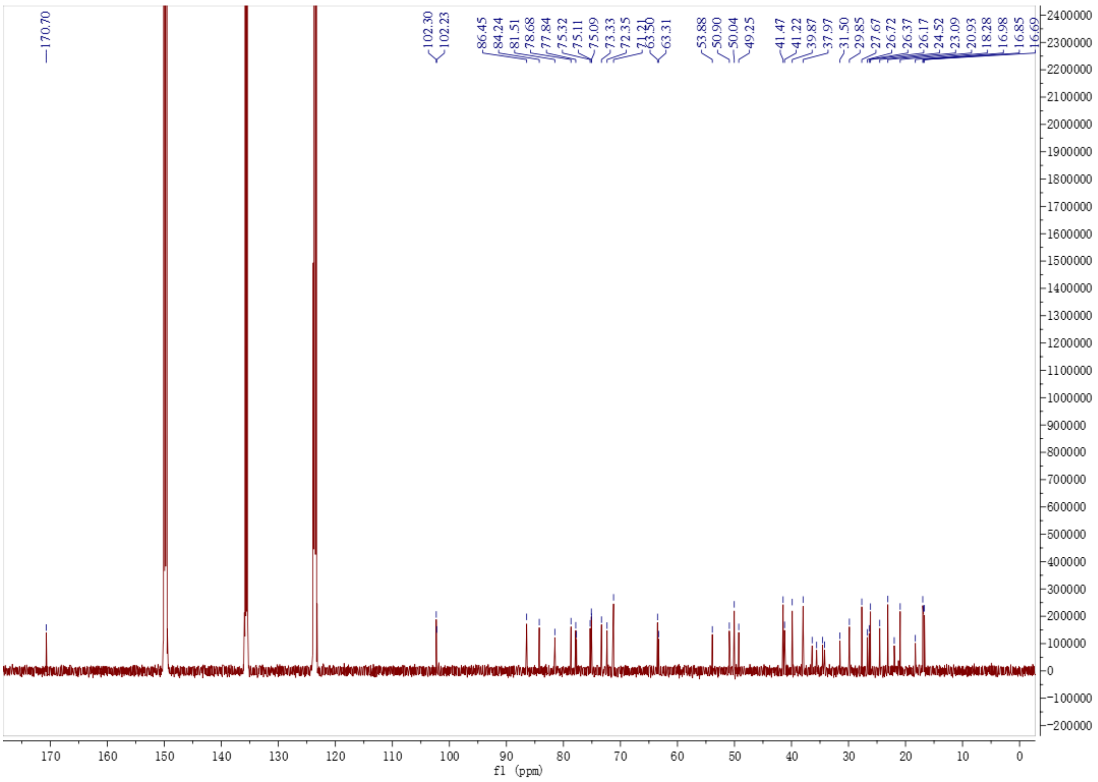

Figure S11. ^{13}C NMR (150 MHz, pyridine-d_5) spectrum of compound **2**

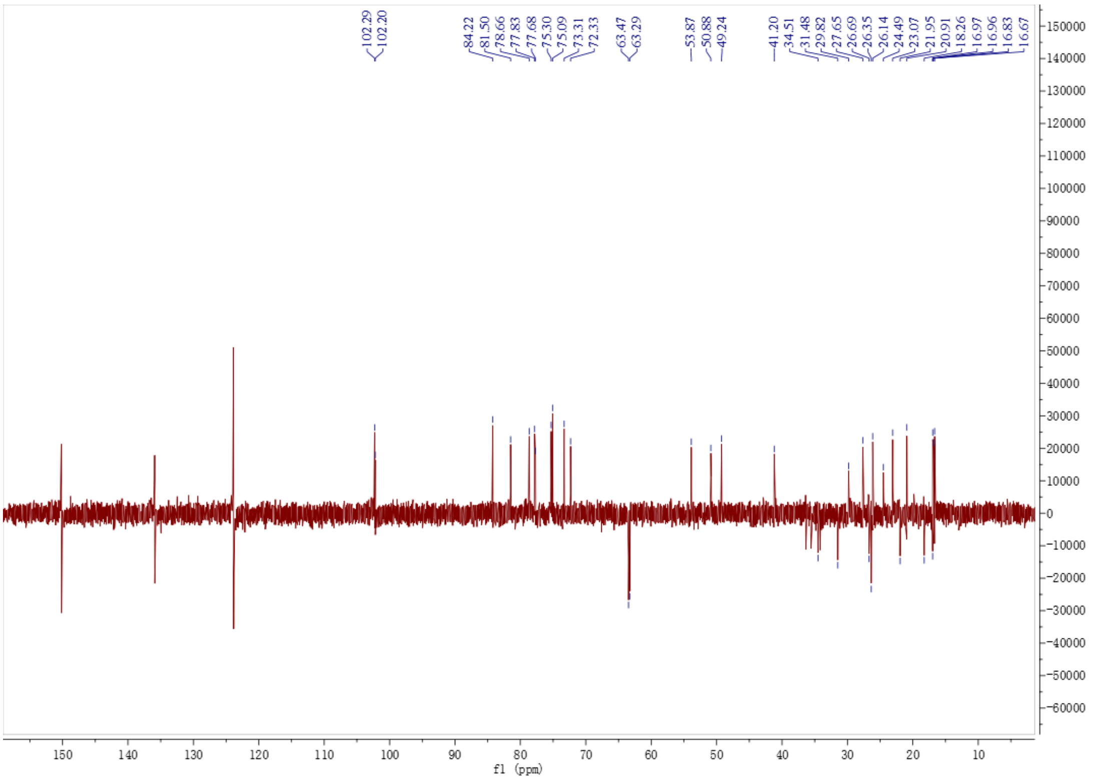

Figure S12. DEPT (150 MHz, pyridine-d_5) spectrum of compound **2**

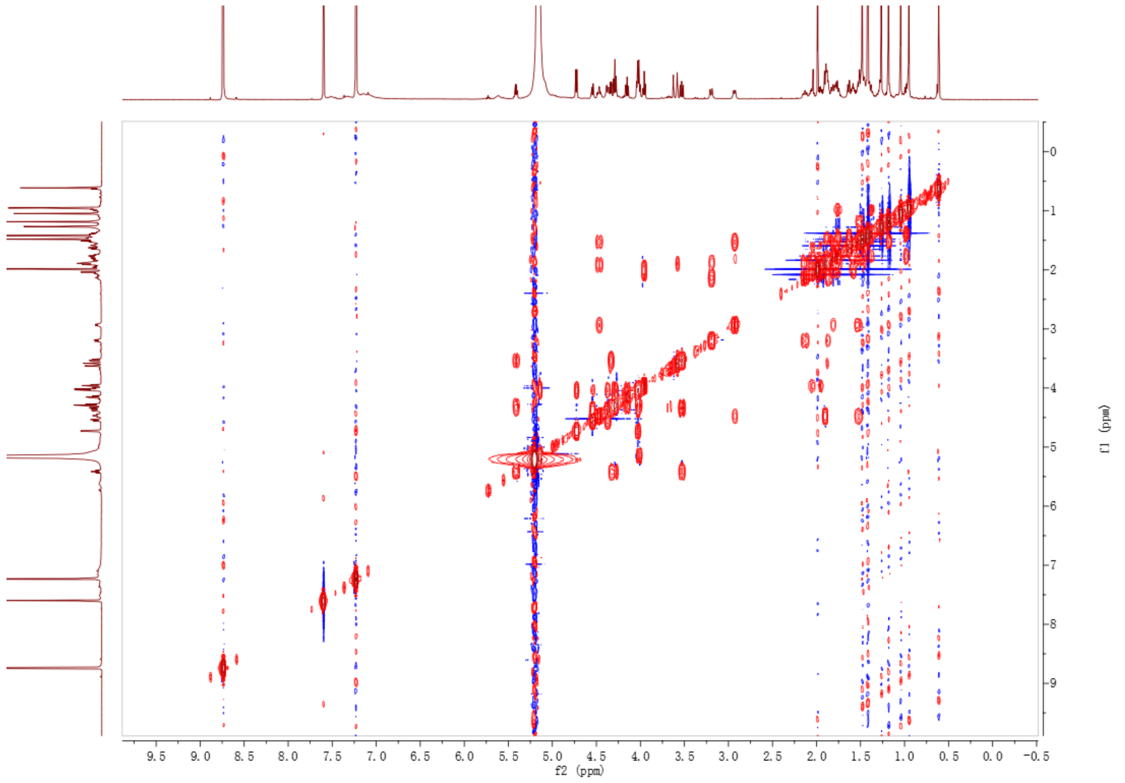

Figure S13. ^1H-^1H COSY (600 MHz, pyridine-d_5) spectrum of compound **2**

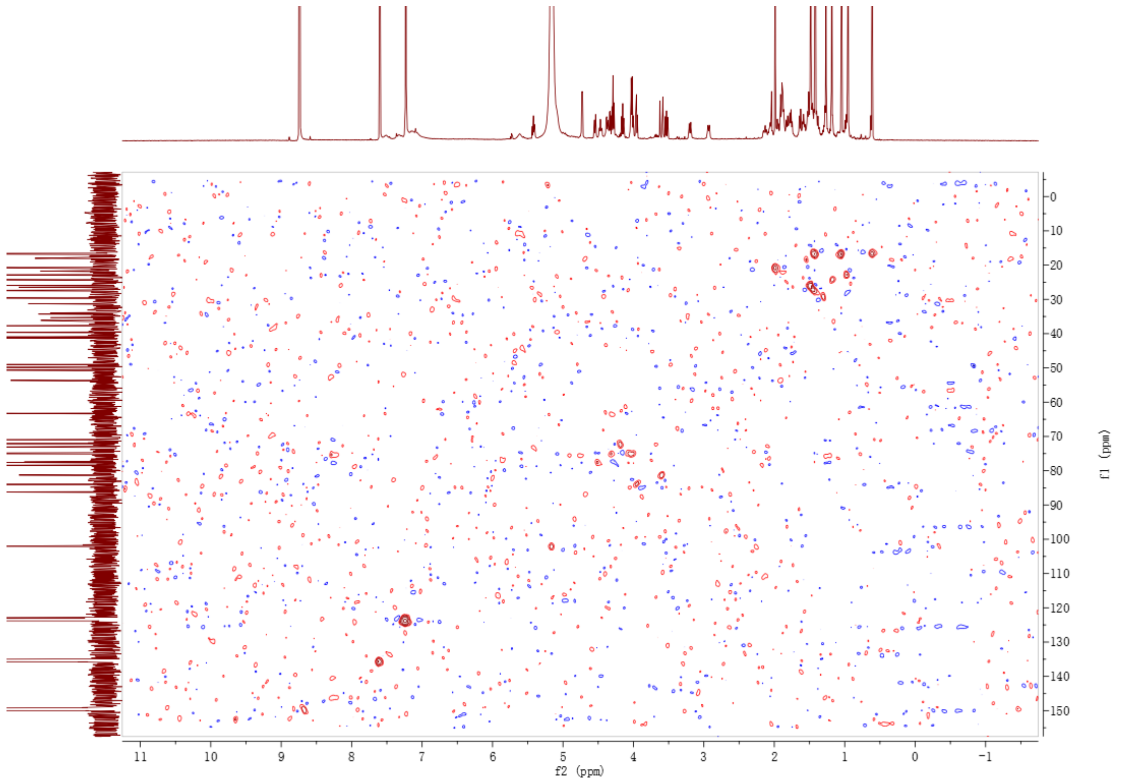

Figure S14. HSQC spectrum of compound **2**

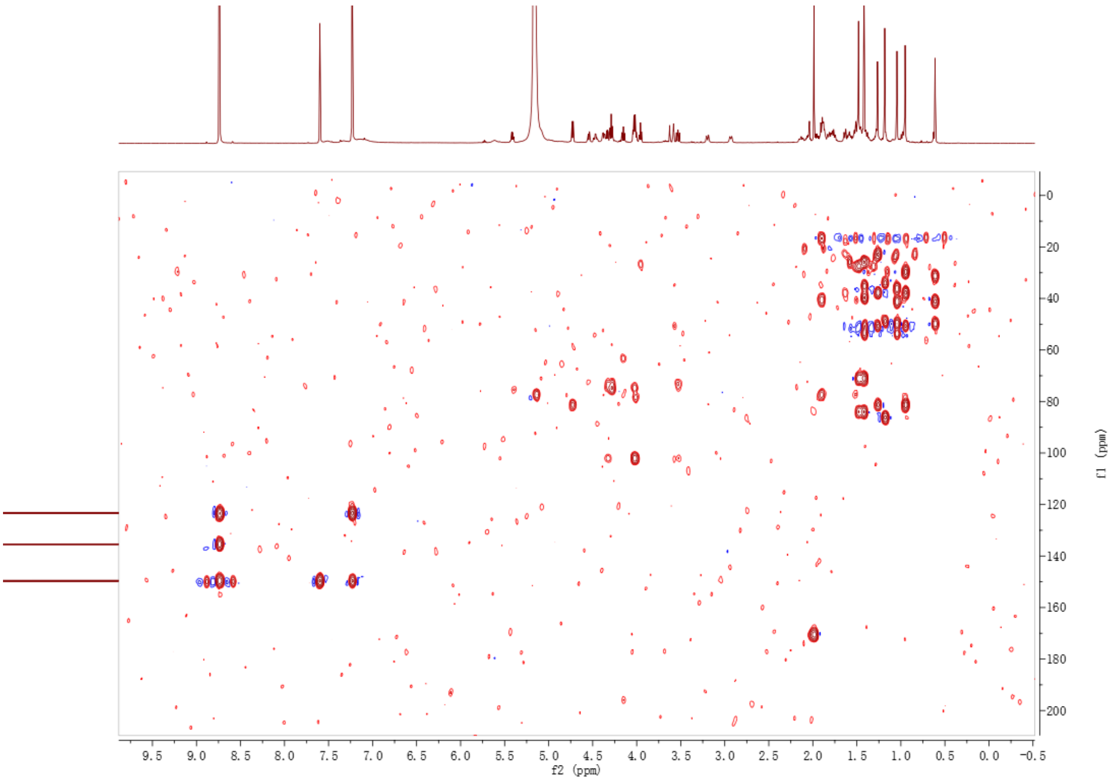

Figure S15. HMBC spectrum of compound **2**

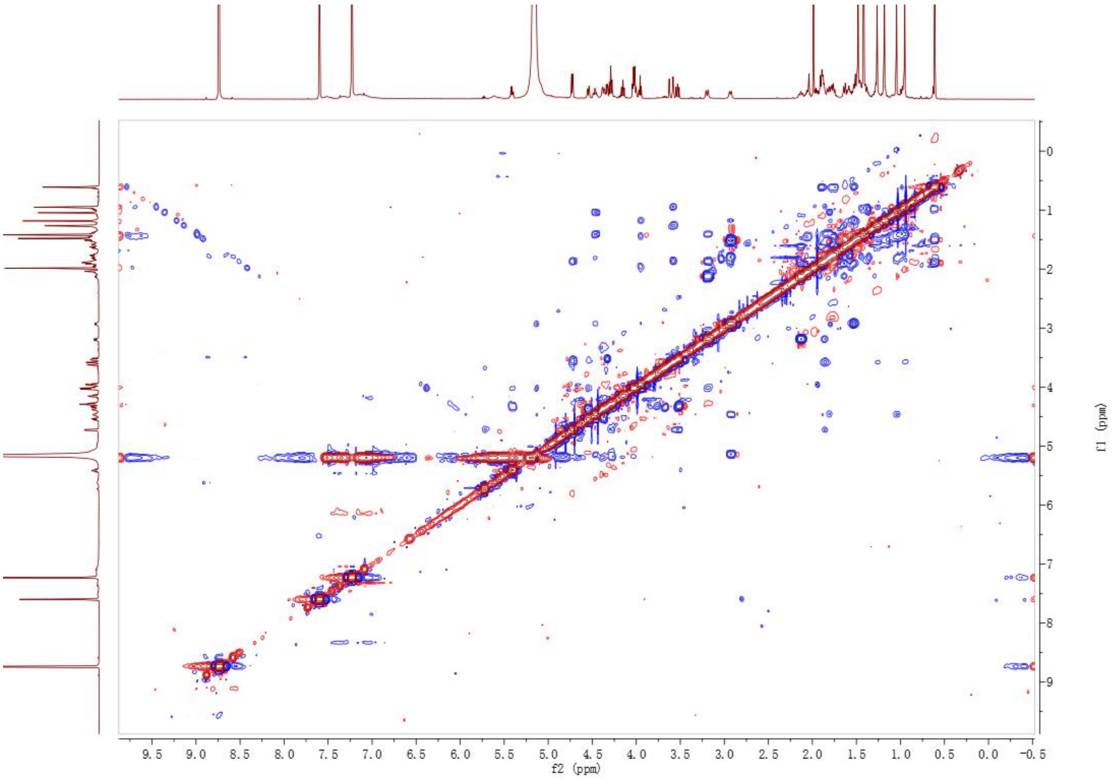

Figure S16. ROESY spectrum of compound **2**

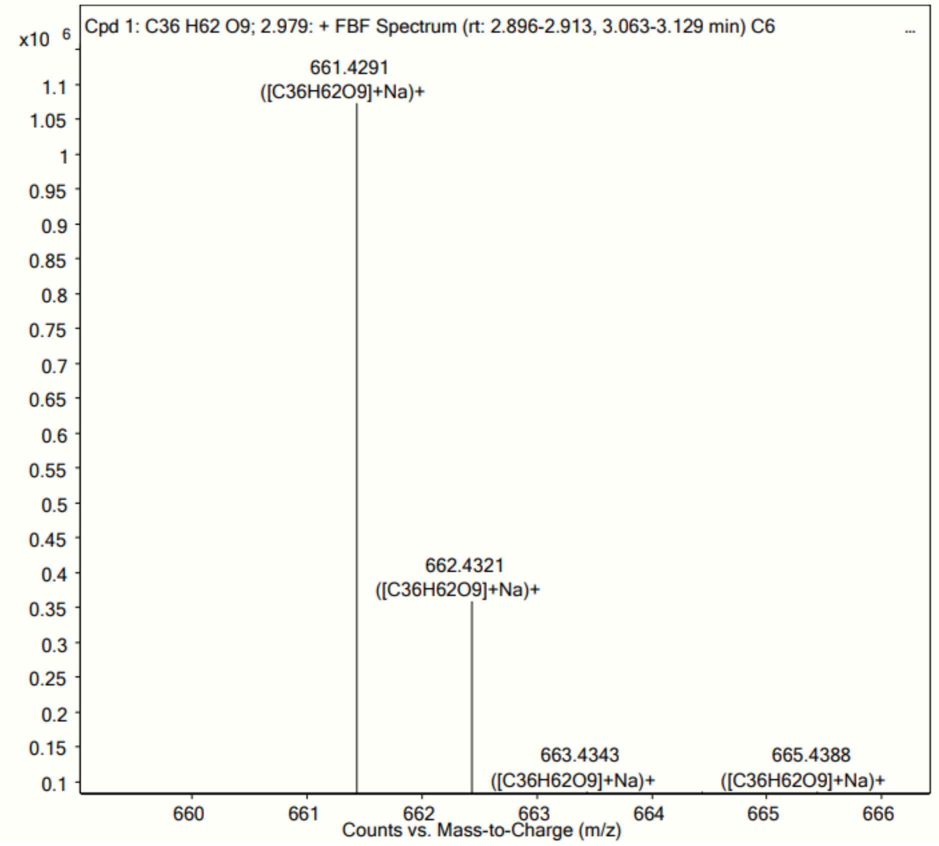

Figure S17. HRESIMS spectrum of compound **3**

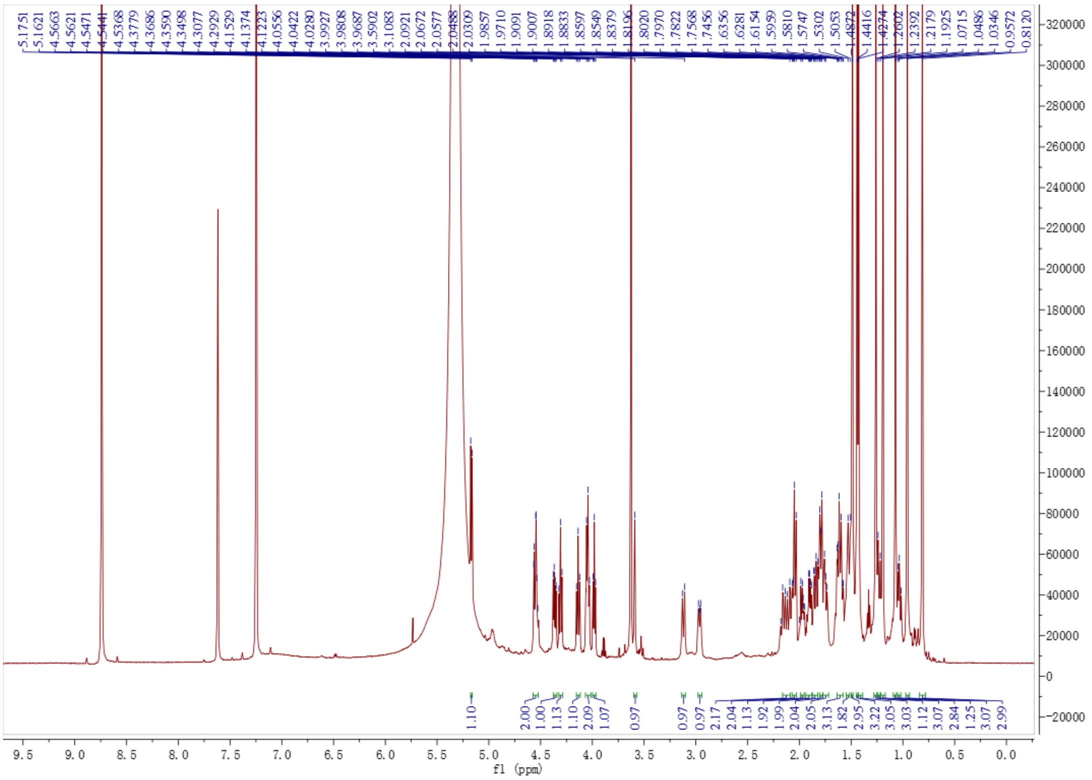

Figure S18. ^{1}H NMR (600 MHz, pyridine-d_5) spectrum of compound **3**

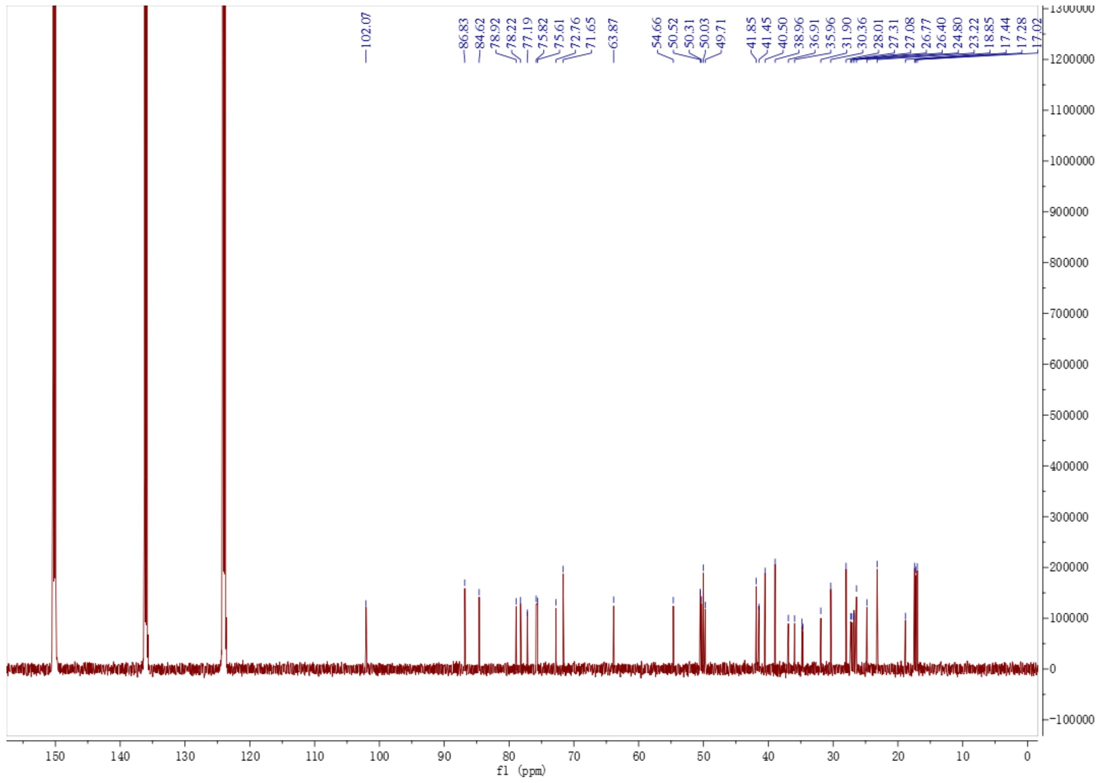

Figure S19. ^{13}C NMR (150 MHz, pyridine-d_5) spectrum of compound **3**

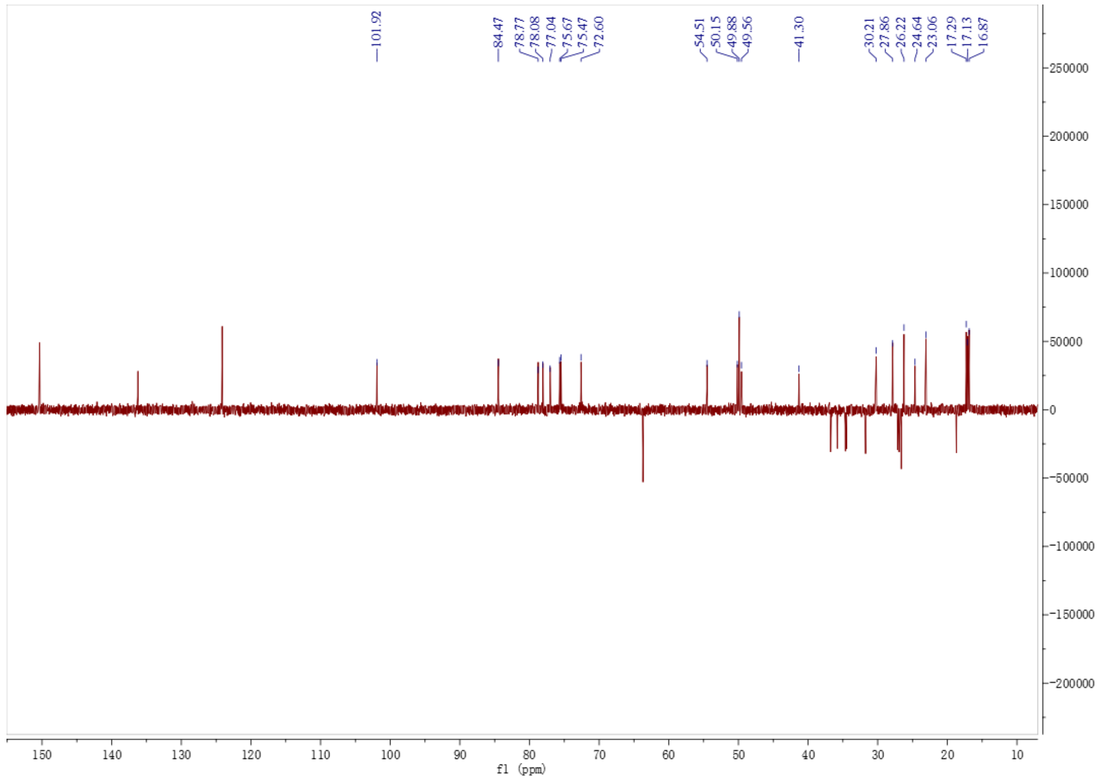

Figure S20. DEPT (150 MHz, pyridine-d_5) spectrum of compound **3**

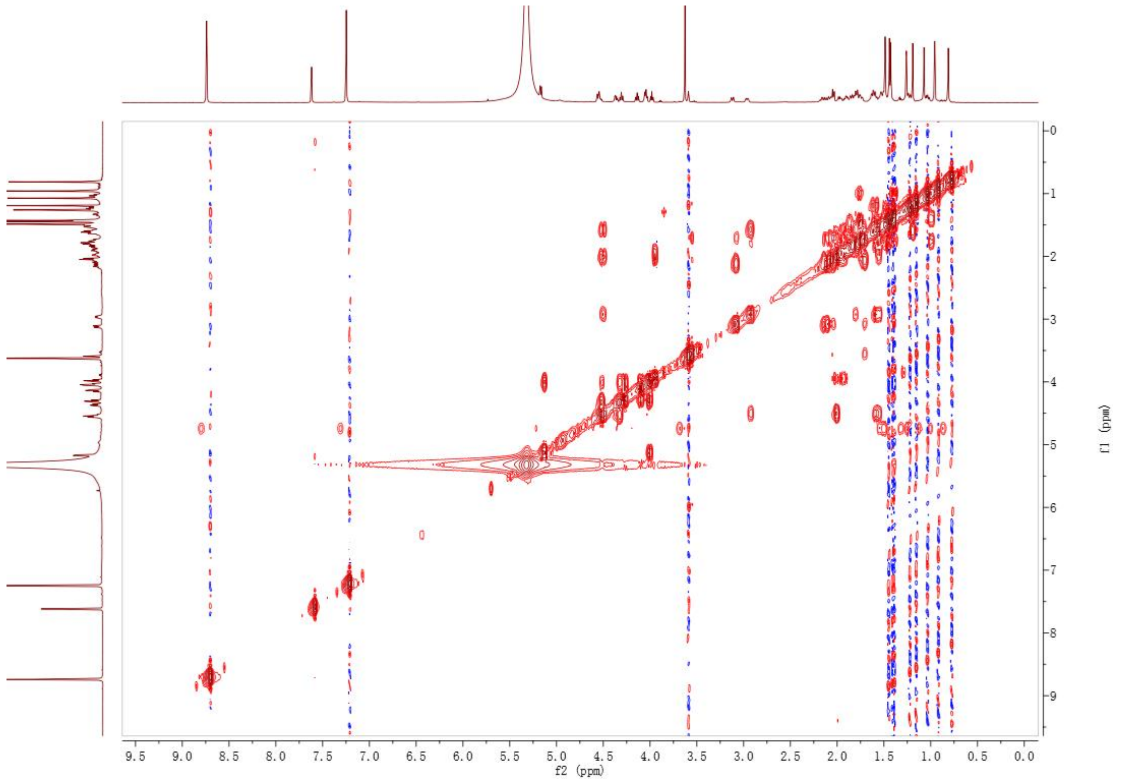

Figure S21. ^{1}H-^{1}H COSY (600 MHz, pyridine-d_5) spectrum of compound **3**

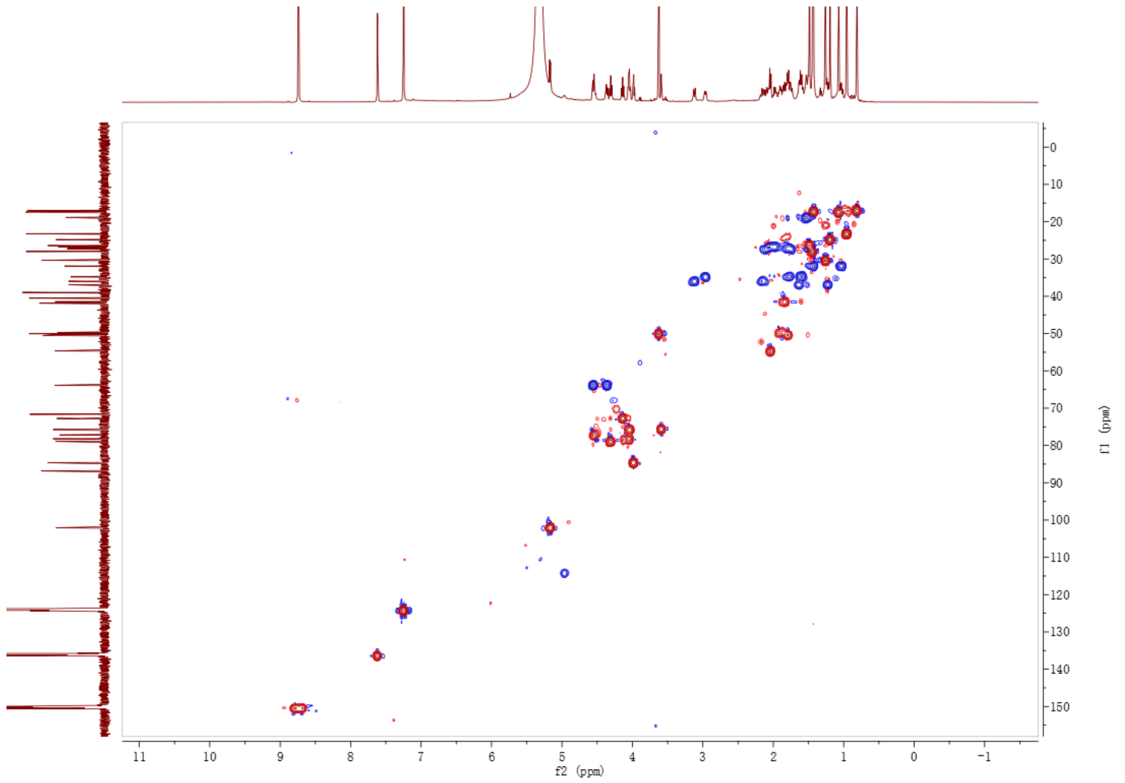

Figure S22. HSQC spectrum of compound **3**

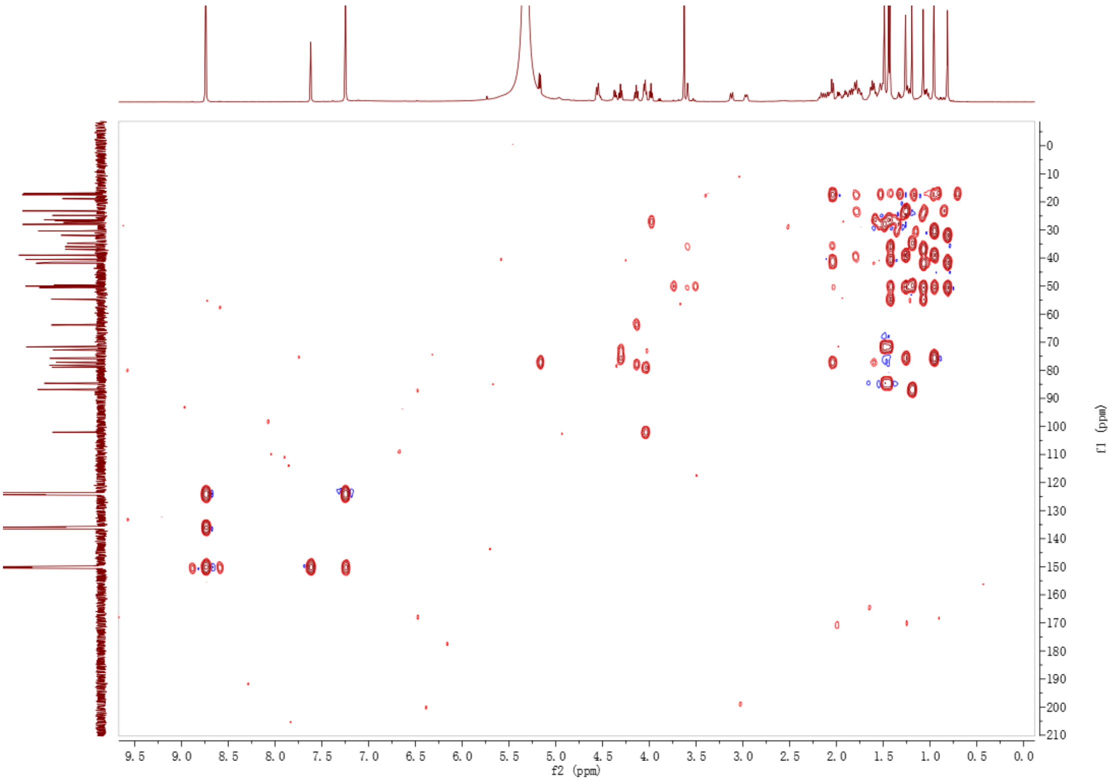

Figure S23. HMBC spectrum of compound **3**

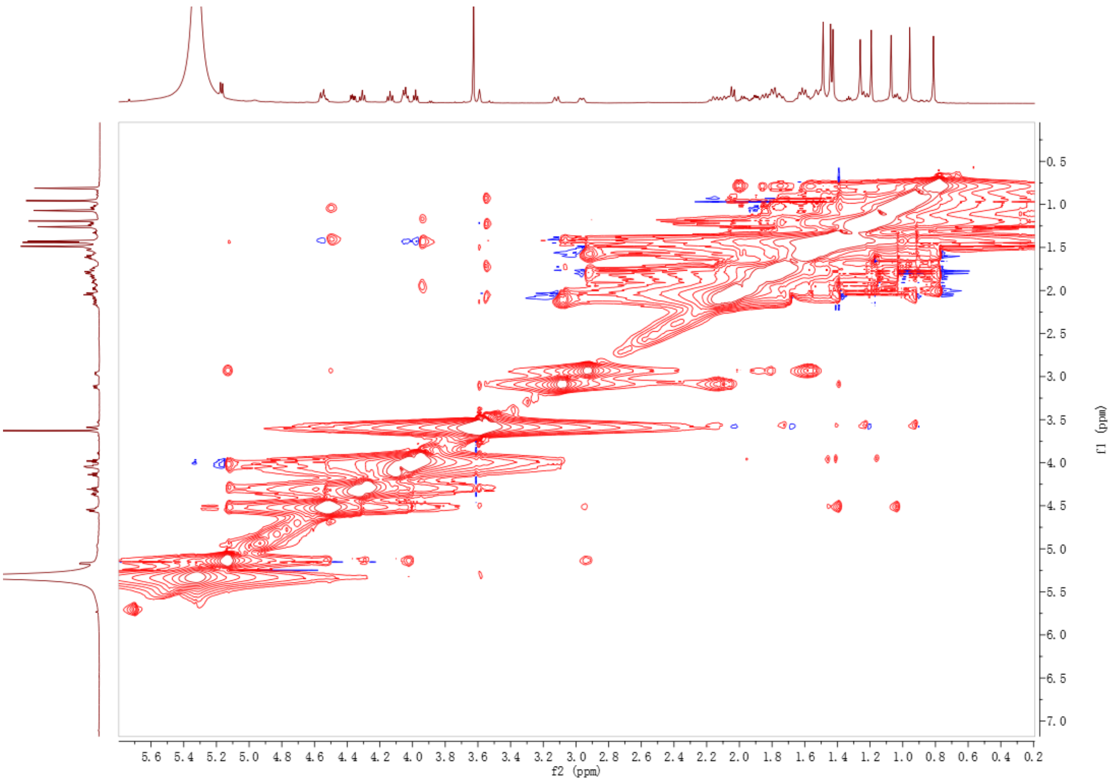

Figure S24. ROESY spectrum of compound **3**

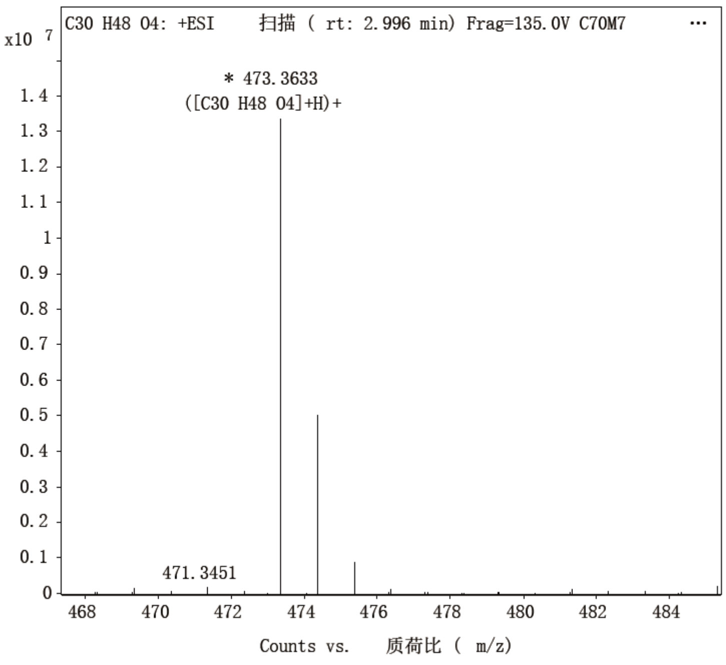

Figure S25. HRESIMS spectrum of compound **4**

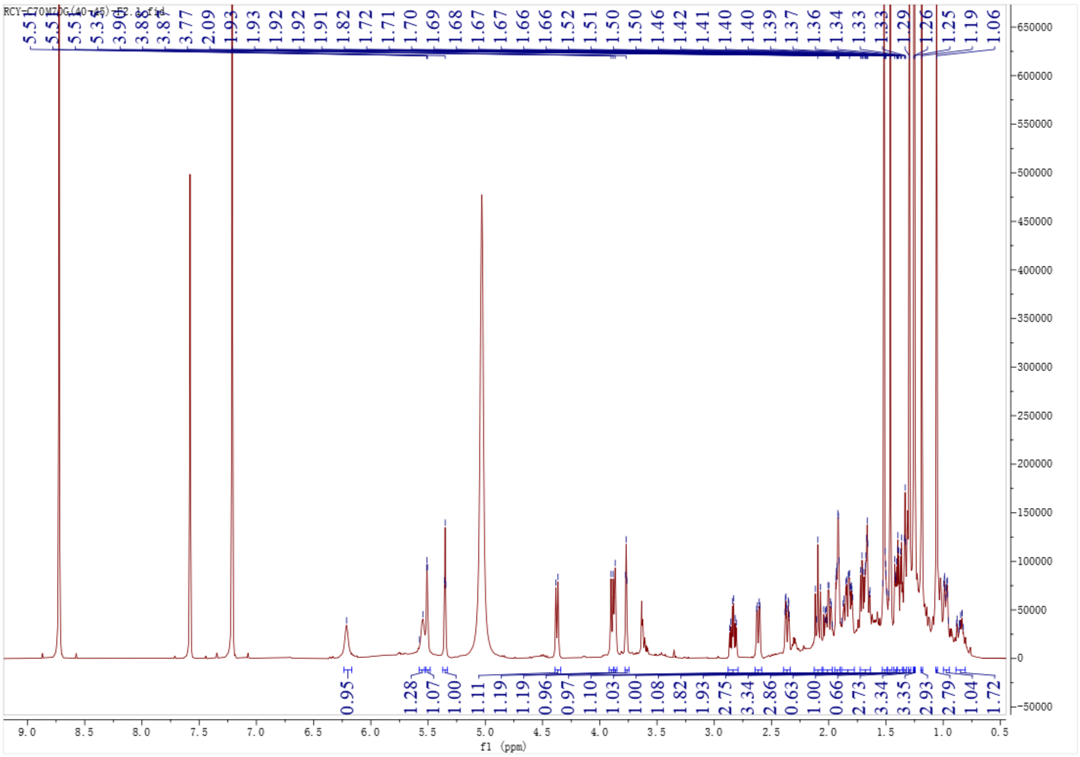

Figure S26. ^{1}H NMR (600 MHz, pyridine-d_5) spectrum of compound **4**

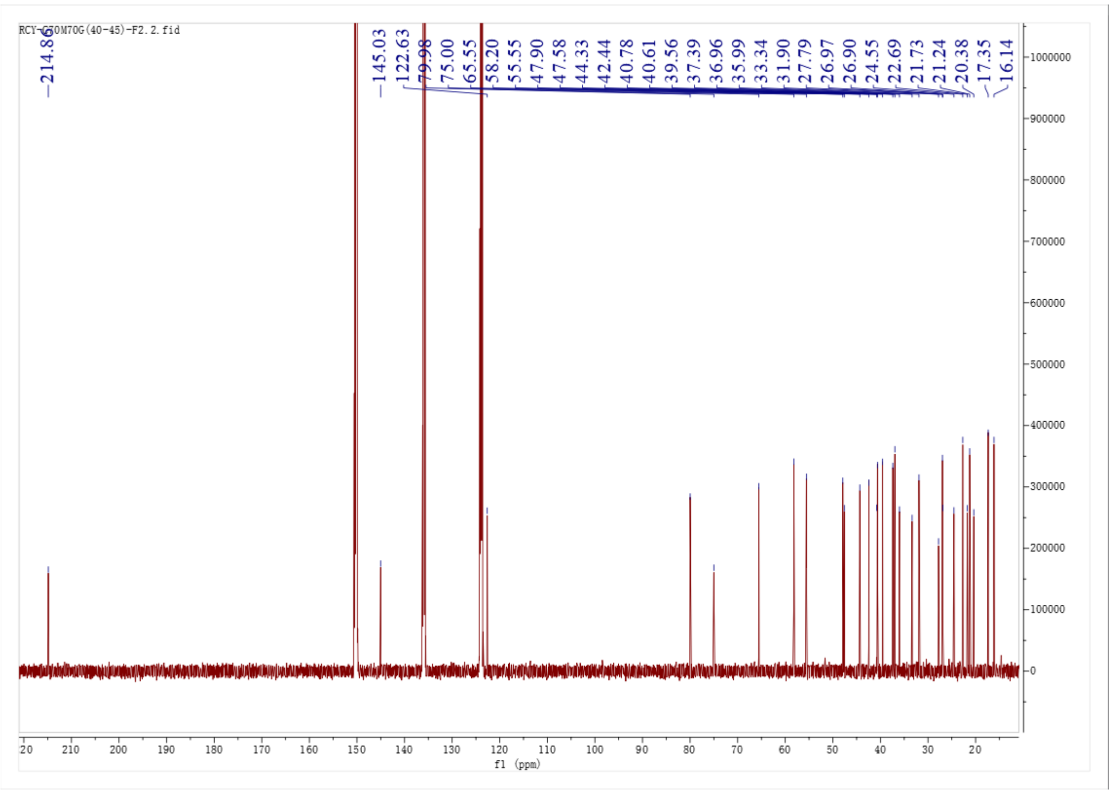

Figure S27. ^{13}C NMR (150 MHz, pyridine-d_5) spectrum of compound **4**

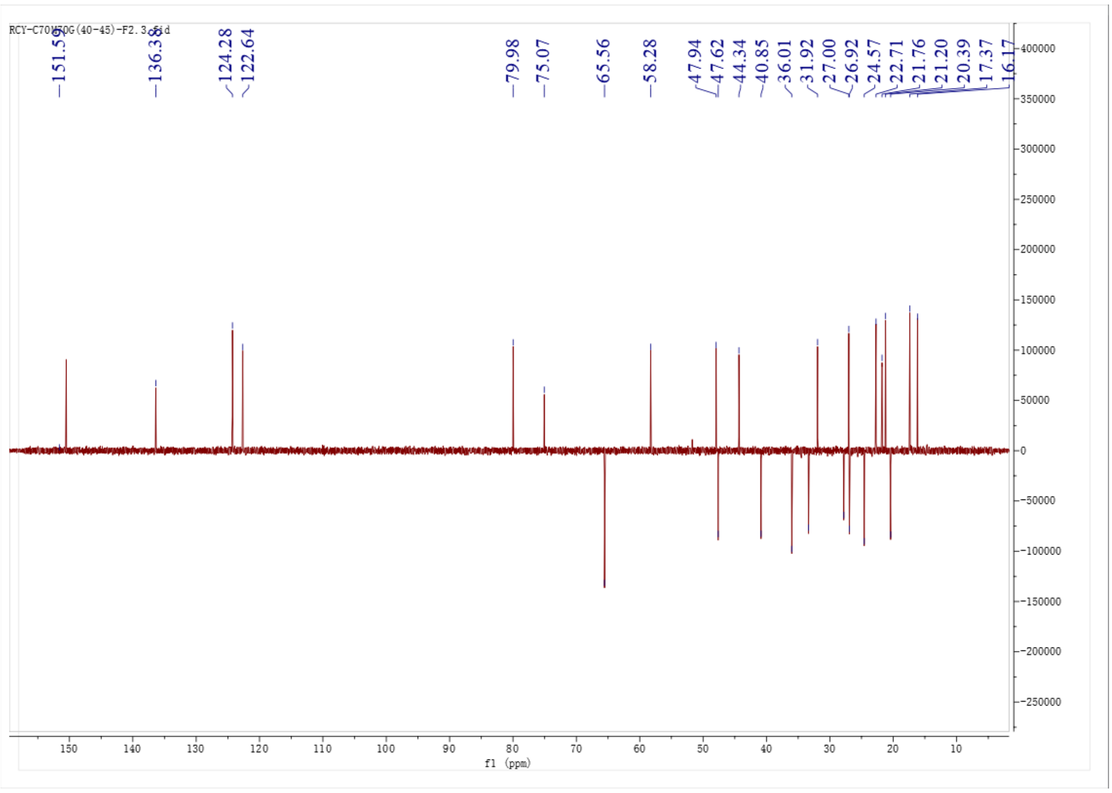

Figure S28. DEPT (150 MHz, pyridine-d_5) spectrum of compound **4**

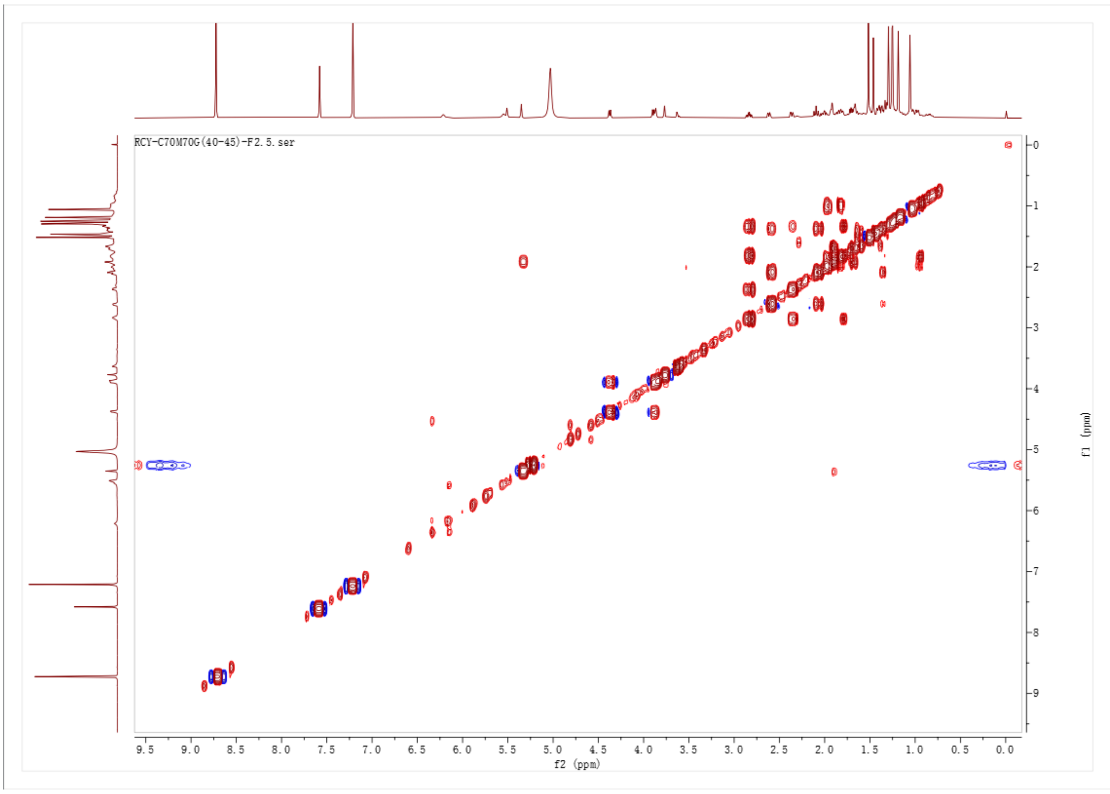

Figure S29. ^1H-^1H COSY (600 MHz, pyridine-d_5) spectrum of compound **4**

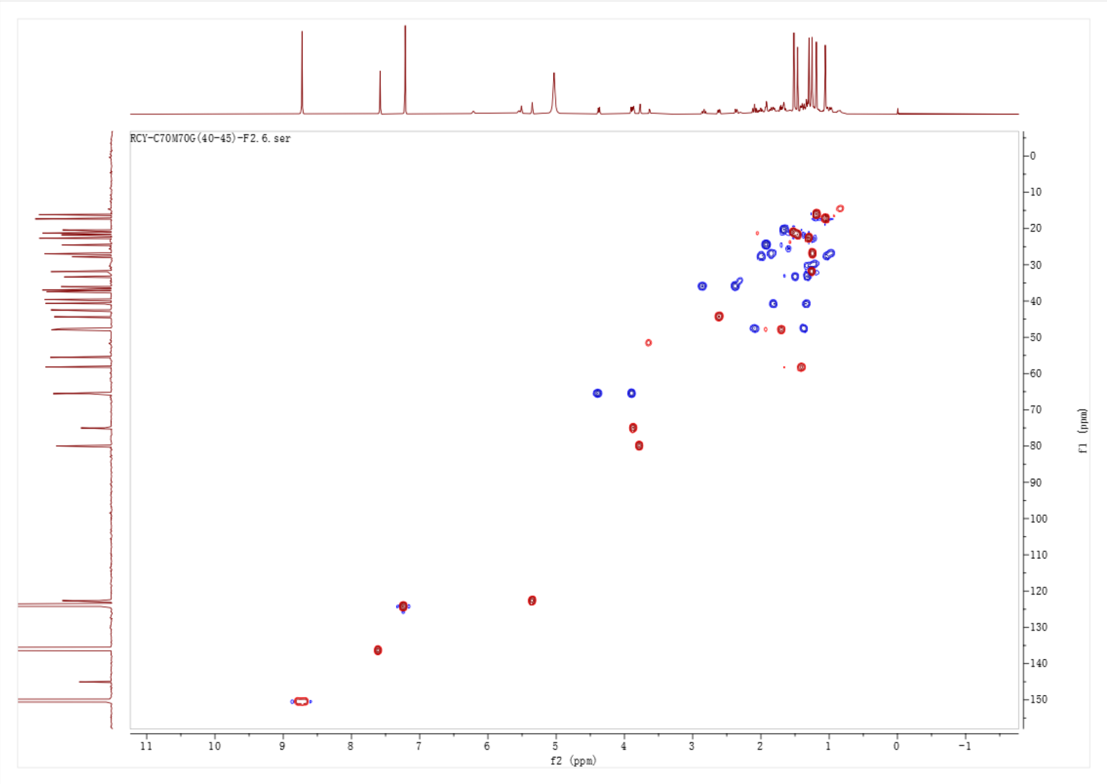

Figure S30. HSQC spectrum of compound **4**

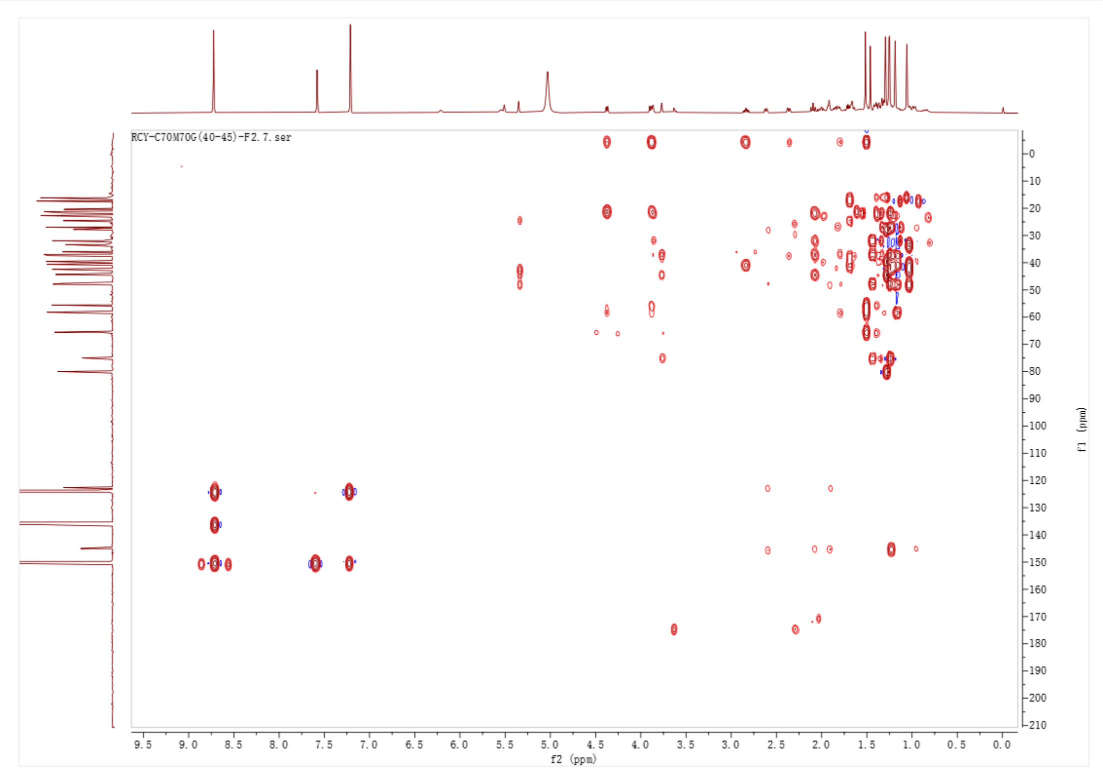

Figure S31. HMBC spectrum of compound **4**

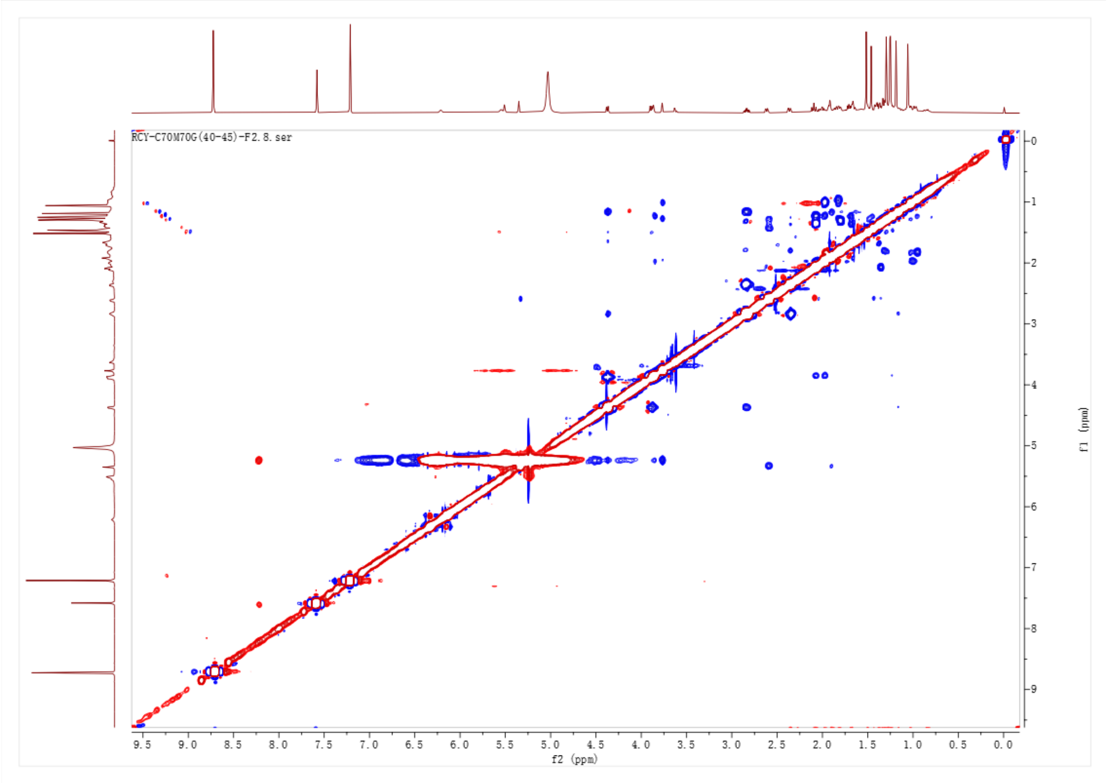

Figure S32. NOESY spectrum of compound **4**

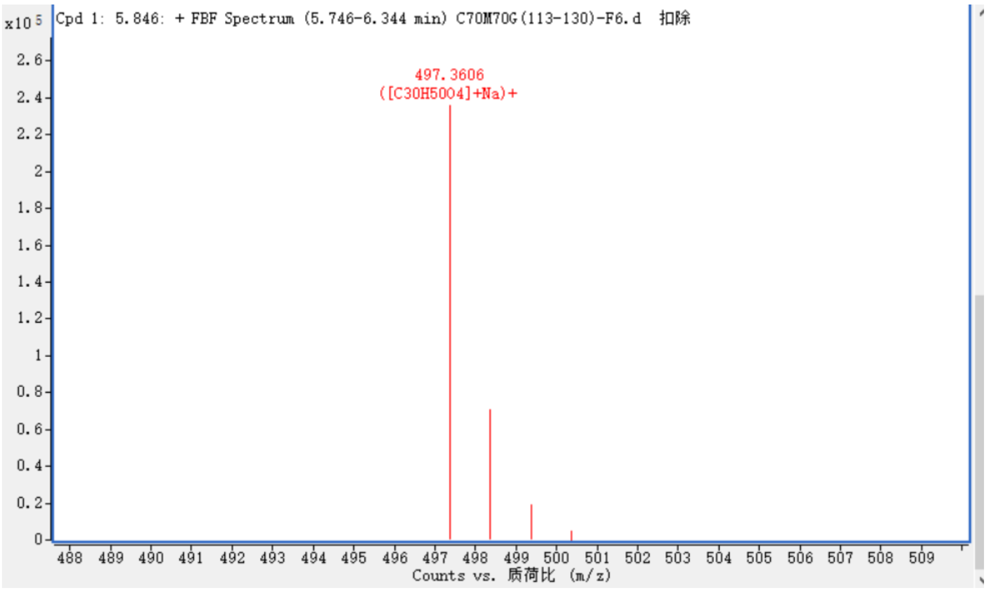

Figure S33. HRESIMS spectrum of compound **5**

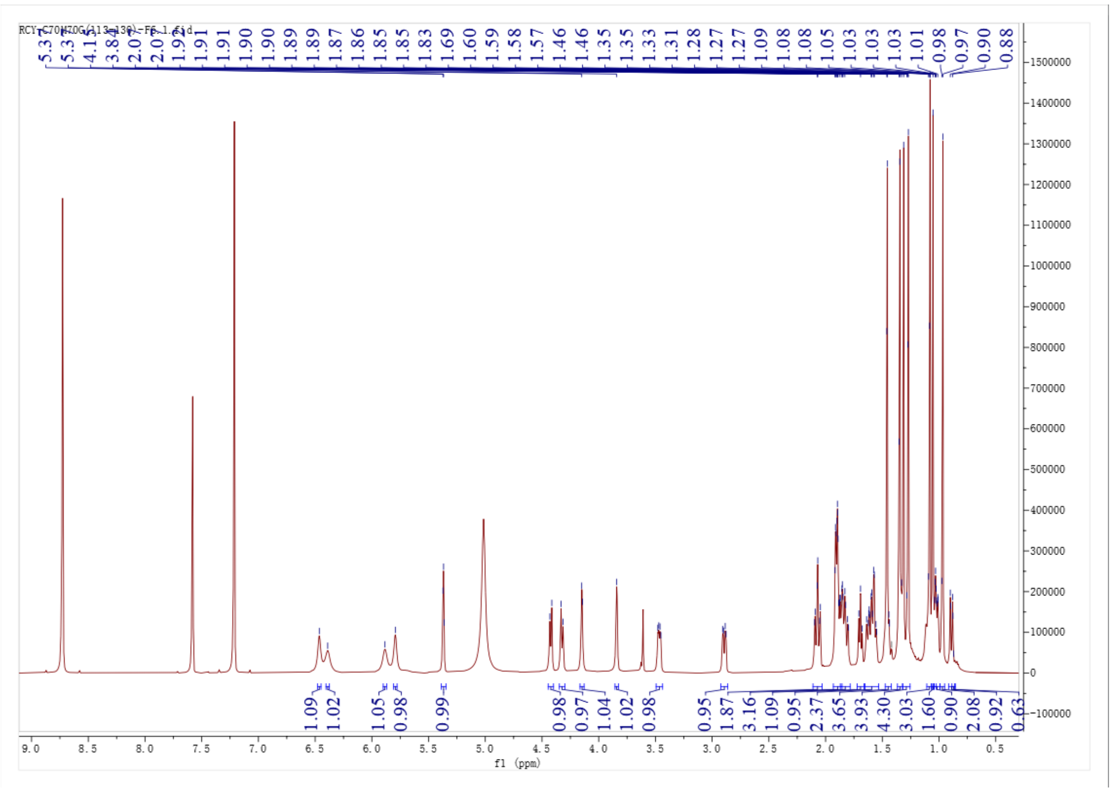

Figure S34. ^{1}H NMR (600 MHz, pyridine-d_5) spectrum of compound **5**

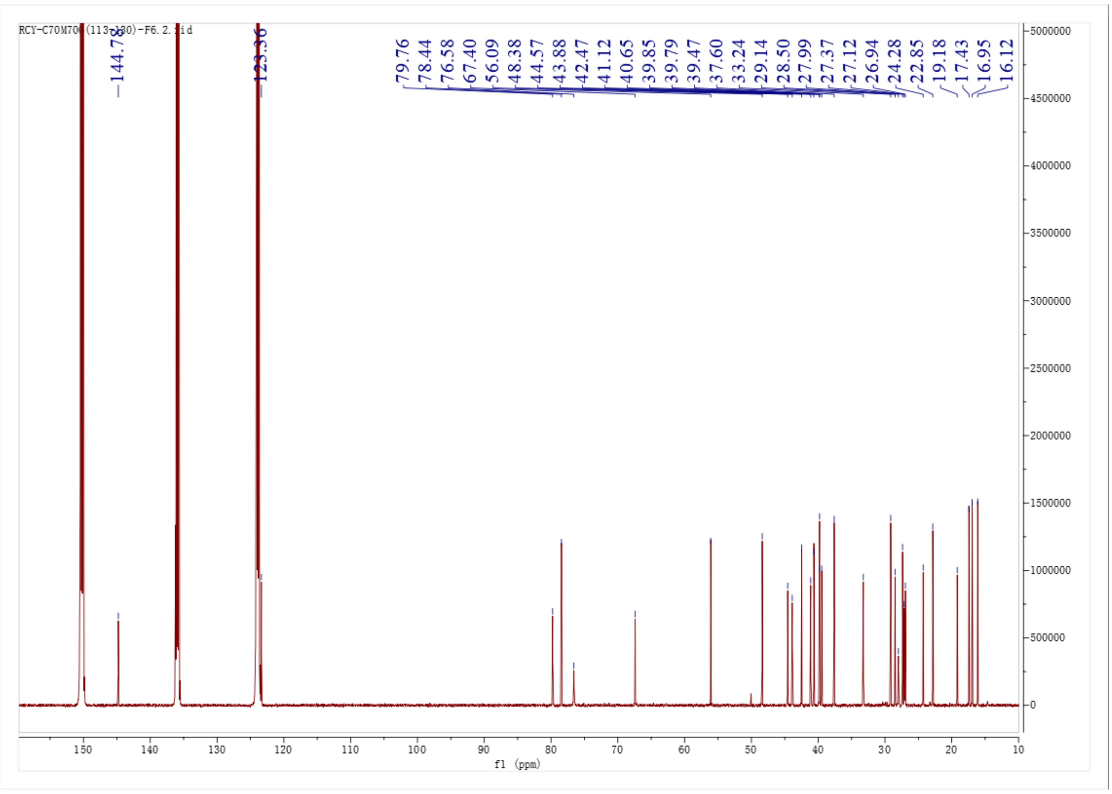

Figure S35. ^{13}C NMR (150 MHz, pyridine-d_5) spectrum of compound **5**

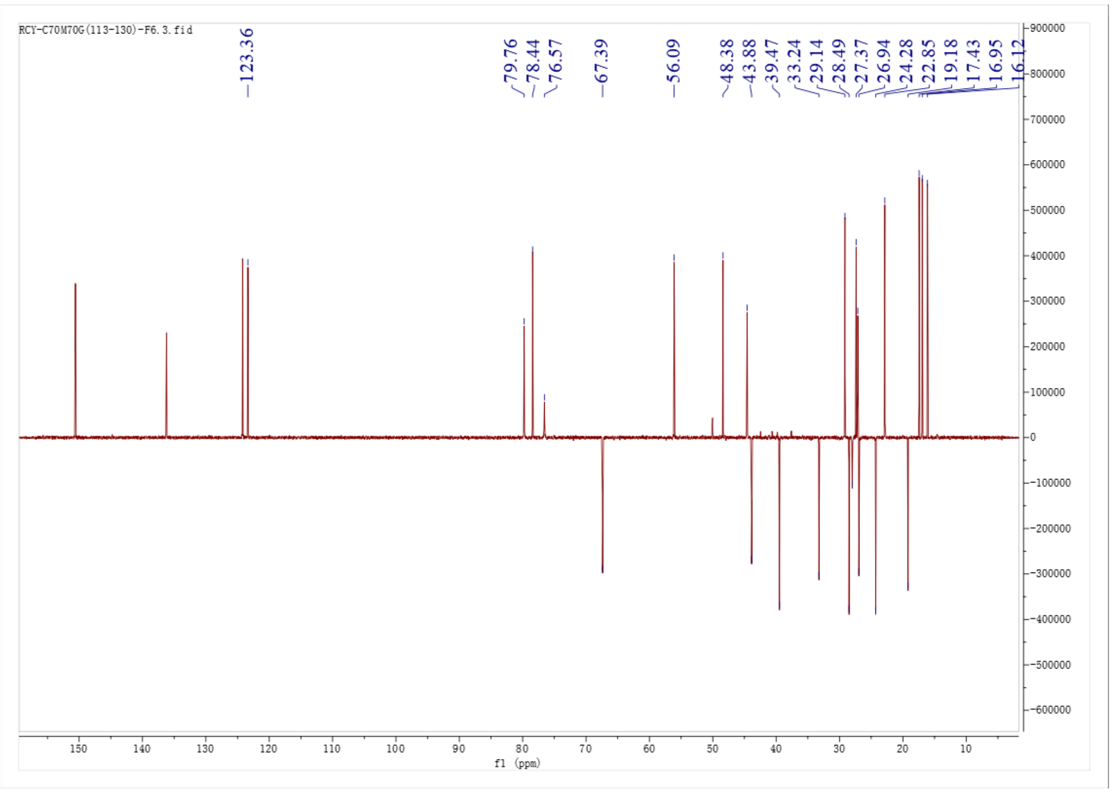

Figure S36. DEPT (150 MHz, pyridine-d_5) spectrum of compound **5**

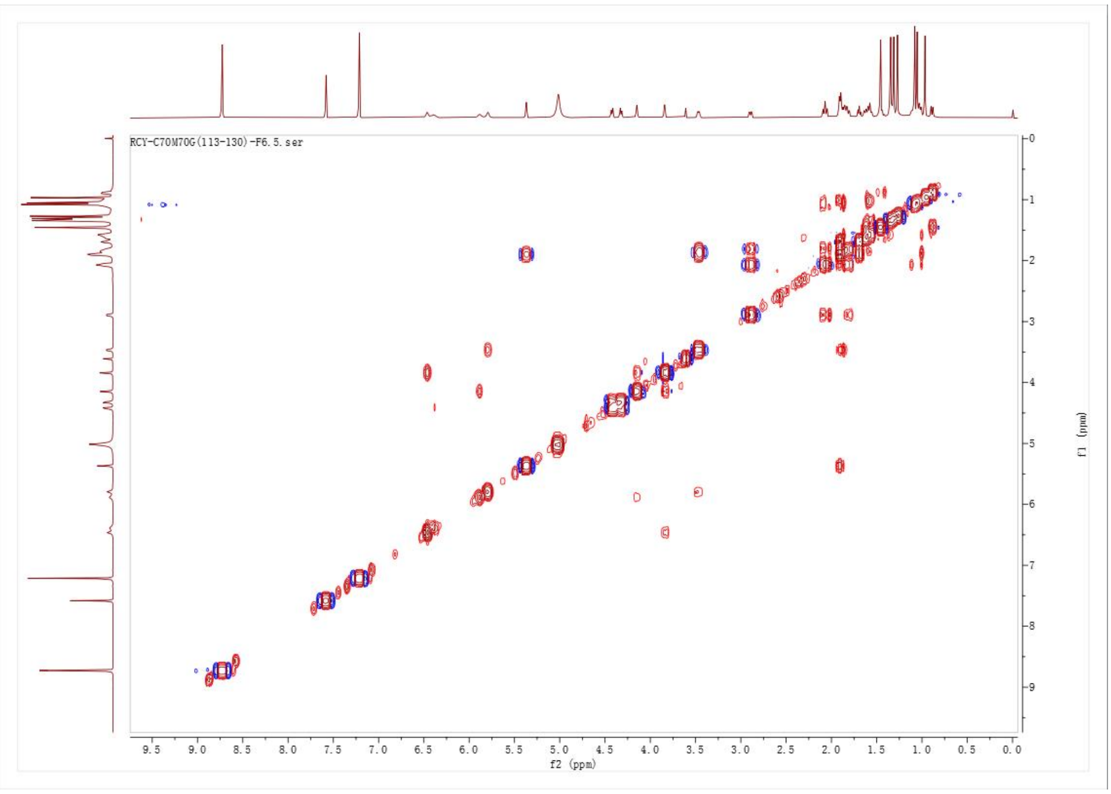

Figure S37. ¹H-¹H COSY (600 MHz, pyridine-d_5) spectrum of compound **5**

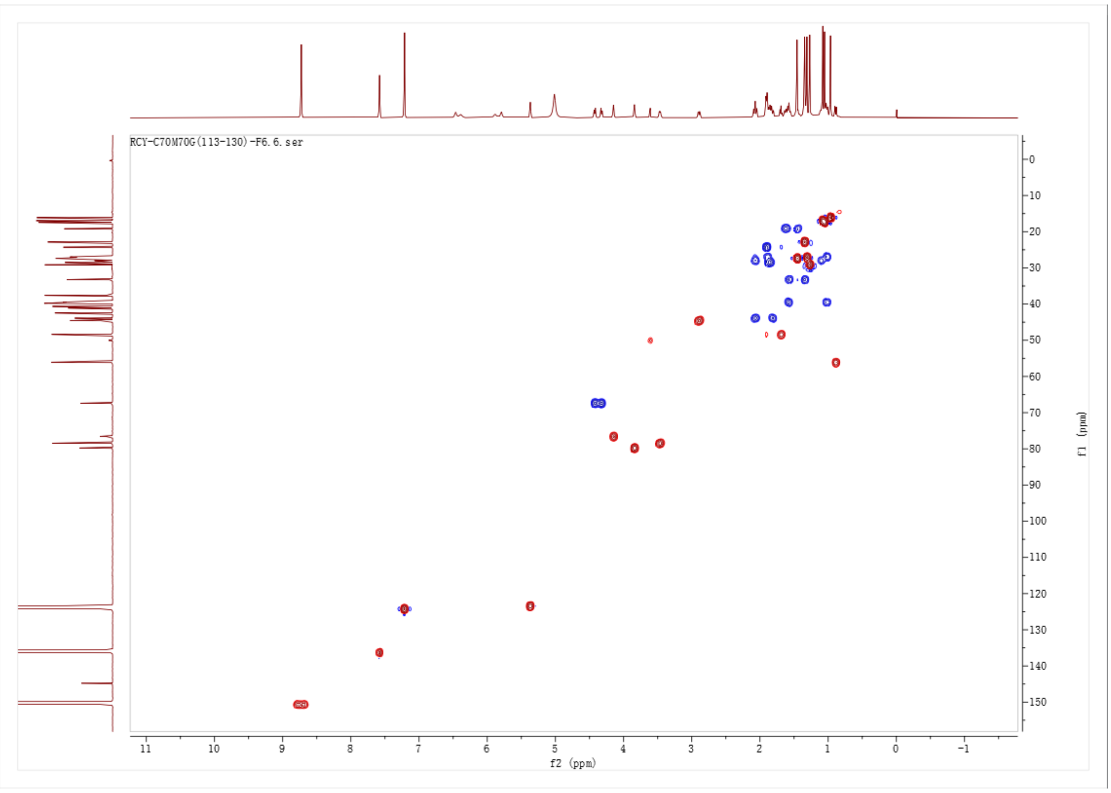

Figure S38. HSQC spectrum of compound **5**

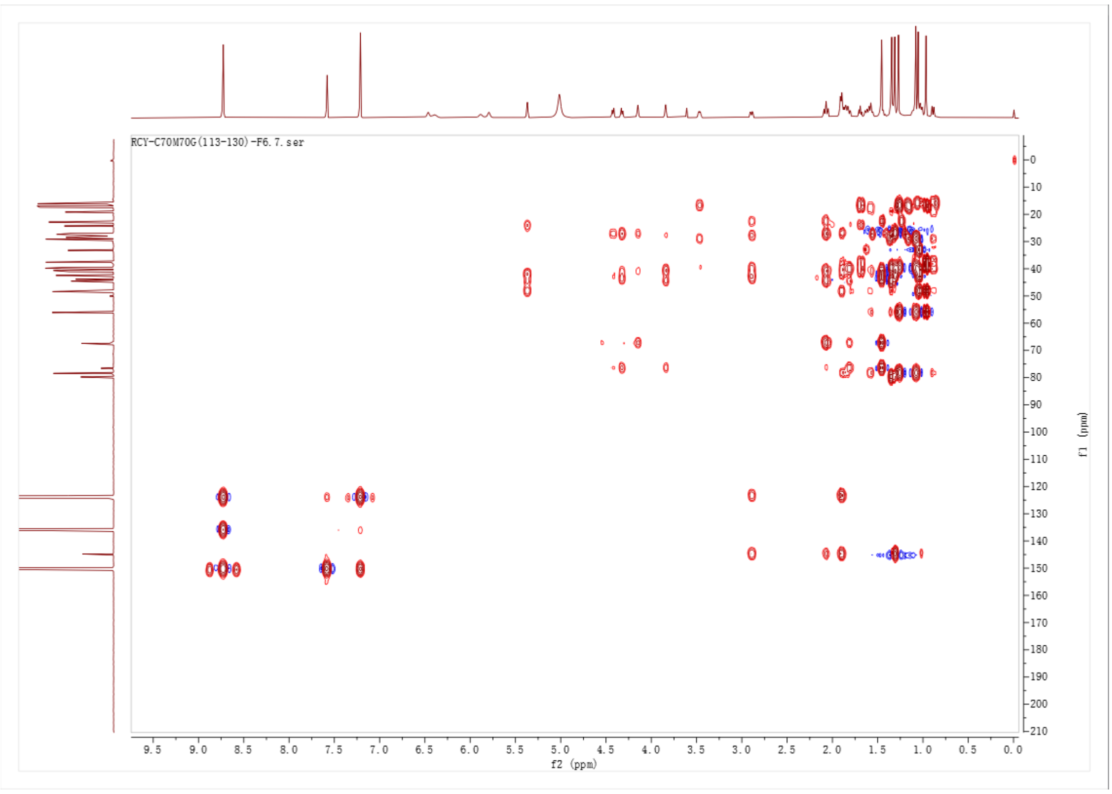

Figure S39. HMBC spectrum of compound **5**

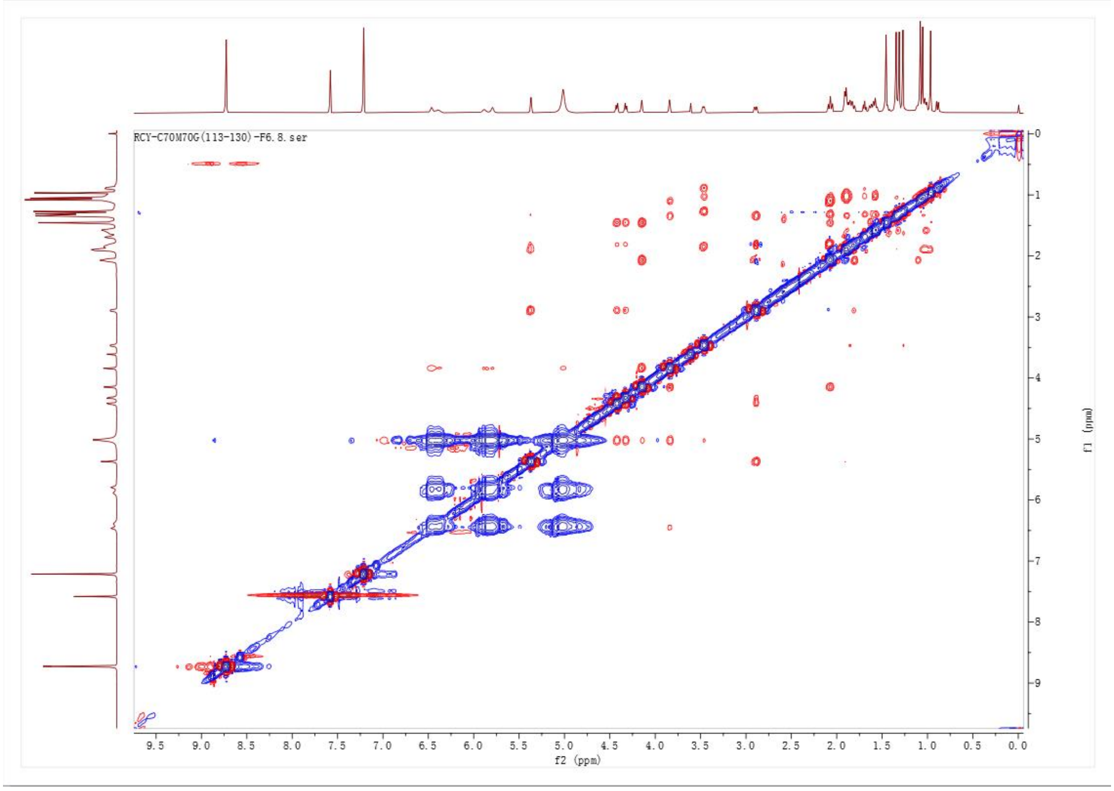

Figure S40. NOESY spectrum of compound **5**

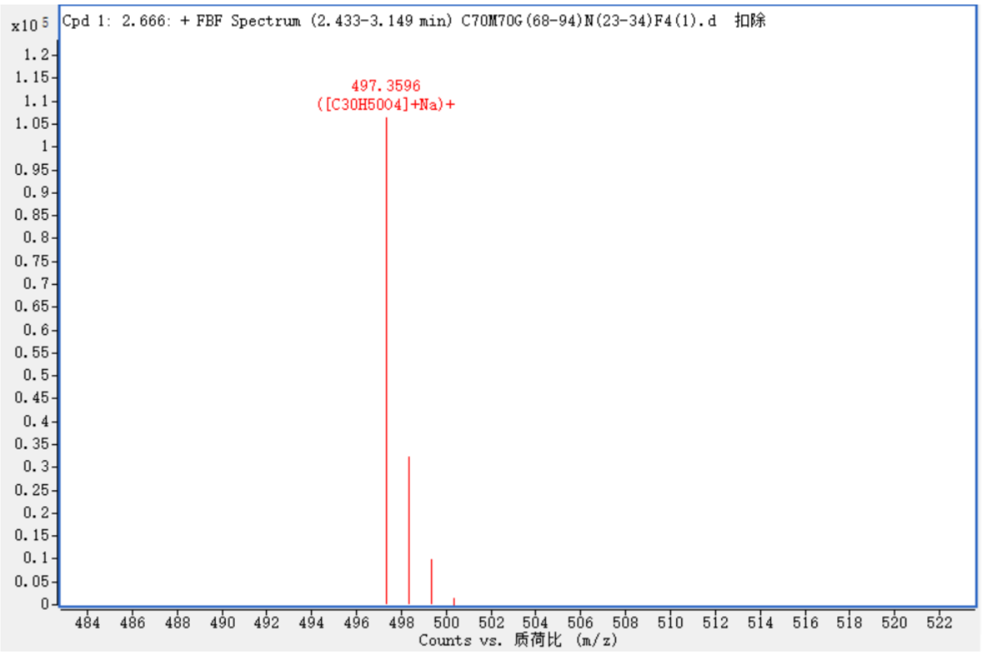

Figure S41. HRESIMS spectrum of compound **6**

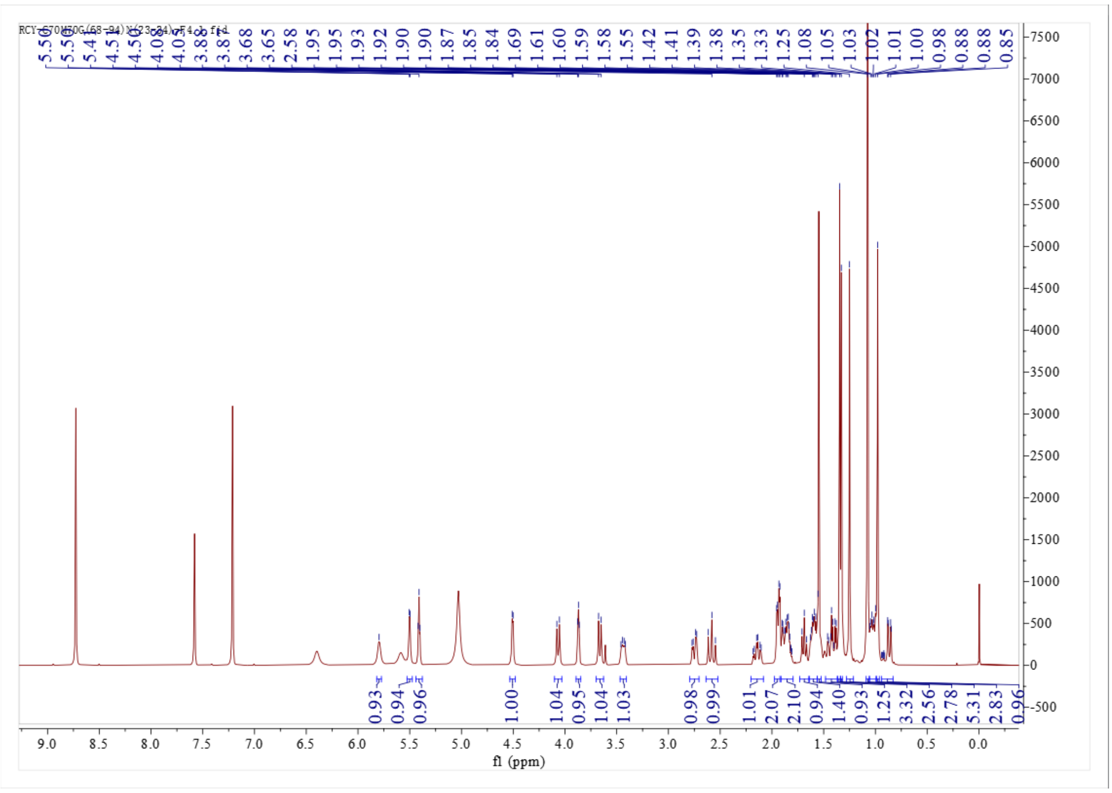

Figure S42. ^{1}H NMR (600 MHz, pyridine-d_5) spectrum of compound **6**

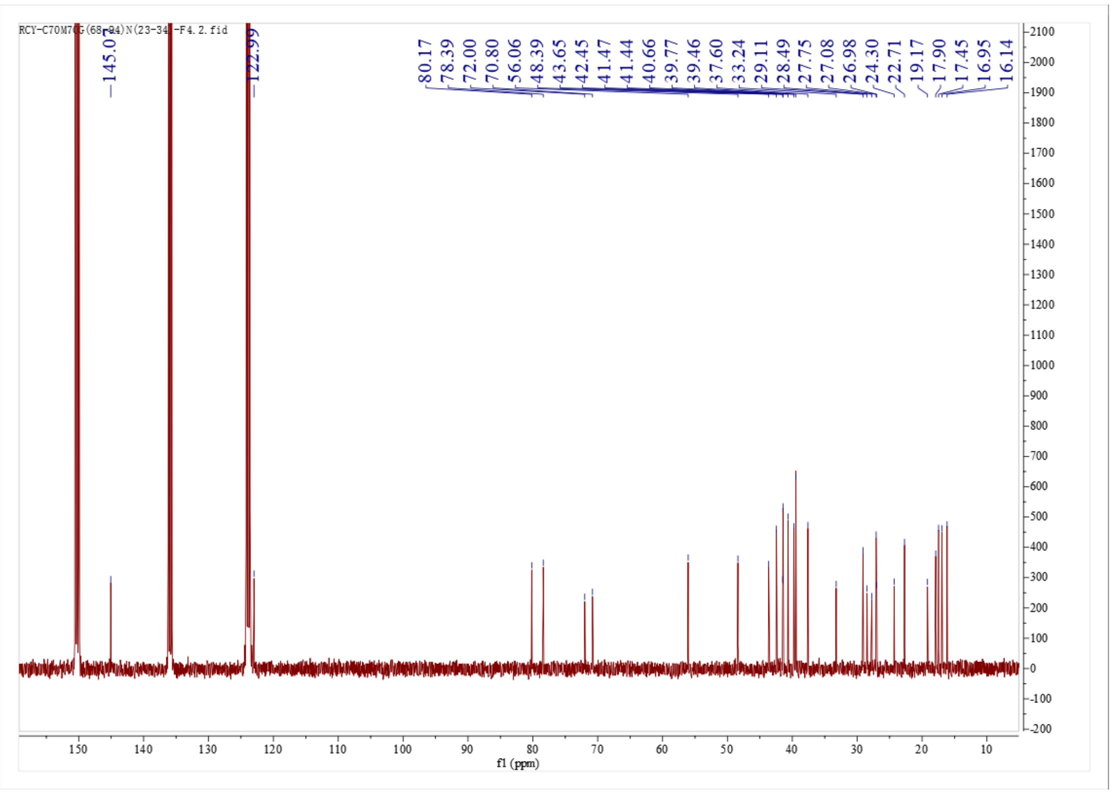

Figure S43. ^{13}C NMR (150 MHz, pyridine-d_5) spectrum of compound **6**

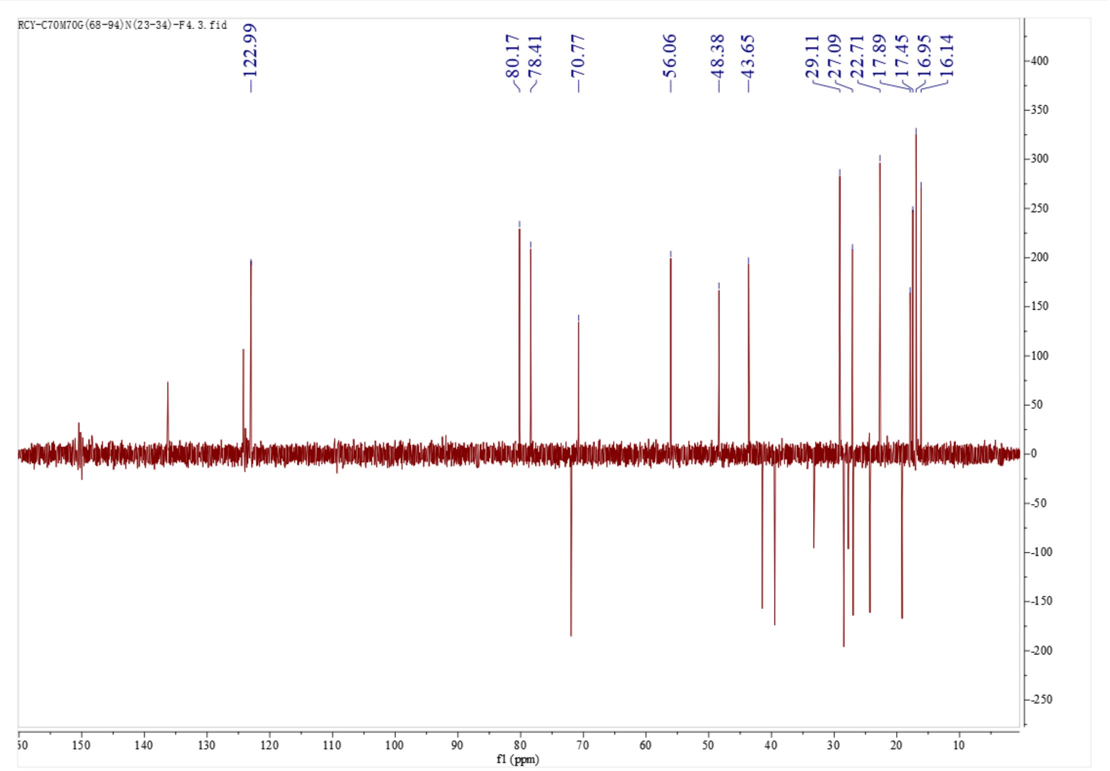

Figure S44. DEPT (150 MHz, pyridine-d_5) spectrum of compound **6**

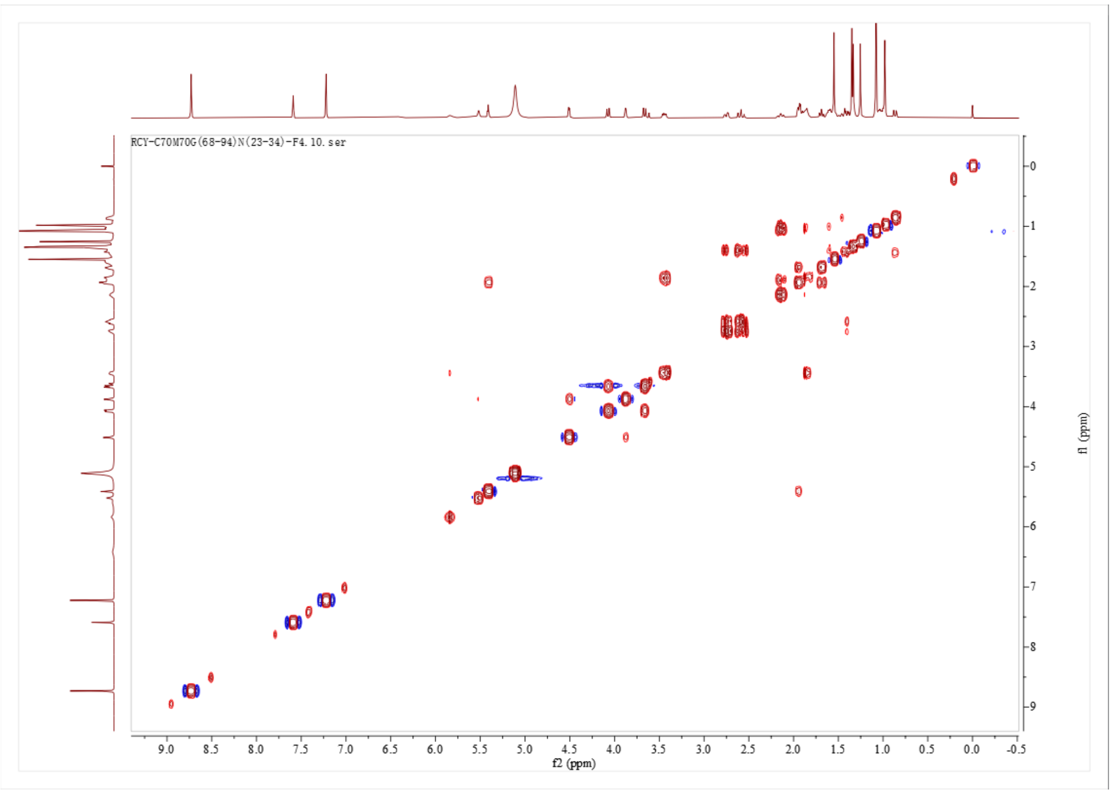

Figure S45. ^{1}H-^{1}H COSY (600 MHz, pyridine-d_5) spectrum of compound **6**

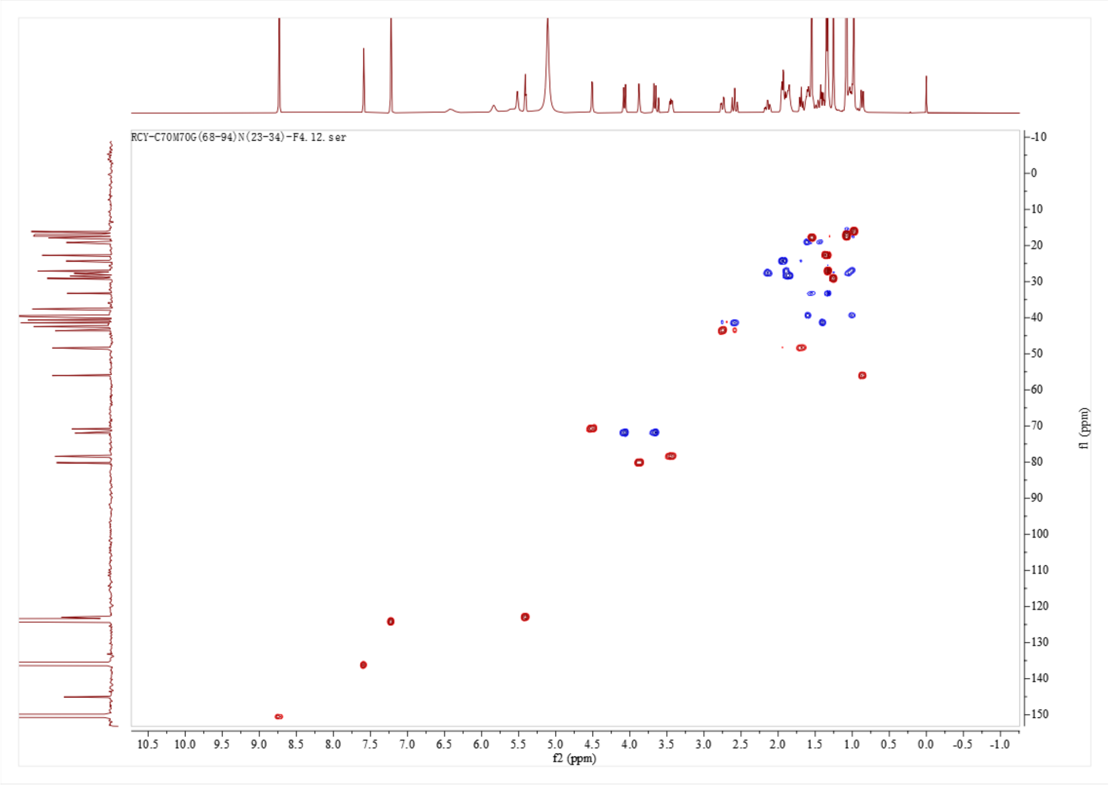

Figure S46. HSQC spectrum of compound **6**

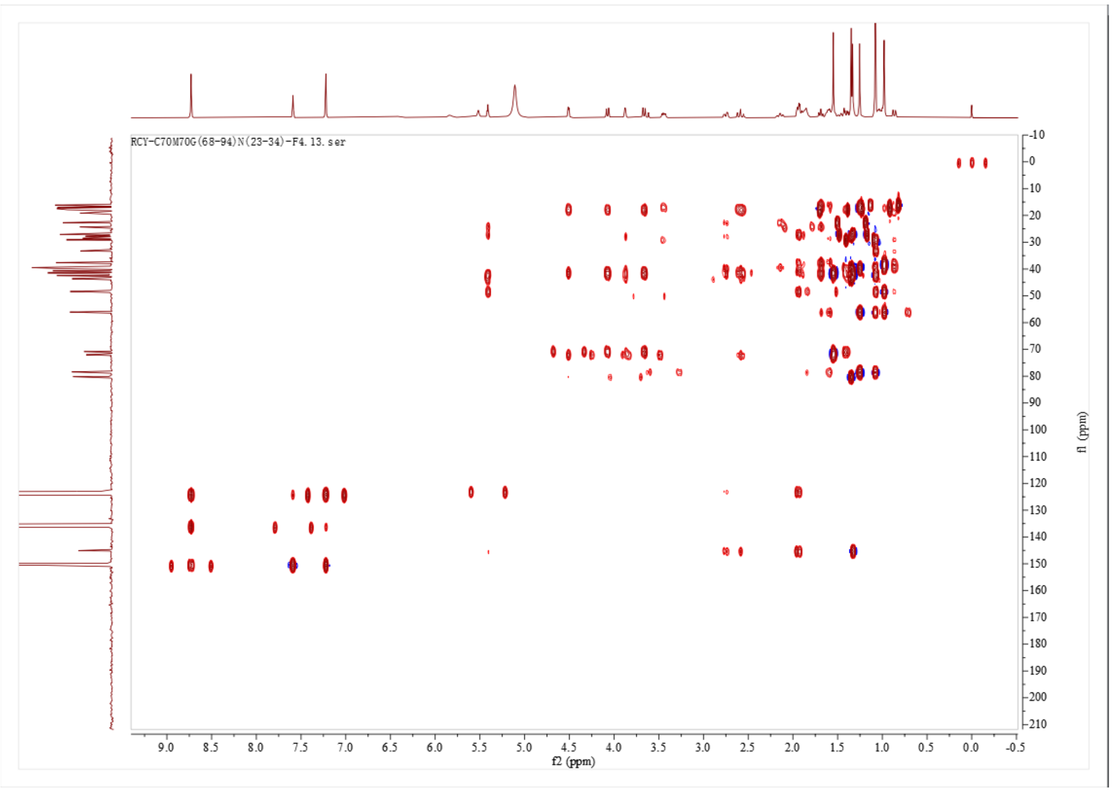

Figure S47. HMBC spectrum of compound **6**

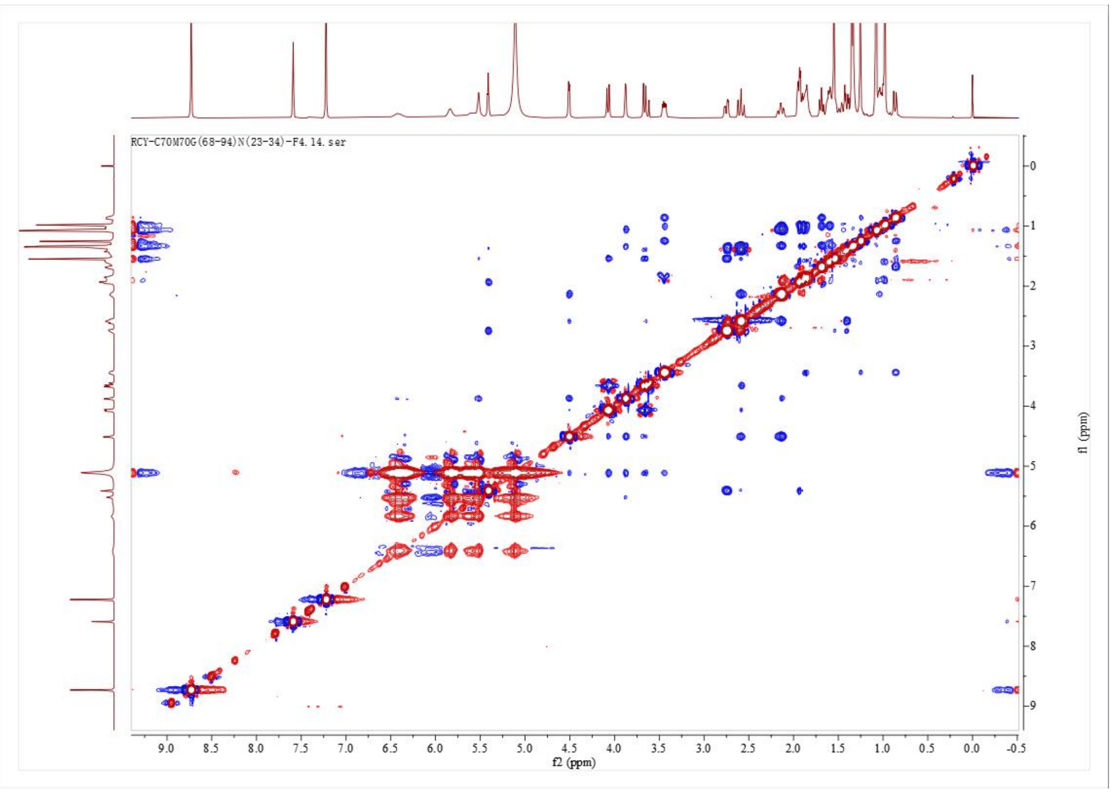

Figure S48. NOESY spectrum of compound **6**

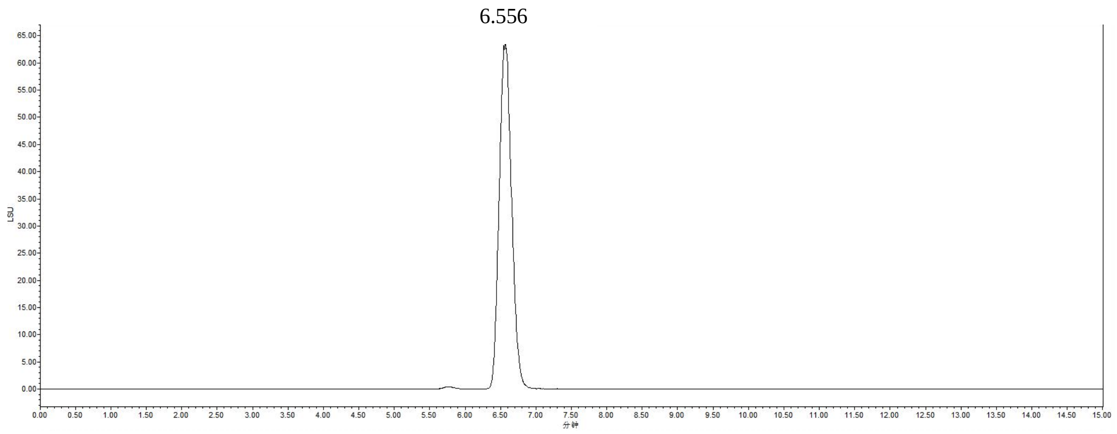

Figure S49. The HPLC plot of the standard and **3** of D-glucopyranose

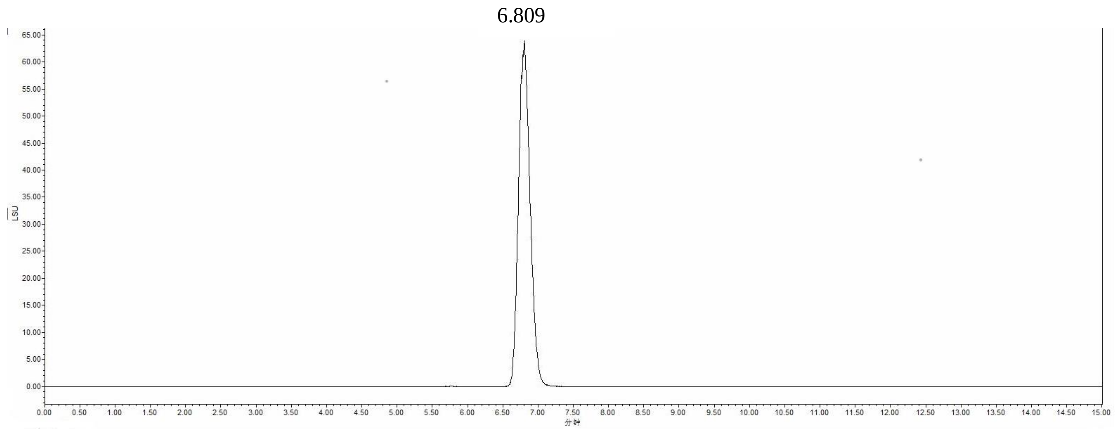

Figure S50. The HPLC plot of the standard of L-arabinose

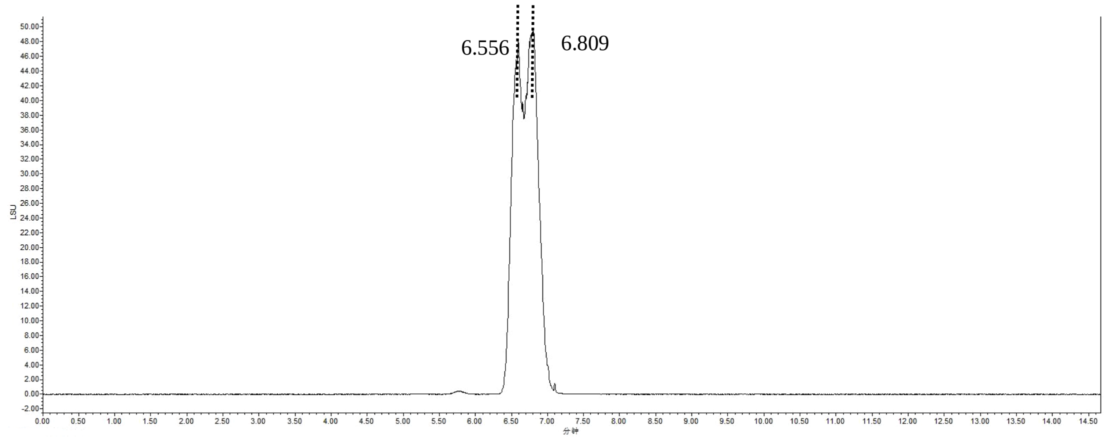

Figure S51. The HPLC plot of the compound **1** and **2** of D-glucopyranose and L-arabinose

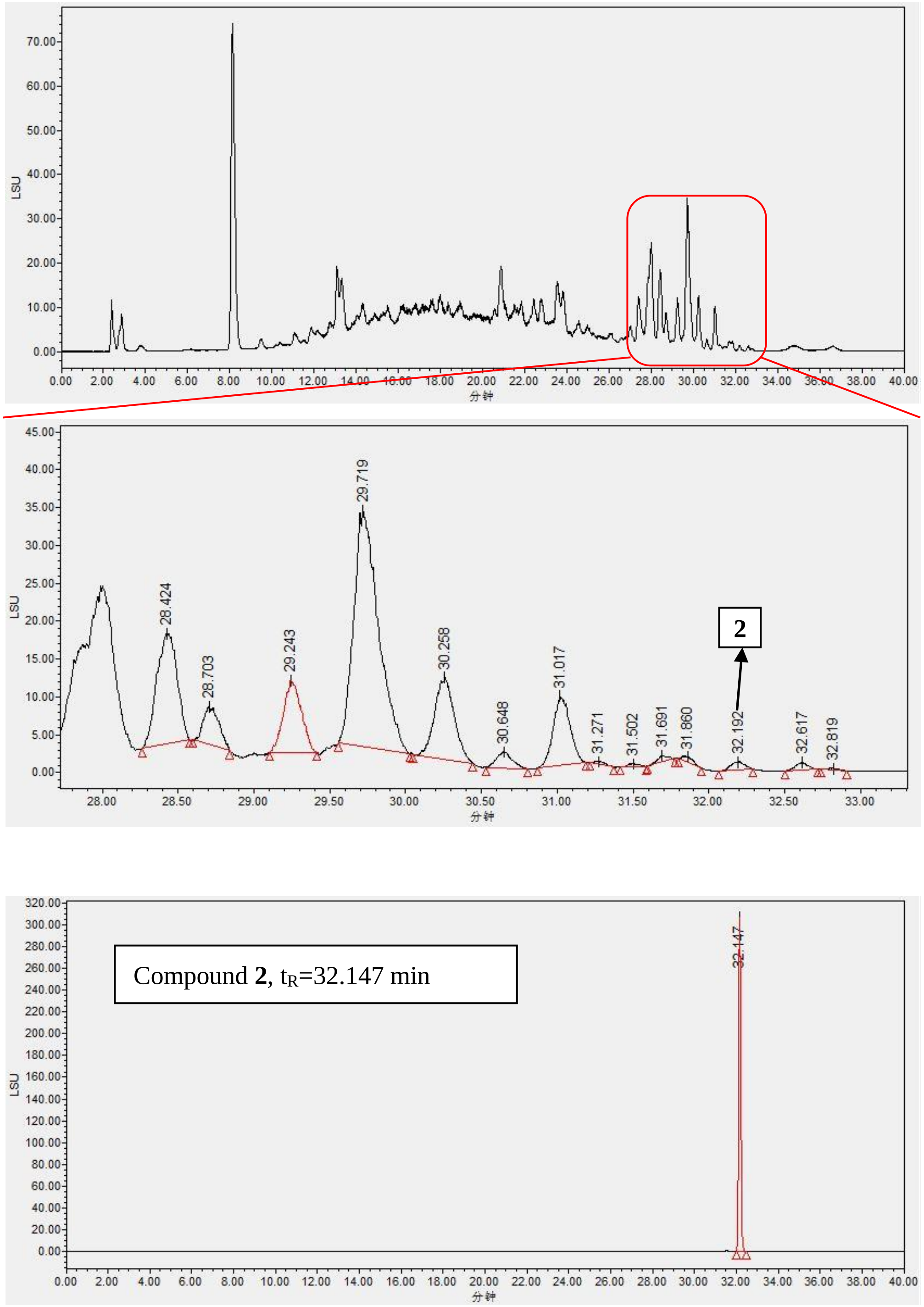

Figure S52. The HPLC plots of the initial extract of *H. littoralis* and pure compound **2**

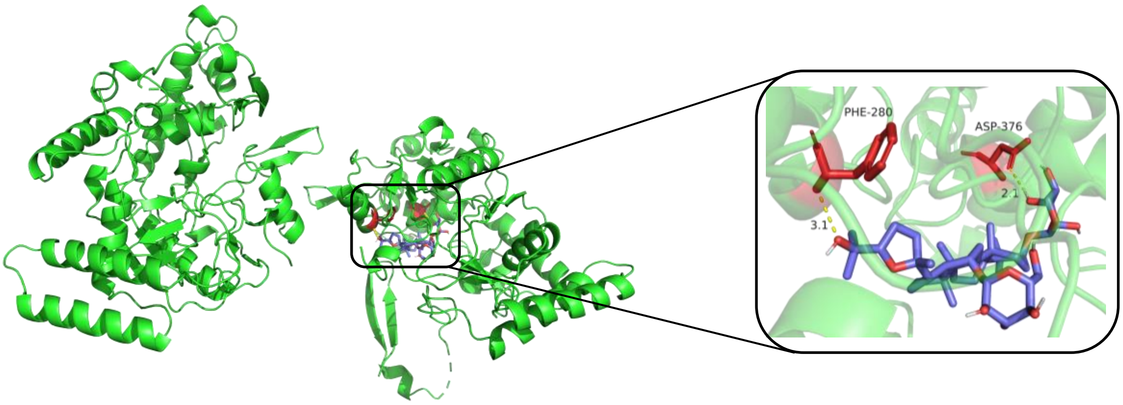

Figure S53. Molecular docking result of compound **1** with iNOS protein

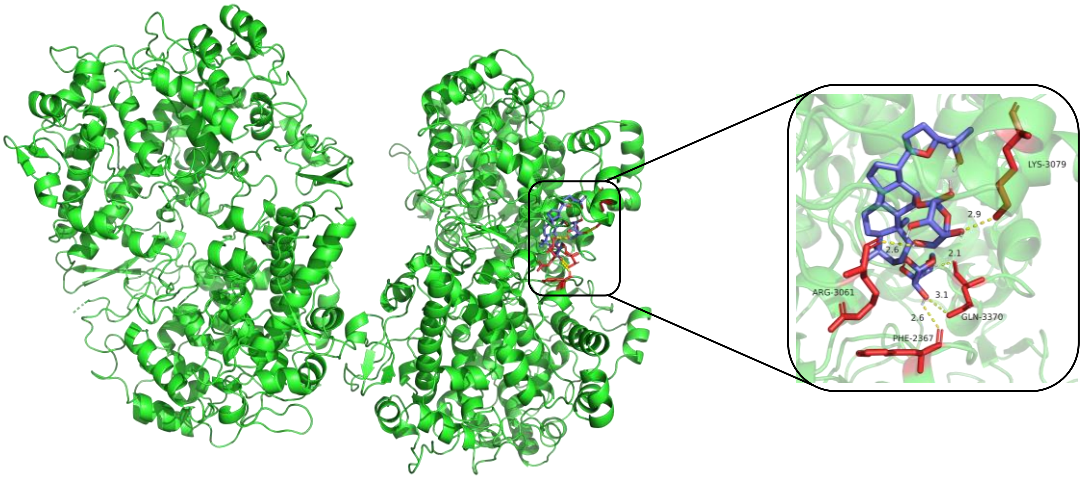

Figure S54. Molecular docking result of compound **1** with COX-2 protein

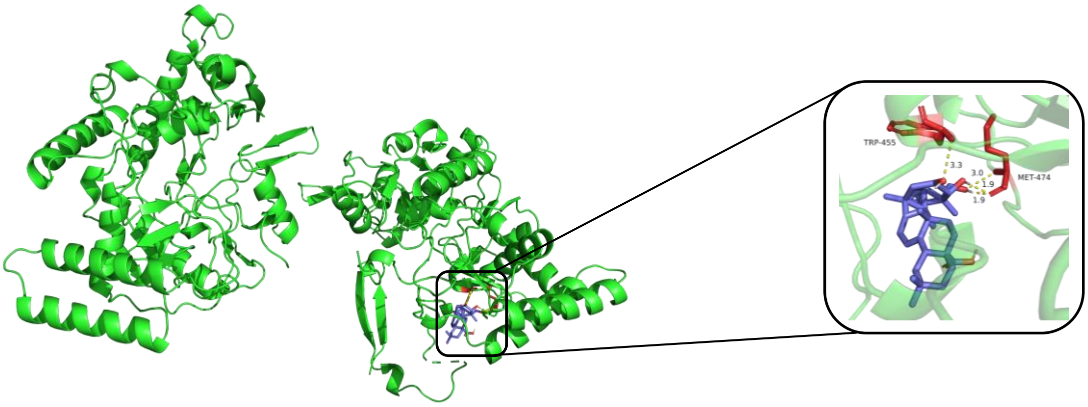

Figure S55. Molecular docking result of compound **12** with iNOS protein

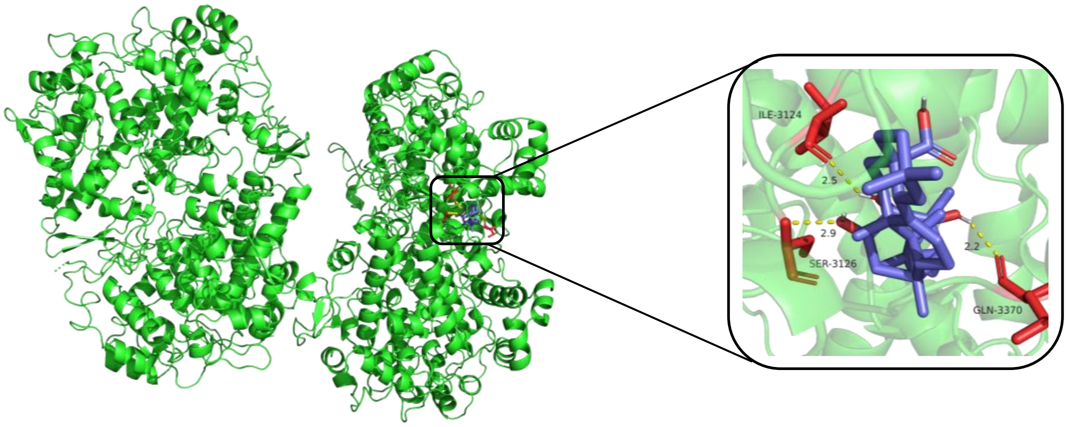

Figure S56. Molecular docking result of compound **12** with COX-2 protein

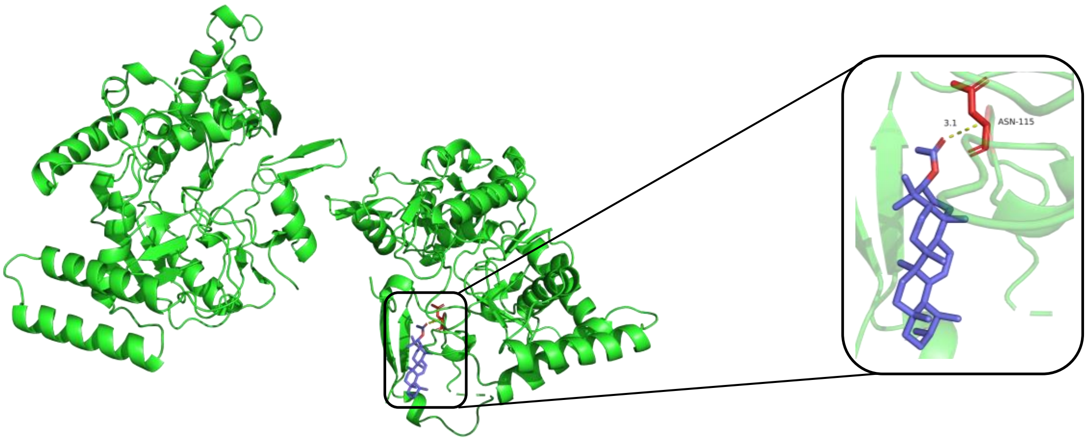

Figure S57. Molecular docking result of compound **16** with iNOS protein

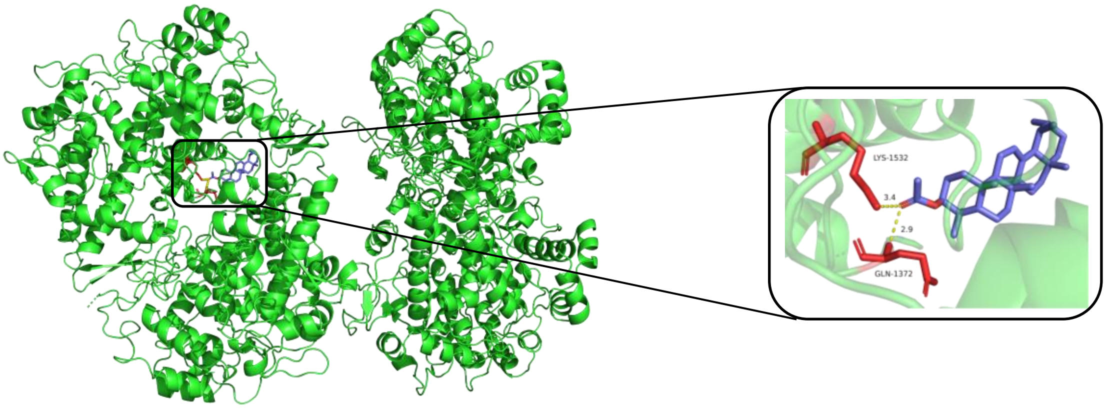

Figure S58. Molecular docking result of compound **16** with COX-2 protein

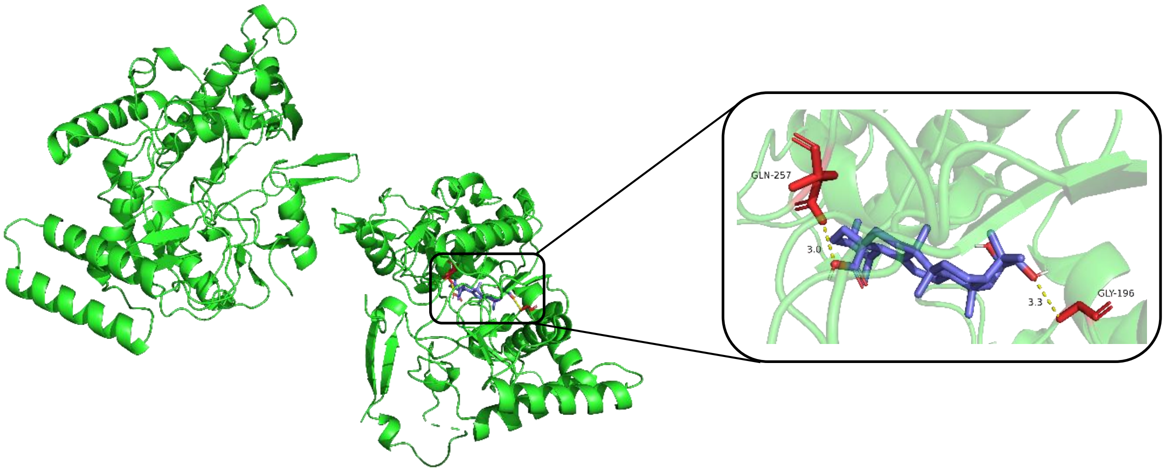

Figure S59. Molecular docking result of compound **17** with iNOS protein

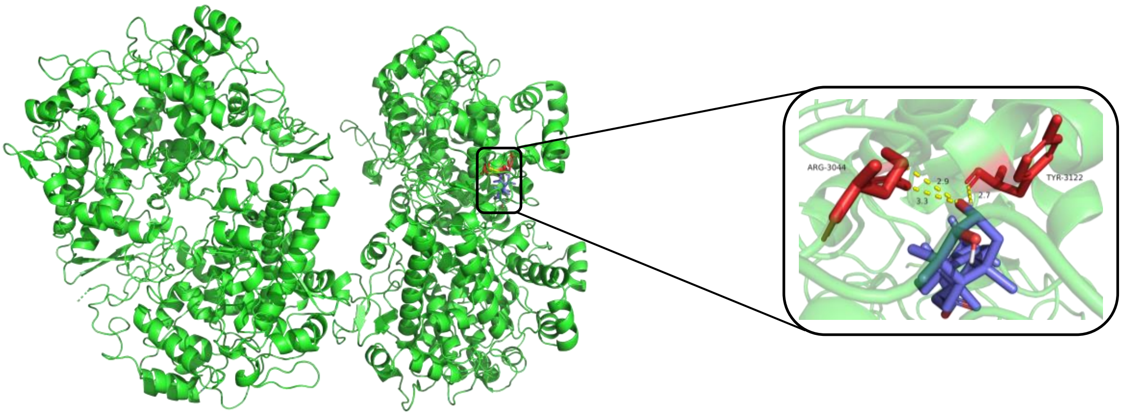

Figure S60. Molecular docking result of compound **17** with COX-2 protein

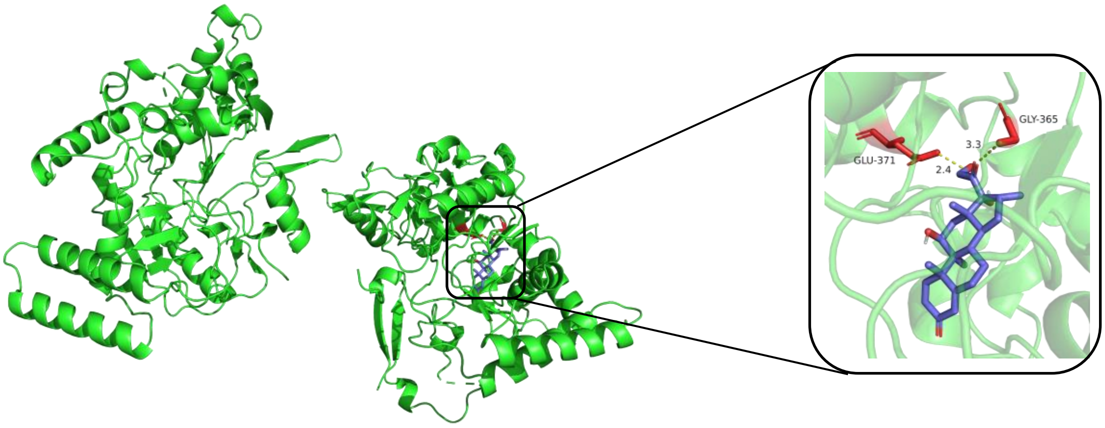

Figure S61. Molecular docking result of dexamethasone with iNOS protein

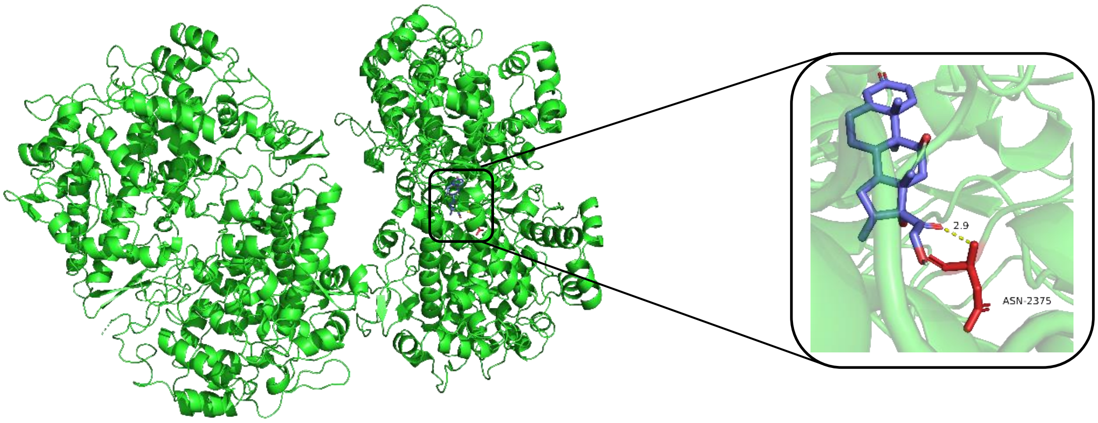

Figure S62. Molecular docking result of dexamethasone with COX-2 protein

Table S1. Logarithms of free binding energies (FBE, kcal/mol) of compounds **1**, **12**, **16**, **17**, **18**, and Dexamethasone to the active cavities of iNOS (PDB code: 3E6T) and targeting residues of the binding site located on the mobile flap

Compound	−Log (FBE)	Targeting residues
**1**	−9.6	PHE-280, ASP-376
**12**	−8.1	MET-474, TRP-455
**16**	−8.0	ASN-115
**17**	−8.8	GLN-257, GLY-196
**18**	−9.0	TRP-457, GLY-365
**Dexamethasone**	−9.5	GLU-371, GLY-365

Table S2. Logarithms of free binding energies (FBE, kcal/mol) of compounds **1**, **12**, **16**, **17**, **18**, and Dexamethasone to the active cavities of COX-2 (PDB code: 1PXX) and targeting residues of the binding site located on the mobile flap

Compound	−Log (FBE)	Targeting residues
**1**	−9.3	GLN-3370, PHE-2367, ARG-3061, LYS-3079
**12**	−9.8	GLN-3370, SER-3126, ILE-3124
**16**	−8.5	GLN-1372, LYS-1532
**17**	−10.3	ARG-3044, TYR-3122
**18**	−9.0	ARG-1044, GLN-1370
**Dexamethasone**	−9.6	ASN-2375
